# Declining hip fracture burden in Sweden 1998–2019 and consequences for projections through 2050

**DOI:** 10.1038/s41598-024-51363-6

**Published:** 2024-01-06

**Authors:** Karl Michaëlsson, John A. Baron, Liisa Byberg, Susanna C. Larsson, Håkan Melhus, Rolf Gedeborg

**Affiliations:** 1 Department of Surgical Sciences, Uppsala University, 751 85 Uppsala, Sweden; 2 Department of Medicine, University of North Carolina School of Medicine, Chapel Hill, NC USA; 3 Department of Epidemiology, Gillings School of Global Public Health, University of North Carolina, Chapel Hill, NC USA; 4 Unit of Cardiovascular and Nutritional Epidemiology, Institute of Environmental Medicine, Karolinska Institutet, Stockholm, Sweden; 5 Department of Medical Sciences, Uppsala University, Uppsala, Sweden

**Keywords:** Epidemiology, Geriatrics

## Abstract

We aimed to estimate the absolute and age-standardized number of hip fractures in Sweden during the past two decades to produce time trends and future projections. We used nationwide register data from 1998 to 2019 and a validated algorithm to calculate the annual absolute and age-standardized number of incident hip fractures over time. The total hip fracture burden was 335,399 incident events over the 22 years, with a change from 16,180 in 1998 to 13,929 in 2019, a 14% decrease. One decade after the index hip fracture event, 80% of the patients had died, and 11% had a new hip fracture. After considering the steady growth of the older population, the decline in the age-standardized number of hip fractures from 1998 through 2019 was 29.2% (95% CI 28.1–30.2%) in women and 29.3% (95% CI 27.5–30.7%) in men. With a continued similar reduction in hip fracture incidence, we can predict that 14,800 hip fractures will occur in 2034 and 12,000 in 2050 despite doubling the oldest old (≥ 80 years). Without an algorithm, a naïve estimate of the total number of hip fractures over the study period was 539,947, with a second 10-year hip fracture risk of 35%. We note an ongoing decline in the absolute and age-standardized actual number of hip fractures in Sweden, with consequences for future projections.

## Introduction

National medical databases and disease registers are crucial in determining disease burden and projecting future disease occurrence^1–3^. For such purposes, high completeness and coding accuracy of register data are crucial. Equally important is correctly identifying incident events, separating complications from the index event, identifying actual relapsing cases, and avoiding counting readmissions or hospital transfers as occurrences of new disease. Myocardial infarction^4^, stroke^5^, pneumonia^6^, injuries^7^, and fragility fractures^7^ are among several diseases that require careful algorithm application to avoid inaccurate estimates of disease burden.

Hip fracture is the most severe of all fragility fractures and almost always leads to hospitalization. This fracture is associated with significant morbidity, increased mortality, and high societal costs^8^, making it a priority for hospital registries. Worldwide, 1.6 million hip fractures are estimated to occur annually^8^ at an average of 80 years of age. They account for half of all fracture-related costs in aging populations^9,10^. With the nearly complete hospital database capture of hip fractures, they can help monitor disease burden and healthcare response after implementing clinical and organizational improvements in the care for the elderly^11,12^.

Several studies have shown a marked variation worldwide in hip fracture incidence, with the highest rates reported in Sweden and other Scandinavian countries^13^. A recent analysis based on national databases^14^ forecasted a steep increase in fragility fracture burden in Scandinavia and most other European countries, with 30% more fractures in 2034 than in 2019. Older projections estimated a 2–3 times higher number of hip fractures in 2050 than in 1990^15^ and 2002^16^. However, the validity of projections regarding hip fracture incidence depends on the accuracy of data inputs, which is often complicated by the original design of national databases that record healthcare encounters without distinguishing between multiple encounters for a single injury.

To address this issue, we estimated the number of hip fractures in Sweden over the past two decades, calculated time trends, estimated 10-year risks of a new hip fracture, and made annual projections through 2050. This study demonstrates the potential problems with using national databases for incidence studies and highlights the importance of accurate data inputs in estimating hip fracture burden.

## Methods

### Hip fractures captured by a national register and development of our database

We estimated hip fracture rates and numbers of hip fractures from 1998 to 2019 using the Swedish National Patient Register (NPR), maintained by the Swedish National Board of Health and Welfare^1^. The register has covered all inpatient care in Sweden since 1987. Swedish law mandates all private and publicly funded physicians and hospitals to deliver complete data to the NPR. The estimated positive predictive value for a first diagnosis of hip fracture in the NPR verified by hospital records ranges from 95 to 99%, depending on the study^1,17,18^. The negative predictive value has been estimated to be 100%^1,17,18^. In addition to the unique personal identification number (PIN) that is given to all Swedish citizens^19^, the register includes information on the primary diagnosis, secondary diagnoses, comorbidities, type of injury, surgical procedures, type of department, and type of admission (elective or urgent). No variable indicates whether the admission is the first (“index”) admission for an injury, transfer, or readmission for continuing care. Since 1997, the diagnoses have been coded according to the International Classification of Diseases version 10 (ICD-10; ICD-9 was previously used).

We used a database from the NPR covering all hospital admissions for injuries from 1998 to 2019. We additionally identified all previous injury admissions in 1987–1997 for these patients using the PIN for linkage. The NPR captures all publicly funded in-patient (and specialist outpatient) care in Sweden, including geriatric and rehabilitation ward admissions.

We identified possible hip fracture patients from 1997 to 2019 using ICD-10 codes S720, S721, or S722^7^. In addition, national annual age- and sex-specific population numbers from the study period and the expected number of Swedish inhabitants through 2050 were retrieved from Statistics Sweden.

We used these data to conduct two sets of analyses, differing only in the definition of incident hip fractures. Our first set of analysis naïvely accepted as an incident fracture any hospitalization with one of the hip fracture codes enumerated above. In a parallel analysis, incident hip fracture events were identified using a previously developed and individual hospital-record-validated prediction model^7^ identifying an incident injury. The model was developed for any injury admission but was separately validated for hip fractures. The probability for the admission being a readmission was calculated based on patient age, sex, type of admission (acute or elective), time interval from the previous injury admission, similar main diagnosis as previous admission (the first 4 positions in the ICD-10 main diagnoses being identical), and admission to a department for rehabilitation. The probability cut-off of 0.4932 to identify incident admissions was optimal from the receiver operating characteristic curve in the validation study. This model omitted readmissions after a previous “index” injury, although the admission had been coded as an acute hip fracture^7^.

Our national database from the NPR, combined with our algorithm, shows that only 1.6% of hip fractures in 1998–2019 occurred before age 50. Because fractures coded as hip fractures in younger generations have a different etiology than those at older ages^20,21^, we restricted our analyses to women and men > 50 years.

### Description of the algorithm and code used to identify incident hip fractures

Derivation and validation of the prediction model variables and their multiplicative interactions used logistic regression modeling to develop a prediction model that could separate incident hospital admissions from readmissions. The algorithm is based on age, sex, diagnoses, time intervals between admissions, type of admission, type of injury, and type of hospital department^7^—the derived SAS code appears in Supplementary file 1. The model was developed and validated using data representative of the Swedish national population^7^ after identifying correctly classified incident hip fractures based on individual hospital records review^7^. One key point is that cases in which a patient’s discharge was followed by a new admission dated within one day were classified as transfers between departments or hospitals and counted as one fracture episode. By applying the model on a separate dataset by data split^7^, we could evaluate and quantify the model's performance, enabling sensitivity analysis to illustrate the effect of misclassification. Based on the separate validation data set, the model has 96% sensitivity for incident hip fracture in patients hospitalized due to injury and 96% specificity^7^.

In a complementary analysis the hip fracture admissions predicted as incident were also checked for the presence of surgical procedure codes for hip fracture surgery (Swedish national procedure codes for external traction [TNX40], reposition, fixation or osteosynthesis [NFJ09–NFJ99], or primary arthroplasty [NFB09–NFB99]). Of note, some Swedish regions have in periods not reported all hip fracture-related surgical procedure codes to the NPR.

### Statistical analysis

Our study was conducted through four interconnected statistical analyses. Our initial analysis compared the annual number of algorithm-estimated incident hip fracture admissions from 1998 to 2019 with estimates generated by the naïve approach (including hospital transfers, readmissions, and complications). In sensitivity analyses, we examined combinations of primary diagnosis, secondary diagnoses, and hospitalization sequences in each chain (within ≤ 1 day) of hospitalizations to identify incident fractures.

In addition, our results were compared to the official annual number of hip fractures presented by the Swedish National Board of Health and Welfare (https://www.socialstyrelsen.se/en). Their SAS code for hip fracture ascertainment can be found in Supplementary File 2. The underlying assumption for the official statistics is that an individual recorded with one or more care events, including an ICD code for hip fracture in a given year, is classified as having had one incident hip fracture in that year. Each care event that includes an ICD code for hip fracture is counted as an incident case, even though the patient could have been treated for a complication after a previous year’s hip fracture or had a hospital transfer near the turn of the year. Although the event contains an ICD complication code and a code for a hip fracture, this event is counted as a new hip fracture. On the other hand, patients who sustained two incident hip fractures during the same year are classified with only one hip fracture; the authority officially presents the number of hip fractures annually using 3-year moving averages.

Second, we evaluated the cumulative incidence of a new hip fracture after an index hip fracture event for the whole study period and within two time windows: 1998–2008 and 2009–2019, the latter a period with more substantial national secondary prevention initiatives^22^. We performed this analysis using three approaches: (1) the algorithm identification of each new hip fracture; (2) the naïve approach for a new hip fracture case identification; and (3) the naïve approach but with exclusions of a new admission dated within one day after the incident hip fracture event. Because death rates are high after a hip fracture event, which affects the likelihood of a new hip fracture^23^, we used individual linkage to the Swedish Cause of Death Register^24^ to enable consideration of death as a competing event.

Third, we assessed age- and sex-standardized trends in the numbers of hip fractures during the period 1998–2019. For each calendar year, we computed age- and sex-specific hip fracture rates, using age strata 50–65, 65–69, 70–74, 75–79, 80–84, 85–89, 90–94, ≥ 95 years, based on algorithm-estimated number of fractures and annual age-and sex-specific population data from Statistics Sweden. These incidence rates were used to calculate age-standardized annual numbers and relative changes in hip fractures using direct standardization with the 2019 population as a reference. For the estimated proportional change from 1998 to 2019, 95% confidence intervals were derived with the percentile method from bootstrapping with 1000 replications.

Fourth, projections of hip fracture numbers from 2020 to 2050 were made under three assumptions: (1) a continued annual decline in hip fracture rates from 2020 through 2050 corresponding to the average annual percent change in 1998 to 2019; (2) rates constant from 2019 and onwards; and (3) a combination of scenario 1 and 2 with a continued downward trend in hip fracture rates from 2020 through 2029 followed by no further change. The rates were calculated by 5-year age bands and sex and applied to projected population numbers from Statistics Sweden. We displayed the population statistics in absolute numbers as supplementary information and relative changes by sex and age (50–74 and ≥ 75 years) from 1998 to 2050.

SAS version 9.4 was used for initial data management, and R version 4.3.0 was used to calculate incidence estimates. Cumulative incidence was plotted using subdistribution functions in competing risks^25^, as implemented in the R package cmprsk (version 2.2-11).

### Ethical approval

The study was approved by the Swedish Ethical Review Authority (No 2020-03336).

## Results

In the analysis that accepted any diagnosis of hip fracture, there were 539,947 separate hip fracture admissions from 1998 to 2019 in individuals > 50 years, 366,787 in women, and 173,160 in men. In contrast, the numbers from our algorithm were considerably lower: 335,399 hip fractures (234,475 for women and 100,924 for men). The naïve analysis overestimated the number of hip fractures by 56% in women and 72% in men. Official statistics from the Swedish National Board of Health and Welfare claim 367,697 hip fractures from the 22-year study period, 112,824 in men and 254,830 in women, i.e., 12 and 9% higher than our algorithm-derived numbers.

**Figure 1:**
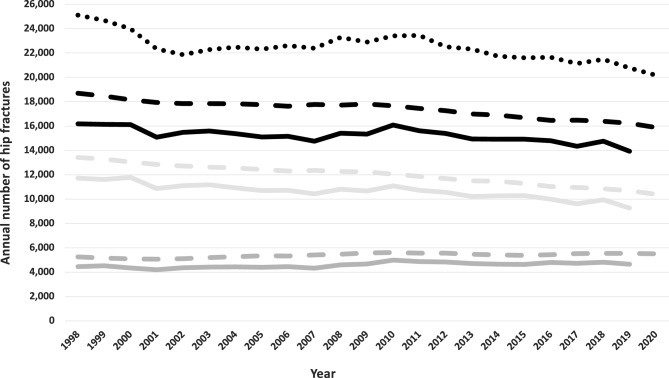
The algorithm-based actual number of hip fractures in Sweden from 1998 through 2019 in women and men > 50 years, overall (solid black line) and by sex (light grey = women dark grey = men). The dashed lines refer to the yearly number of hip fractures, according to official statistics from the Swedish National Board of Health and Welfare. The black dotted line illustrates the naïve analysis, with the overall number of hip fracture diagnoses each year recorded in the national Swedish Patient Register.

Our algorithm identified 16,180 hip fracture cases over age 50 in Sweden in 1998 (Fig. 1), declining to 13,929 in 2019, a 14% decrease. This reduction was due entirely to trends in women, with a 21% reduction in hip fractures versus a slight increase in men. The *relative* decline in hip fractures between 2019 and 1998 is comparable to those estimated using the naïve hip fracture identification approach (17% reduction) or official statistics from the Swedish National Board of Health and Welfare (13% reduction).

**Figure 2:**
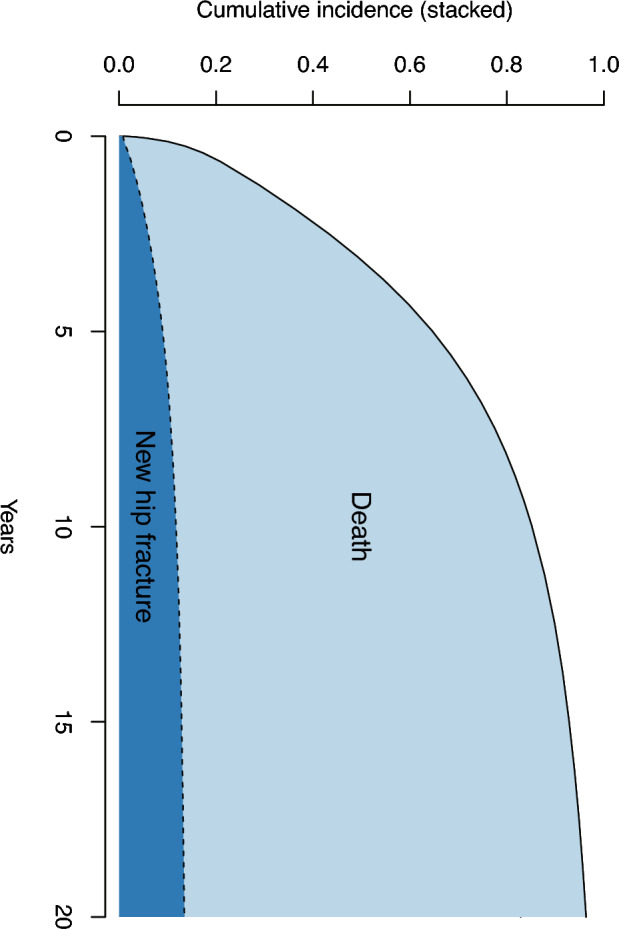
Our algorithm-based cumulative incidence of a second hip fracture and death after the index hip fracture event.

We additionally evaluated the risks of a second hip fracture. Within the first decade after an index hip fracture event, about 80% of hip fracture patients had died, and 11% had suffered a new hip fracture (Fig. 2). This pattern was evident (Supplementary Fig. 1) from the first half (1998–2008) and the second half of the observational period (2009–2019). In contrast, using a naïve approach, the proportion of patients with a second hip fracture from the first decade after the index hip fracture increased to 35% (Supplementary Fig. 2), an apparent tripling compared to the results based on our algorithm. If we used the naïve approach but ruled out adjacent admissions as unique cases, the 10-year risk of a new hip fracture was estimated to be 18% (Supplementary Fig. 3).

To better interpret the alterations in the number of hip fractures, we need to consider changes in age-specific incidence using the population's age-, sex- and year-specific structure. Between 1998 and 2019, the life expectancy of a 65-year-old Swede increased by 2.0 years in women and 3.2 years in men. During the same period, we observed a continuously increasing mean age at hip fracture, reaching 83.5 years in women and 80.7 years in men (Supplementary Fig. 4). In our Swedish setting, approximately 80% of all hip fractures occurred after 75 years (82% in women and 76% in men). Compared to 1998, the number of individuals 75 + years in 2019 had increased by 24%, with a moderate 13% increase in women compared to 41% in men in this age category.

**Figure 3:**
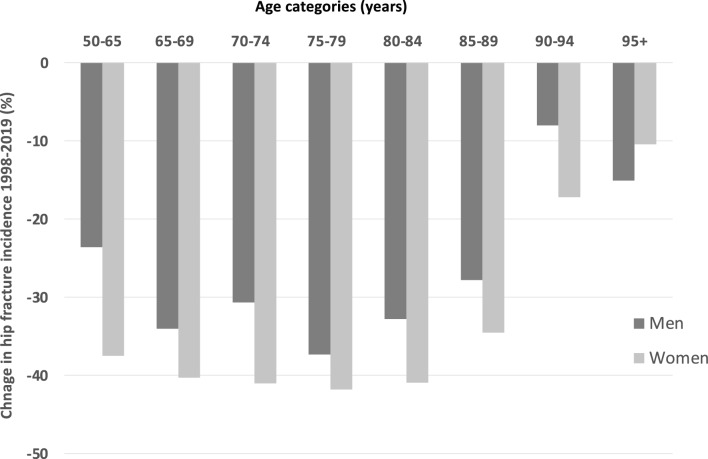
Percent change in annual hip fracture incidence rates from 1998 to 2019 in women and men > 50 years by sex (women = light grey, men = dark grey) and age.

**Figure 4:**
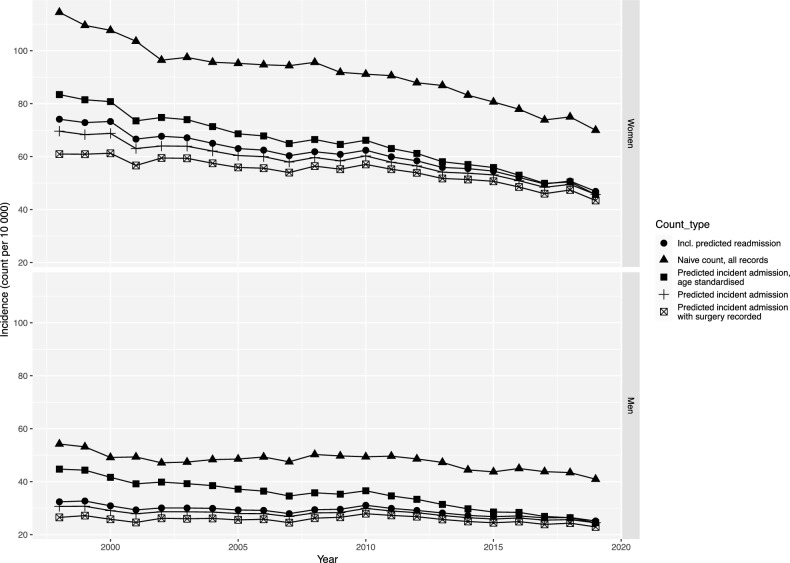
Hip fracture incidence from 1998 through 2019: (1) estimated by a prediction algorithm for incident fracture events and observed population structure (cross); (2) estimated by a naïve incidence calculation of all admissions and observed population structure (filled triangle); (3) estimated using prediction algorithm rates with age-standardization (to the population structure as of 2019) predicted incident admission (filled square); (4) estimated using a prediction algorithm for incident fracture events and in addition, predicted readmissions with the observed population structure (filled circle); and (5) estimated by a prediction algorithm for incident fracture events combined with a surgical procedure code with the observed population structure (checked box).

**Figure 5:**
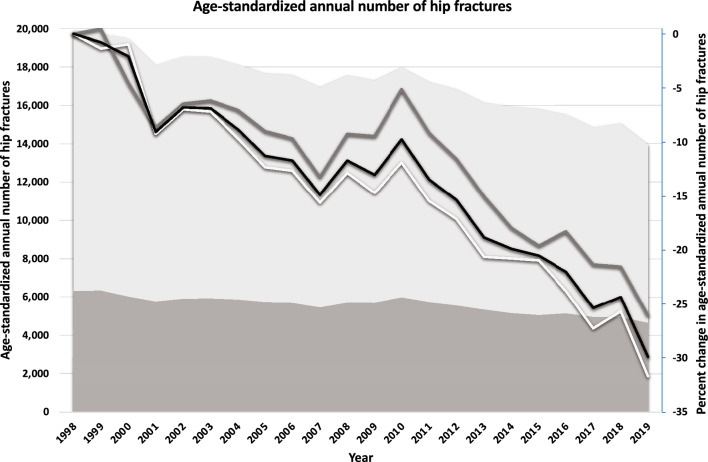
Age-standardized annual number of hip fractures (primary vertical axis) from 1998 through 2019 with direct standardization to the population of 2019. The light gray area represents women, and the dark grey area represents men. The percentage change (secondary vertical axis) in the age-standardized number of hip fractures from 1998 (reference) through 2019 is displayed as solid lines. The overall results are shown as a solid black line, a solid light gray line representing women, and a dark gray line representing men.

The aging Swedish population would increase the number of hip fractures. Still, the decreases in age-specific fracture rates during 1998–2019 (Fig. 3) and overall incidences during 1998–2019 (actual, age-standardized, actual combined with a surgical procedure code, actual incidences including estimated readmissions, and naïve incidences) in Fig. 4) more than compensated for this, as shown by the dramatic decline in the age-standardized number of hip fractures over time (Fig. 5). With the population in 2019 as a reference, there was a 29.2% (95% CI 28.1–30.2) reduction in the age-standardized number of hip fractures from 1998 through 2019 in women and a corresponding reduction of 29.3% (95% CI 27.5–30.7) in men. This corresponds to about 6000 fewer hip fractures in 2019 compared to 1998 if the age-sex population structure had been identical.

**Figure 6:**
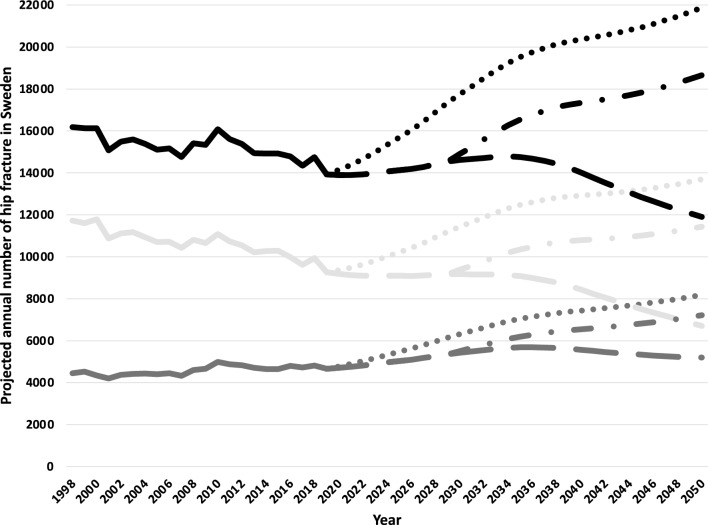
The actual number of hip fractures in Sweden from 1998 through 2019 in women and men > 50 years, displayed overall (black line) and by sex (light grey = women dark grey = men). Shown are also projections through 2050 assuming a continued decline in incidence (dashed lines), a continued decline in incidence through 2029, and after that, no further reduction in the incidence (combined dashed and dotted lines) or no further decrease in the incidence (dotted lines).

There is an expected continued demographic trend towards an older population in Sweden. From 2020 to 2050, the number of Swedish citizens > 75 years is projected to increase by 45%. The relative increase will be more pronounced in men, although the absolute number of older women will continue to be higher (Supplementary Fig. 5). Assuming the average proportional decline in hip fracture incidence from 1998 to 2019 continues from 2020 to 2050, and using the population changes projected by Statistics Sweden, we estimate the absolute number of hip fractures in 2050 will fall to less than 12,000 per year (Fig. 6, dashed lines). The total number of hip fractures in men will approach those in women. By contrast, if the decline in hip fracture rates does not continue, we can naturally expect an increase in the total number of hip fracture events because of the growing number of older individuals, bringing the total to about 22,000 in 2050 (Fig. 6, dotted lines). In the third intermediate scenario, there would be approximately 18,500 hip fractures annually in 2050 (Fig. 6, dash-dot lines). In a 15-year projection from 2019, the number of hip fractures expected in 2034 would be 14,380 under scenario 1, 19,200 under scenario 2, and 16,300 under scenario 3.

### Sensitivity analyses

In a sensitivity analysis (Supplementary Fig. 6), the algorithm estimates of the number of hip fractures were similar irrespective of whether we considered only the primary diagnosis at the first hospitalization in each unique chain of hospitalizations, the primary diagnosis at any hospitalization in each chain, or if we used primary or secondary diagnoses (but not both) in each chain of hospitalizations. This congruence in estimates was observed in women and men. Regardless of the method, there was a steady decline in incidence in both sexes from 1998 to 2019 (Supplementary Fig. 6). There was regional variability but a consistent decline in regional incidence overall (Supplementary Fig. 7). Using the prediction model to identify incident hip fractures, 0.46% (953/207,982) of female subjects and 0.38% (353/92,036) of male subjects were registered with > 2 hip fractures. We also performed a complementary analysis looking at the proportion of predicted incident hip fracture admissions that were and were not associated with a surgical procedure code for hip fracture surgery. In women, the proportion of predicted incident admissions not having an associated surgical procedure code decreased over time from 14.2% in 1998 to 5.3% in 2019. In men there was a similar decrease from 15.6% in 1998 to 6.9% in 2019.

## Discussion

This study demonstrates that declining incidence rates of hip fractures have more than offset the aging Swedish population, resulting in a marked decrease in the number of hip fractures in Sweden since 1998. With age- and sex-standardized calculations, the reduction is approximately 30%. We also show that incident hip fractures and recurrent hip fracture events in Sweden can be substantially over-reported without careful use of population fracture data. This is the first investigation to use an individual hospital-record-validated algorithm to identify incident hip fracture cases to describe the national burden of hip fracture in Sweden.

Our findings corroborate previous reports of a continuous decline in age-standardized actual hip fracture incidence in Swedish women and men^26^. These authors used individually hospital-record validated data from northern Sweden, though only from 1993 to 2005^26^. Norwegian investigators have similarly observed a decline in age-standardized hip fracture incidence from 1999 to 2013 using a careful methodology^27^. Other Nordic studies using less formal methods have also seen downward trends in recent years^28–32^. Three of the studies used only first hip fracture events to calculate incidence trends^3–5^. Rosengren et al.^6^ combined an annual ICD code for proximal femoral fracture and a relevant surgical procedural code to identify an incident hip fracture. Thus, the patient could not have had two hip fractures in the same calendar year. With this approach, there is also the problem with underreporting procedure codes in the Swedish National Patient Register, leading to neglecting actual hip fracture events. In addition, inpatient-treated complications after a hip fracture are not uncommon, and problematically, many of the complications are coded in combination with a hip fracture code. Rosengren’s approach will accordingly misclassify a miscoded complication as a new hip fracture if the secondary surgery is performed the following year. Meyer et al. used first hip fractures, and in addition, hip fracture codes more than two weeks after a previous hospital admission for hip fracture were considered recurrent hip fracture events^7^. Furthermore, in several of the studies^3,5–7^, it is unclear, from the Methods descriptions, whether only the main diagnosis in the patient register was used or also secondary diagnoses codes. We observed a downward trend in hip fracture events irrespective of how fractures were ascertained. We found it with a naïve analysis, official Swedish statistics, and our more accurate algorithm-based approach. The same downward trend has been seen in other European settings^33–36^, while trends have plateaued in North America^37^. In contrast, hip fracture rates are increasing in Asia^38–41^. The present study also confirmed previous reports with careful identification of incident hip fracture events^42–44^ that only about 10% of hip fracture patients have a second hip fracture within ten years, and high mortality rates may largely explain this moderate risk following a hip fracture^23,45^.

Multiple potential causes may explain the downward trend in Swedish hip fracture rates, including a more healthy lifestyle with the avoidance of sedentary behavior^46,47^, a reduction in cigarette smoking (down to a 6% prevalence in recent years^48^), and healthier dietary habits^49–53^. Moreover, there have been increases in body weight^54–56^ and more common use of hip arthroplasties, precluding hip fracture injuries^57^. Positive, healthy lifestyle trends are likely to continue in Nordic countries, with strong influences from different authorities advocating and instructing the inhabitants on how to live a healthy life—the Nordic nutritional recommendations are just one example^58^.

Other factors may have played a role in lowering hip fracture rates. Because of a possible direct causal link between cardiovascular diseases and the risk of hip fracture^59^, the 40% reduction in both ischemic heart disease^60^ and stroke^61^ incidence in Sweden during the past two decades may also have contributed to the reduced hip fracture burden. Increases in cataract surgery in Sweden^62^ could reduce the risk of falls and subsequent hip fractures^63^.

Some may argue that preventing fractures by bone-specific drugs and Fracture Liaison Services^22,64,65^ should have affected the number of hip fractures. However, from 2005 to 2020, among Swedish women older than 55, the prevalence of bisphosphonate users decreased from 4.9 to 3.6%, while in men, the corresponding proportion was approximately 1%. This pattern is unlikely to substantially affect hip fracture rates at a population level^66^. Moreover, we found a similar age-adjusted decrease in hip fracture in men and women, even though women have a higher frequency of bone-specific drug treatment. Over the past decade, denosumab has been introduced as an additional bone-specific drug. In 2019, approximately 0.7% of all women and 0.1% of all men > 55 years were prescribed the medication. Finally, our results show a decline in age-adjusted hip fracture burden before initiating the Fracture Liaison Services in 2012. The proportion of patients with a new hip fracture remained stable during our study period.

A non-clinical factor contributing to the declining hip fracture rates might be that in Sweden, the immigrant population has grown in recent decades [Statistics Sweden; https://www.scb.se/], and many have moved from settings with a lower risk of fractures than in Scandinavia. From 2000 through 2019, the proportion of foreign-born women and men > 75 years in Sweden increased from 7 to 12%. The immigration impact on hip fracture rates is, therefore, likely modest.

Compared with our individual hospital-record validated algorithm to identify the number of incident hip fracture cases, the burden of hip fractures in Sweden seems to have been overestimated in several previous reports^13–15^. These estimates, together with the fact that lowered time trends in hip fracture rates should have been taken into account, have also led to inflated projections of future risk. Our 2034 and 2050 projections sharply contrast previous estimates^10,16,67,68^. We estimate that the number of hip fractures in 2050 will be *lower* despite a growing number of older adults if the incidence trends we observed in the past two decades continue into the mid-century.

Our results are of importance to clinicians. At first glance, the presentation of naïve fracture number estimates, in addition to the accurately calculated numbers, seems unnecessary. However, such high incidence estimates that apparently do not fully restrict the count to incident fracture events^14^ are used in the widely adopted fracture risk calculator, FRAX^14^, likely overestimating the FRAX 10-year risk of hip fracture for individual patients. The reported Swedish incidence of hip fracture in 2020 for the population aged 50–89 years of age^14^ corresponds to 20,650 Swedish hip fractures, approximately 50% higher than our algorithm-based actual estimate—even though we also included hip fractures in the oldest old of 90+ years, a group excluded from the figures in Kanis’ publication^14^. Based on our data, these cases among those aged 90 years or older contribute 27% of all incident hip fractures in women and 19% in men. As a comparison, our naïve estimate for 2020 is 20,176 Swedish hip fractures, including women and men aged 50 years or older, while the actual numbers, according to our algorithm-based calculation, are > 6000 fewer hip fracture cases.

Moreover, the ratio between Swedish hip fracture rates and those of other types of fragility fractures is used in the FRAX calculations for countries without complete register information^69,70^. The overestimation of Swedish incident hip fracture numbers may, therefore, also be a concern for other countries.

Similarly, FRAX seems to provide high 10-year risks for a second hip fracture. We used the online FRAX calculator and computed the 10-year FRAX probability of a hip fracture for Swedish women aged 80–85 years of average height and weight and with a previous fracture of *any type* but without any other risk factors. The estimate is 20–22%, considerably higher than those we and other researchers estimated using population-based databases^42–44^. In addition, according to the FRAX calculator, the 10-year risk of hip fracture is greater than 50% in that clinical setting if there is additionally a parental history of hip fracture, a marker of risk inferior to the patient’s history of a previous hip fracture. The present study confirmed^42–44^ that only about 10% of hip fracture patients have a second hip fracture within 10 years, and high mortality rates may largely explain this moderate risk following a hip fracture^23,45^.

The validity of our calculated number of hip fractures has been confirmed by manual case identification using individual patient hospital records in localized regions in Sweden and with external validity to national data^26,71^. However, whether the potential for substantial overestimation of fracture risk from national databases can be generalized to settings outside Sweden requires detailed validation of the case identification algorithms (if any) used. In addition to our algorithm for identifying incident injury cases^7^, our study is strengthened by the national population-based design and individual-based register data linked using the Swedish PIN. The trend of lower hip fracture rates is gratifying, but our study design cannot decipher which individual lifestyle factors have most tangibly driven this development.

The methodological issues we faced in ascertaining hip fracture cases in a large population are relevant for other disease endpoints for which it is essential to identify incident cases of disease, e.g., myocardial infarction^4,72^, stroke^5,73^, colorectal cancer^74^, pneumonia^6^, and different types of injuries^7^. Methodology for accurate estimates requires an ascertainment algorithm validated against individual medical records. Using raw register data may result in serious bias. Many difficulties can be overcome in databases with unique PINs that enable reliable record linkage. This linkage allows for multiple medical encounters to be used to identify incident disease events, avoiding double counting. The issues we describe are even more critical when using administrative databases, such as those from Medicare in the US, without direct individual identifiers^75,76^.

The potential limitations of the methods used in this study should be recognized. The extent of coding errors in the Patient Register is diagnosis-dependent, and the potential impact on hip fracture diagnoses recorded over the long study period is unknown^77^. The prediction model used to identify incident injuries will inevitably result in some residual misclassification. Less than 0.5% of subjects were registered with > 2 hip fractures, a potential limitation but also a clinical possibility since the patient could first have had a cervical hip fracture and later a sub- or pertrochanteric hip fracture. Similarly, a fraction of predicted admissions did not have an associated procedure code for hip fracture surgery reported, which is indicative of misclassification in the NPR. Our algorithm did not involve procedure codes, and given the degree of missingness, we do not recommend the approach to identify an incident Swedish hip fracture by combining a hip fracture code and a surgical procedure code. We have tried to address the remaining misclassification by presenting alternative methods to estimate the number of hip fractures, thereby contextualizing this concern. The uncertainty of projections is inevitable, and they should be interpreted cautiously.

Our findings show a substantial recent over-reporting of hip fractures in Sweden^14^. In addition, over the past two decades, there has been a continued decline in the overall and age-standardized number of hip fractures in Sweden. Accordingly, Sweden's future hip fracture burden will almost certainly be lower than previously calculated^14–16^. This lower fracture incidence will affect health economic analyses and fracture prediction tools in Sweden and other countries.

### Supplementary Information

Supplementary Information.

## Data Availability

The SAS code for calculating incident injuries, including hip fractures, can be found in Supplementary File 1. The datasets analyzed during the current study are not publicly available because the data contain sensitive personal information, and such data cannot be publicly available according to the EU’s General Data Protection Regulation. Data are, however, available upon ethical approval and application to the Swedish National Board of Health and Welfare.
