# Supplementary material for: Declining hip fracture burden in Sweden 1998–2019 and consequences for projections through 2050
Source: Sci Rep. 2024 Jan 6;14:706. doi: 10.1038/s41598-024-51363-6 (PMC10771431; doi:10.1038/s41598-024-51363-6)
Supplement: Supplementary file 1 — Supplementary Information. [file 41598_2024_51363_MOESM1_ESM.pdf]

Supplementary information

Declining hip fracture burden in Sweden 1998-2019 and consequences for projections through 2050

Karl Michaëlsson et al

**Content**	**Page**
Supplementary Figure 1	2
Supplementary Figure 2	3
Supplementary Figure 3	4
Supplementary Figure 4	5
Supplementary Figure 5	6
Supplementary Figure 6	7
Supplementary Figure 7	8
Supplementary File 1	9
Supplementary File 2	63

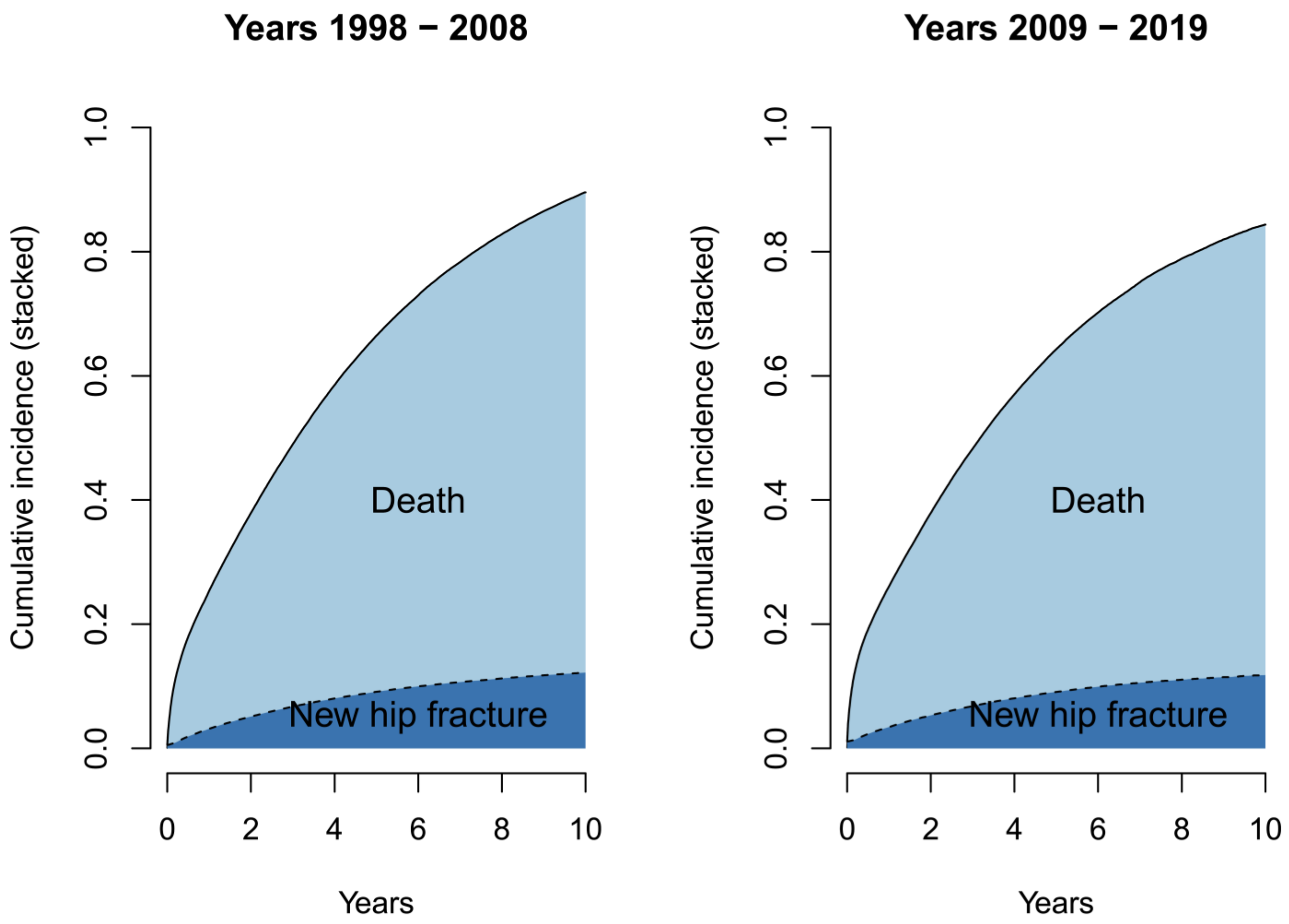

Supplementary Figure 1. Cumulative incidence of a second hip fracture and death in 1998-2008 and 2009-2019.

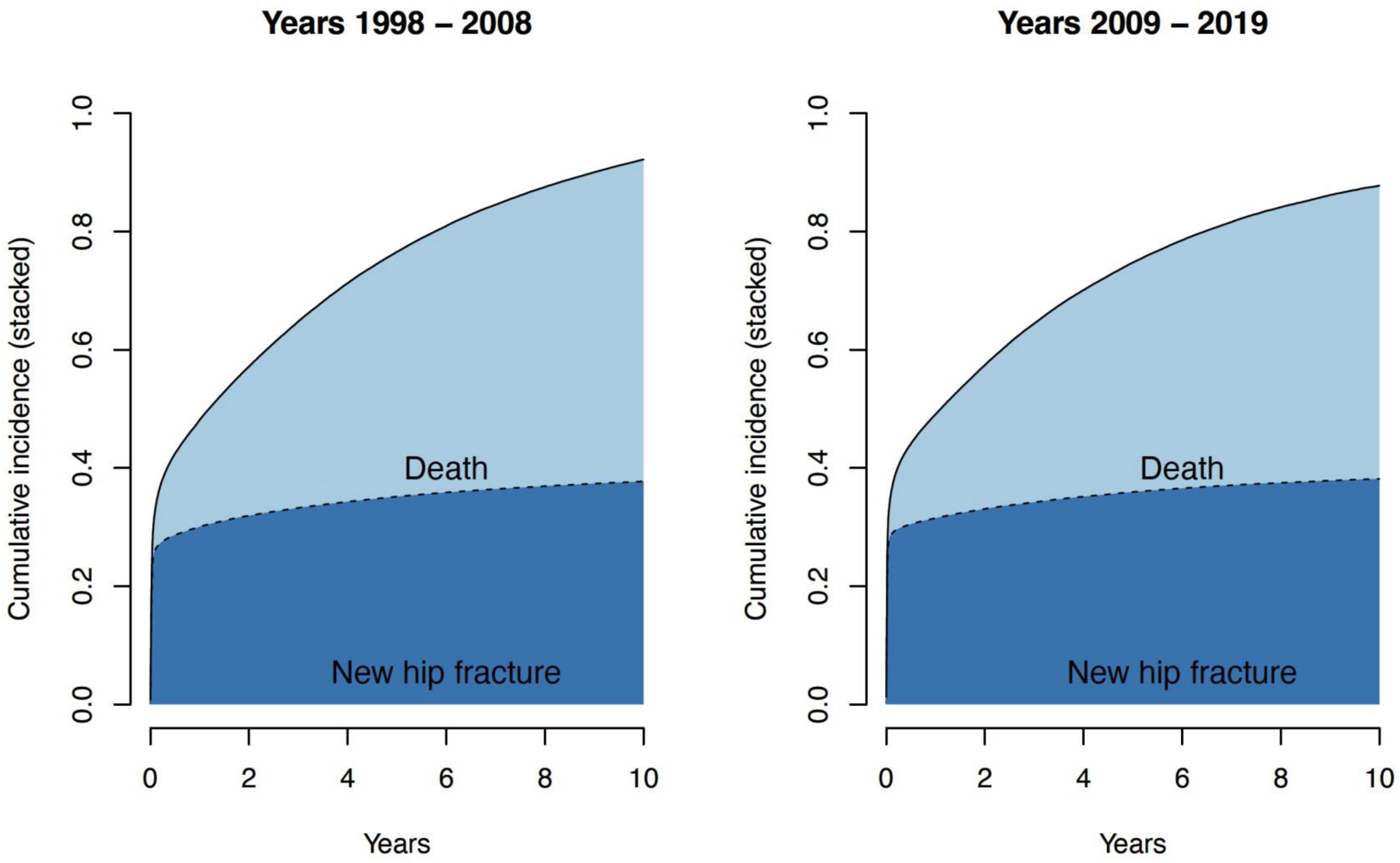

Supplementary Figure 2. Cumulative incidence of a second hip fracture and death in 1998-2008 and 2009-2019 using the naïve approach to identify a new hip fracture.

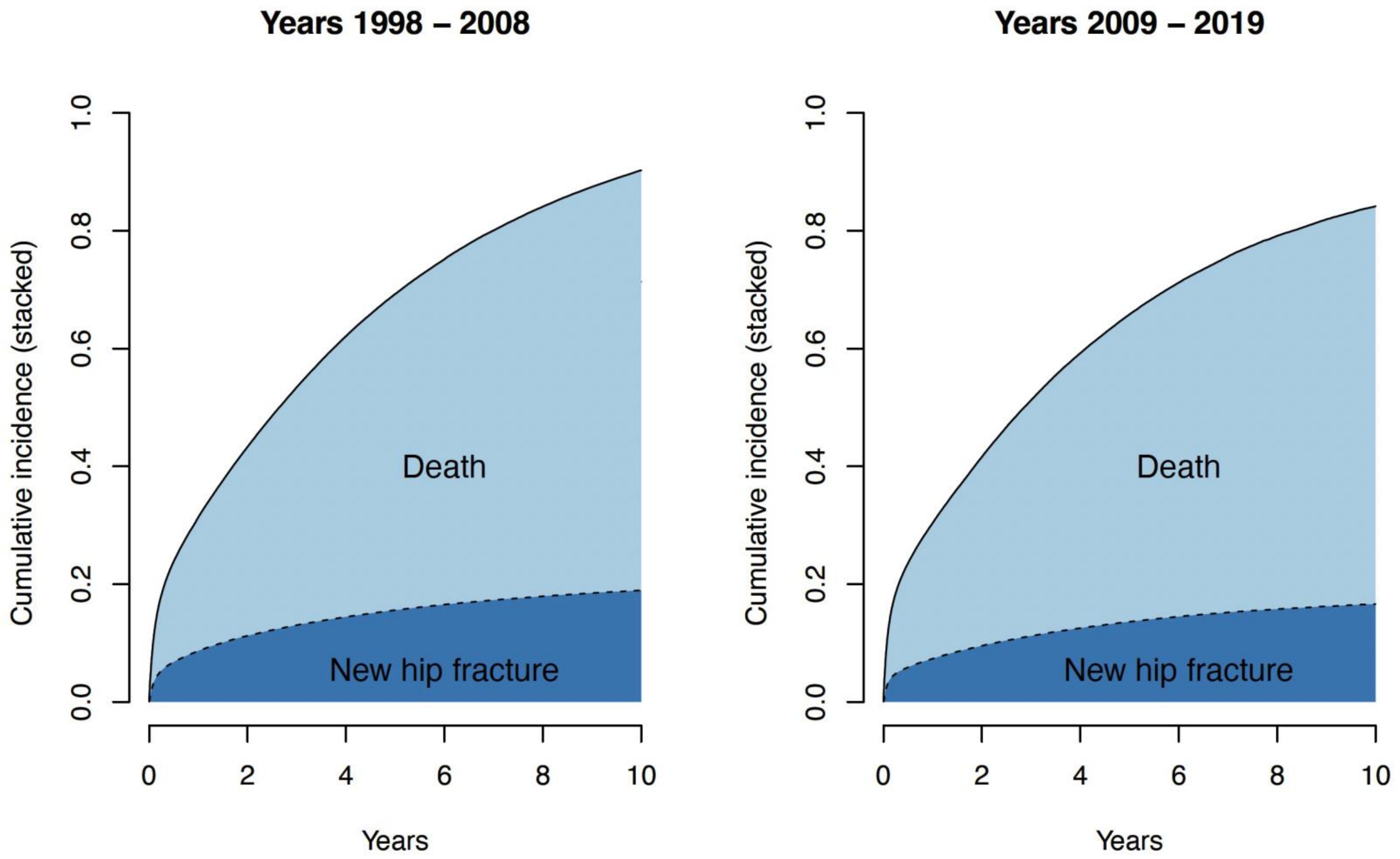

Supplementary Figure 3. Cumulative incidence of a second hip fracture and death in 1998-2008 and 2009-2019 using the naïve approach but excluding adjacent admissions as unique new cases.

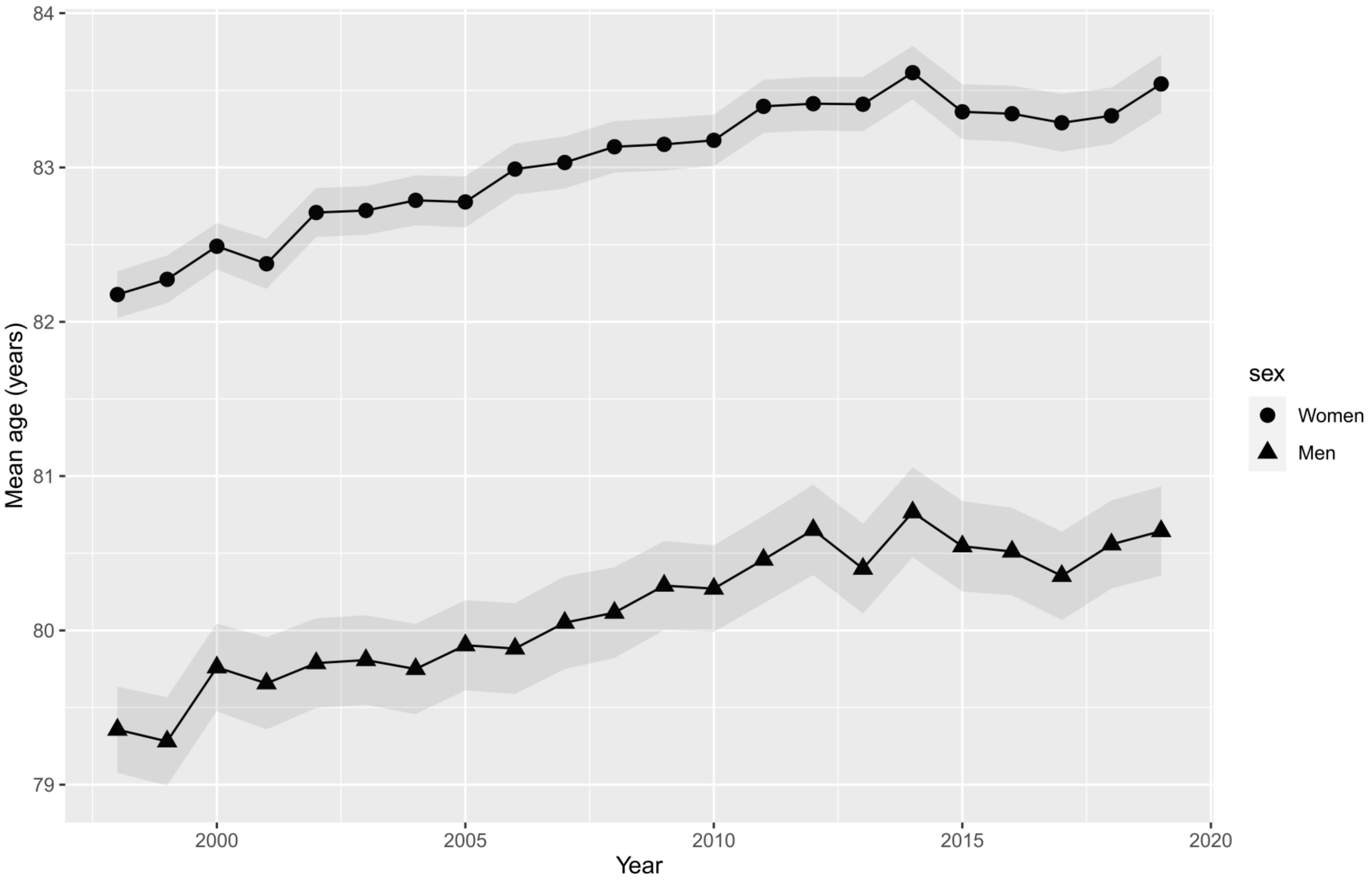

Supplementary Figure 4. The mean age of predicted incident hip fractures from 1998 through 2019. Gray shaded area indicates a 95% confidence interval.

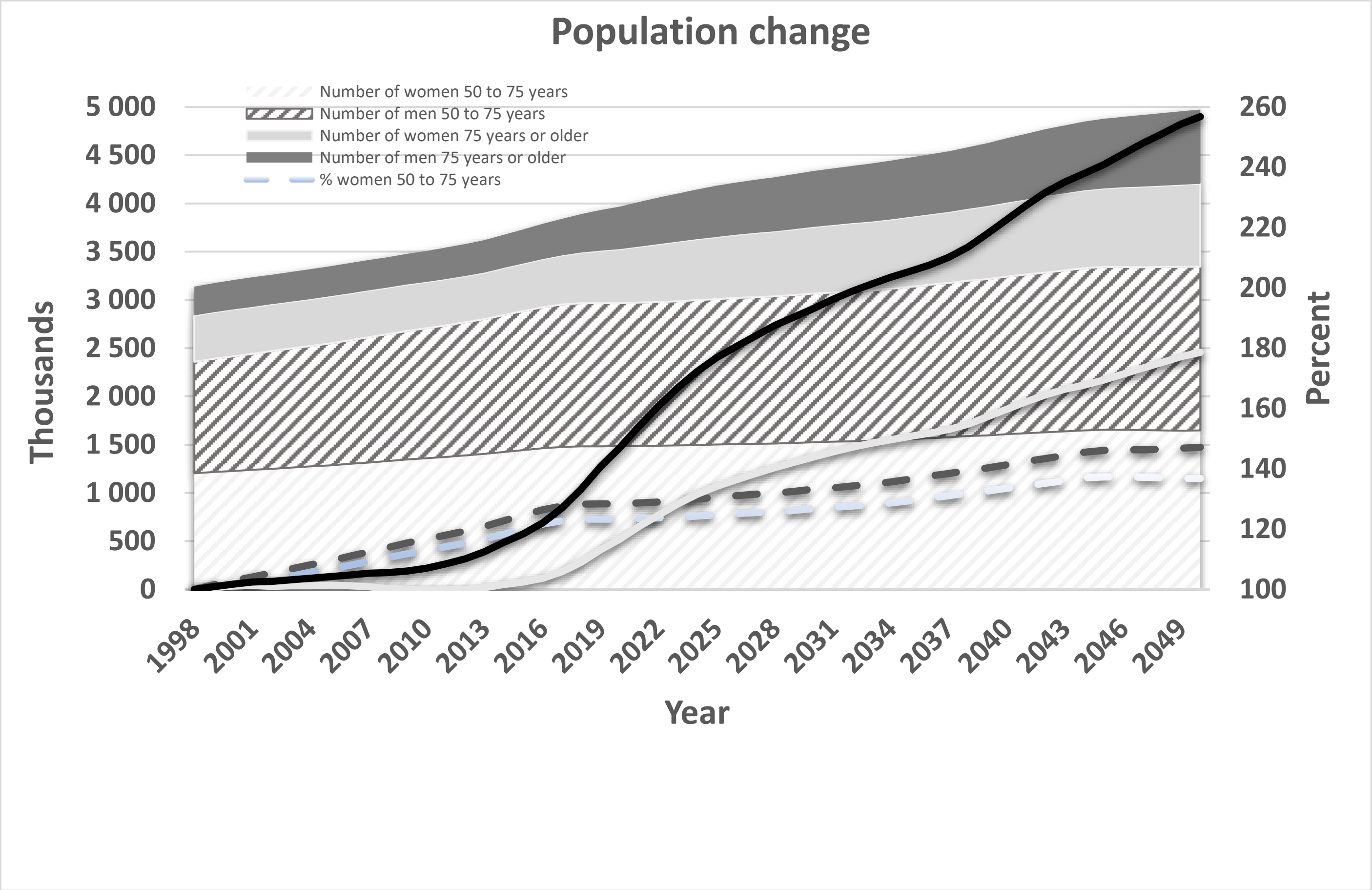

Supplementary Figure 5. Swedish population changes in people ≥50 years from 1998 through 2050. The cross-hatched areas represent ages 50-74 years, with the light grey area corresponding to the number of women and the dark grey area to the number of men (primary vertical axis). The dashed lines correspond to the relative change in the population, expressed as percentages relative to 1998 (secondary vertical axis). The solid areas and lines represent women and men aged ≥75 years.

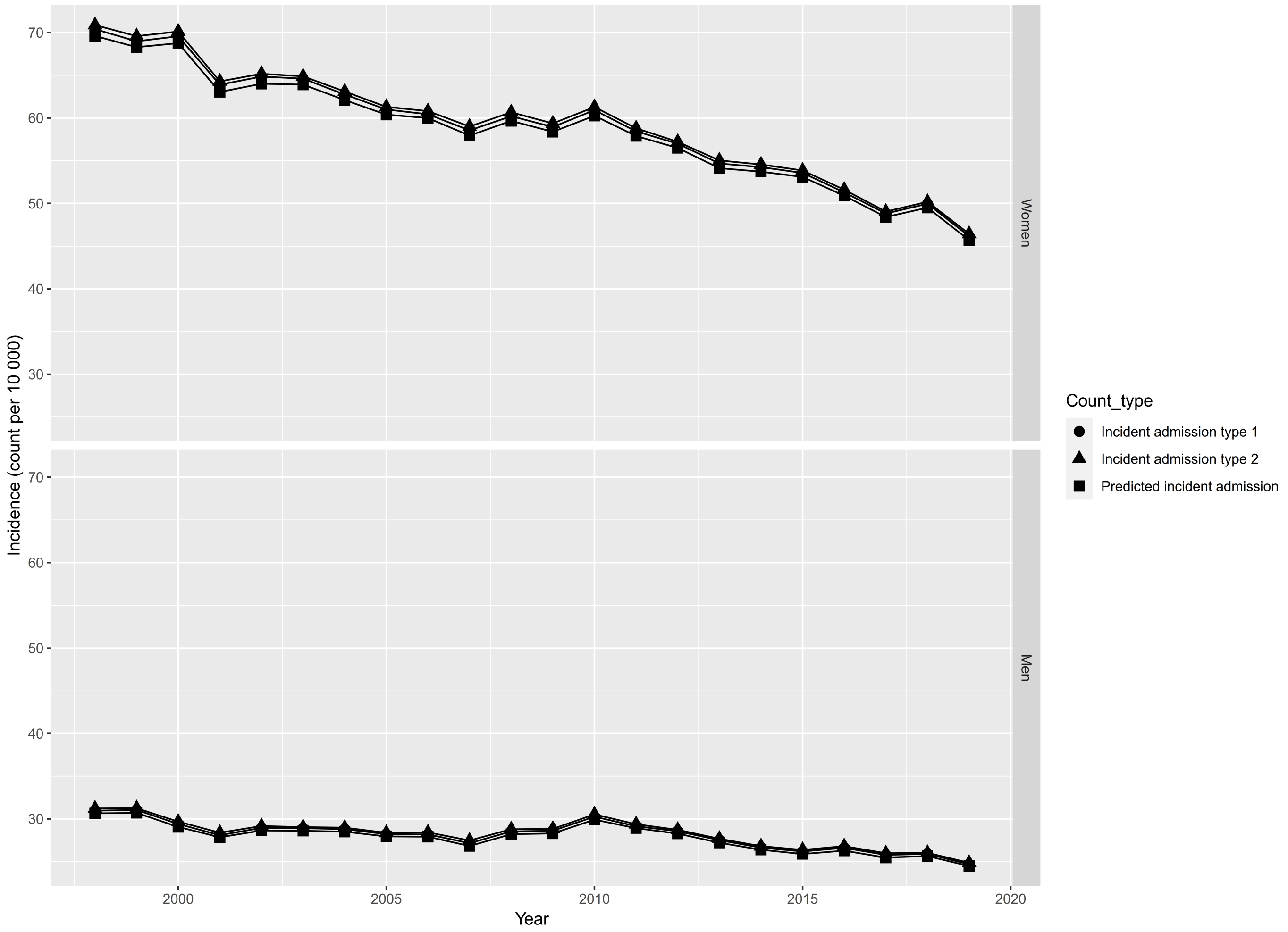

Supplementary Figure 6. Incidence of hip fracture from 1998 through 2019: 1) estimated using a prediction algorithm and primary diagnosis from the first hospitalization in a chain of hospitalizations (square); 2) estimated using a prediction algorithm and primary diagnosis at any hospitalization in a chain of hospitalizations (circle); and 3) estimated using a prediction algorithm and primary or secondary diagnoses at any hospitalization in a chain of hospitalizations (triangle). The background population was restricted to 50 years of age or older.

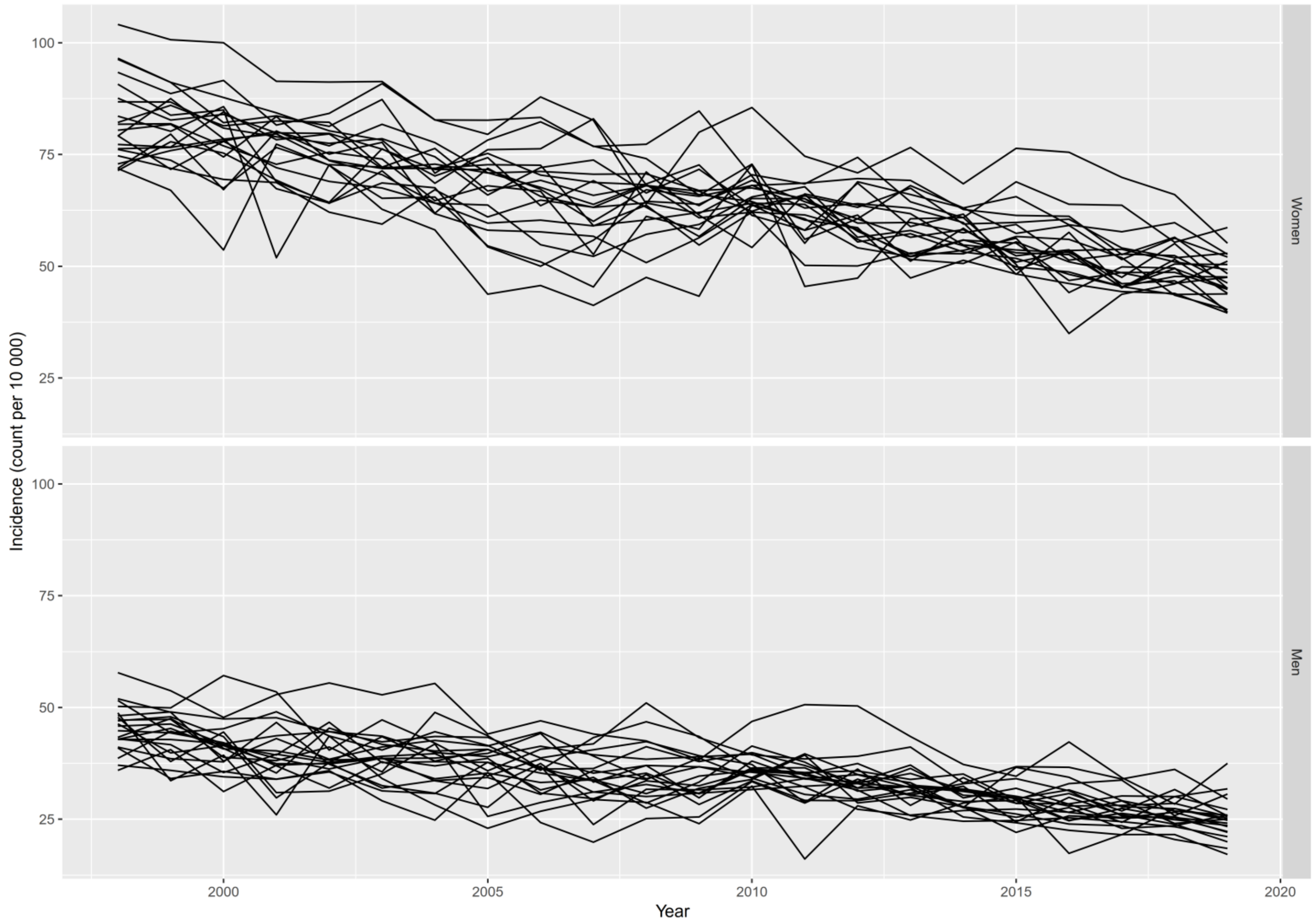

Supplementary Figure 7. Age-standardized trends for incidence of hospital admissions for hip fractures predicted to be incident admissions, displayed separately for the 21 health-care regions in Sweden. The background population was restricted to 50 years of age or older.

Supplementary File 1. The SAS code for the identification of incident hip fractures. The algorithm is based on age, sex, diagnoses, time intervals between admissions, type of admission, type of injury, and type of hospital department (1).

Reference

1. Gedeborg R, Engquist H, Berglund L, Michaelsson K. Identification of incident injuries in hospital discharge registers. Epidemiology. 2008;19(6):860-7.

SAS code for data management of injury database, including identification of incident injury events.

```
Program:	Injury database from national Swedish Health Care Databases:
/*****************************************************************************************
Purpose:	Merge and check data from hospital discharge data and cause of death data
Created:	First version Dec 2005
Revised:	2021/11/25
By:		Rolf Gedeborg
Notes:		New corrected data from SoS March 2021. Files from SoS are
t_r_dors__7178_2020 and t_t_t_r_par_sv_7178_2020.

		OLD:
		This version amended after discovery of potential missing
cases after comparison hip fx incidence with aggregated data from SoS. Added code to describe trends over
time
		and verify identification of index cases algorithm works as
intended.

		Version revised after final data delivery April 2016 from SCB
and SoS. New delivery July 2021.
		Ver21: Sensitivity analysis defining linked hospital admission
using same date discharge/admission as criterion
*****************************************************************************************/

/* Set libraries */
libname eget  "\\micro.intra\projekt\P0660$\P0660_Gem"; run;
libname data  "\\micro.intra\projekt\P0660$\P0660_Gem"; run;
libname datasosd  "\\micro.intra\projekt\P0660$\P0660_Data\Leverans_20210322"; run;
libname datasos  "\\micro.intra\projekt\P0660$\P0660_Data\Leverans_20210831"; run;
libname egetimpo  "\\micro.intra\projekt\P0660$\P0660_Gem\Rolfs SAS program"; run;
libname vascular  "\\micro.intra\projekt\P0660$\P0660_Gem\Vascular"; run;
libname formats  "\\micro.intra\projekt\P0660$\P0660_Gem\Formats"; run;

options fmtsearch = (formats);

proc contents data = datasos.t_t_t_r_par_sv_7178_2020; title 'Dataset content: PAR (hosp discharge registry)';
run;
proc contents data = datasosd.t_r_dors__7178_2020; title 'Dataset content: DOR (cause of death register)';
run;

/* Check AR versus INDATUM variable */
```

```
data d_temp; set datasos.t_t_t_r_par_sv_7178_2020;
indate = input(indatuma, YYMMDD10.);
if AR ge 1997 AND AR NE . AND year(indate) = .; run;
/* Only 24 cases identified with AR but indatum missing. Reasonable to exclude these cases */

data d_temp; set datasos.t_t_t_r_par_sv_7178_2020;
indate = input(indatuma, YYMMDD10.);
if indate = .; run;

/* Check years included */
proc freq data = datasos.t_t_t_r_par_sv_7178_2020; table ar; title 'Dataset content: PAR (hosp discharge
data)'; run;
/*proc freq data = datasos.inlaggningar_Ny; table ar; title 'Dataset content: entire PAR'; run;*/
proc freq data = datasosd.t_r_dors__7178_2020; table ar; title 'Dataset content: Cause of death register'; run;
data d_temp; set datasosd.t_r_dors__7178_2020;
deathdate = input(dodsdat, YYMMDD10.); run;
proc tabulate data = d_temp; var deathdate; table deathdate, n nmiss (max min)* f=YYMMDD10.; title 'Check
span deathdate'; run;

/* Check length op variable */
data d_temp; set datasos.t_t_t_r_par_sv_7178_2020 (keep = op);
l = length(op); run;

proc freq data = d_temp; table l; title 'Check length op variable'; run;

/* Select admissions with injury as main dx */
data data.d_par_STmain98_final; set datasos.t_t_t_r_par_sv_7178_2020;
label val_indate = 'Validation indate';
label val_outdate = 'Validation outdate';
label val_bdate = 'Validation birthdate';
label val_allergy_adveff = 'Validation allergy adverse effects';
label val_maindx_1 = "Validation main dx missing";
label val_maindx_2 = "Validation main dx not in scope";

format val_indate val_outdate val_allergy_adveff val_maindx_1 val_maindx_2 check. indate outdate bdate
YYMMDD10.;

val_maindx_1 = 0; val_maindx_2 = 0; val_indate = 0; val_outdate = 0; val_allergy_adveff = 0;

if strip(hdia) = '' then val_maindx_1 = 1; *Tag records without a main dx;
                else do;
if substr(strip(hdia),1,1) not IN("S", "s", "T", "t", "8", "9") then val_maindx_2 = 1;
                end;
*if substr(strip(hdia),1,1) IN("S", "T");
                *If period with ICD-9 dx NOT included;

/* date only as numerical
if
                strip(indatuma) = '' OR strip(utdatuma) = '' OR
                length(strip(indatuma)) NE 8 OR length(strip(utdatuma)) NE 8 OR
                substr(strip(indatuma),8,1) NOT IN('0','1','2','3','4','5','6','7','8','9') OR
                substr(strip(utdatuma),8,1) NOT IN('0','1','2','3','4','5','6','7','8','9')
then val_inoutdate = 1;
format indate outdate YYMMDD10.;
if val_inoutdate = 0 then indate = MDY(substr(strip(indatuma),5,2), substr(strip(indatuma),7,2),
substr(strip(indatuma),1,4));      * new format admission date;
```

```
if val_inoutdate = 0 then outdate = MDY(substr(strip(utdatuma),5,2), substr(strip(utdatuma),7,2),
substr(strip(utdatuma),1,4));      * new format discharge date;
*/

/* Rename date and geo variables */
indate = input(indatuma, YYMMDD10.); outdate = input(utdatuma, YYMMDD10.);
bdate = mdy(substr(strip(foddat),5,2), '15', substr(strip(foddat),1,4));

if strip(lk) NE '' then geo = lk; if strip(lk) = '' then geo = lkf;

year = ar;

if indate = . then val_indate = 1;
if outdate = . then val_outdate = 1;
if bdate = . then val_bdate = 1;

/* Check for allergy / adverse effects cases */
if length(hdia) GE 3 then do;
        if (substr(strip(hdia),1,3) eq 'T78' OR substr(strip(hdia),1,3) ge 'T80') then val_allergy_adveff =
1;                 *If period with ICD-9 dx NOT included;
end;

if year > 1997;   *Restrict data on index injuries to ICD-10 period from 1998;

drop indatuma utdatuma foddat lk lkf ar ;
run;

/* Check effects of selection criteria for indate, main dx */
/* proc contents data = data.d_par_STmain98_final; title 'Data content dataset selected main dx injury after
1997'; run; */
proc freq data = data.d_par_STmain98_final; table val_indate val_outdate val_bdate val_allergy_adveff
val_maindx_1 val_maindx_1*year val_maindx_2 geo / missing;
        format val_maindx_1 val_maindx_1 val_maindx_2 val_indate val_outdate val_allergy_adveff
check.; title 'Validity check variables for dataset selection'; run;

/* Check excluded cases */
data d_temp; set data.d_par_STmain98_final; if val_maindx_1 = 1; run;
proc freq data = d_temp; table hdia dia1 / missing; title 'Main diagnosis missing'; run;

data d_temp; set data.d_par_STmain98_final; if val_maindx_2 = 1; run;
proc freq data = d_temp; table hdia / missing; title 'Main diagnoses excluded from the study population'; run;
/* Also including cases with HDIA where lower case (and not only upper case) S or T in the first position was
included added 101,185 cases! */

data d_temp; set data.d_par_STmain98_final; if val_allergy_adveff = 1; run;
proc freq data = d_temp; table hdia / missing; title 'Dx excluded from the study population'; run;

/* Exclude cases without valid main dx, indate and cases with main dx of allergy or adverse effects */
data data.d_par_STmain98_final; set data.d_par_STmain98_final;
        if val_maindx_1 = 0 AND val_maindx_2 = 0 AND val_indate = 0 AND val_outdate = 0 AND
val_allergy_adveff = 0;
        drop val_maindx_1 val_maindx_2 val_indate val_outdate val_allergy_adveff; run;

/* Create a file with deaths having a cause code in variable chapt19 */
proc contents data = datasosd.t_r_dors__7178_2020; run;

data data.injdead; set datasosd.t_r_dors__7178_2020;
```

```
if kap19 NE ''; run;

/* !DATA ENTRY: Which dataset contains raw data from hospital discharge database - subset
		with injury admissions (main diag from chapt 19 ICD-10) from specified date range: */
%let datalib = data;
%let tabpatreg = d_par_STmain98_final;

/* !DATA ENTRY: Which dataset contains raw data from hospital discharge database - subset
		with all admissions irrespective of main diag for patients in subset above: */
data data.par; set datasos.t_t_t_r_par_sv_7178_2020; run;
%let tabcomorb = par;

/* !DATA ENTRY: Which dataset contains raw data from Cause of death register - subset
		with all patients present in first subset above: */
data data.dors; set datasosd.t_r_dors__7178_2020; run;
%let taballdead = dors;

/* !DATA ENTRY: Which dataset contains raw data from Cause of death register - subset
		with all patients dead from trauma, also without corresponding hospital admission: */
%let tabinjurydead = injdead;


/* CHAPTER 4- Merge all records corresponding to the same injury admission
		------------------------------------------------------------------------------------------
   - Create variable that numbers records corresponding to one injury admission (dep_change)
   - Create variable for interval from preceeding admission (adm_intval)
   - Create new variables for diagnoses, op.procedures codes, causecode */

		/* Merge dataset with admissions with principal dx from chapt 19 ICD-10 with dataset
		containing all hospital admissions (irrespective of main diag) for those individals. Remove
duplicate records. */

data d3d; set &datalib..&tabcomorb.;
indate = input(indatuma, YYMMDD10.); outdate = input(utdatuma, YYMMDD10.);
if indate lt '01jan1998'd then delete; *remove admission outside specified date range;
if indate gt '31dec2019'd then delete;
drop indatuma utdatuma; run;

data work.d3a; set &datalib..&tabpatreg. d3d; run;

proc sort data = work.d3a nodupkey; by lopnr indate outdate; run; *remove duplicates;

data d3; set work.d3a; title 'Number of records per injury';
format adm_intval 6. tmp_date YYMMDD10.; adm_intval = .;
label dep_change = 'Counter department/hospital change';
label adm_intval = 'Interval subsequent admissions';
		by lopnr;
		retain dep_change tmp_date; if first.lopnr then dep_change = 0;
		if first.lopnr then tmp_date = .;
		if indate - tmp_date < 1 then
						dep_change = dep_change + 1;
			else do;
						dep_change = 1;
						adm_intval = indate - tmp_date;
			end;
		tmp_date = outdate;
		drop tmp_date; run;
```

```
data data.d3; set d3;
			retain adm_no 0;
			if dep_change = 1 then adm_no = adm_no + 1; run;
proc contents data = data.d3; run;
proc sort data = data.d3; by adm_no dep_change; run;

data d_temp; set data.d3; keep dep_change; run;
proc freq data = d_temp; table dep_change; run;

/* Calculate total los */
data d3a; set data.d3 (keep = lopnr adm_no dep_change indate outdate);
format los 5.; label los = 'Length of stay (days)';
los = intck('day', indate, outdate); if los = 0 then los = 0.5;
run;

proc summary data = d3a; by lopnr adm_no; var los; output out = d3b SUM = sumlos; run;

proc sort data = d3a; by lopnr adm_no; run;
data d3c; merge data.d3 (IN = a) d3b (IN = b); by lopnr adm_no; if a; run;

/* Check missing rate among variables with diagnoses and ekodes to detect empty variables */
ods output Nlevels = checkchar;
ods html close;
proc freq data = d3c nlevels; tables _char_ / noprint; run;
ods html;
ods output close;

data d4a;
/* Set length of op variable to accomodate length as determined above. In this case longest 179 */
length op1-op10 $ 180;

set d3c; by adm_no dep_change;
if dep_change > 10 then delete; * Delete outlier records with dep_change>10. Will not bias total los which was
calculated above;

/* Split "op" variable
array a_op[*] $ op1 - op30;
do i = 1 to 30;
			a_op[i] = scan(op, i, ' ');
end; */

array a_hdia(10) $ hdia_1-hdia_10;
array a_diag1(10) $ diag1_1-diag1_10;
array a_diag2(10) $ diag2_1-diag2_10;
array a_diag3(10) $ diag3_1-diag3_10;
array a_diag4(10) $ diag4_1-diag4_10;
array a_diag5(10) $ diag5_1-diag5_10;
array a_diag6(10) $ diag6_1-diag6_10;
array a_diag7(10) $ diag7_1-diag7_10;
array a_diag8(10) $ diag8_1-diag8_10;
array a_diag9(10) $ diag9_1-diag9_10;
array a_diag10(10) $ diag10_1-diag10_10;
array a_diag11(10) $ diag11_1-diag11_10;
array a_diag12(10) $ diag12_1-diag12_10;
array a_diag13(10) $ diag13_1-diag13_10;
array a_diag14(10) $ diag14_1-diag14_10;
```

```
array a_diag15(10) $ diag15_1-diag15_10;
array a_diag16(10) $ diag16_1-diag16_10;
array a_diag17(10) $ diag17_1-diag17_10;
array a_diag18(10) $ diag18_1-diag18_10;
array a_diag19(10) $ diag19_1-diag19_10;
array a_diag20(10) $ diag20_1-diag20_10;
array a_diag21(10) $ diag21_1-diag21_10;
array a_diag22(10) $ diag22_1-diag22_10;
array a_diag23(10) $ diag23_1-diag23_10;
array a_diag24(10) $ diag24_1-diag24_10;
array a_diag25(10) $ diag25_1-diag25_10;
array a_diag26(10) $ diag26_1-diag26_10;
array a_diag27(10) $ diag27_1-diag27_10;
array a_diag28(10) $ diag28_1-diag28_10;
array a_diag29(10) $ diag29_1-diag29_10;
array a_diag30(10) $ diag30_1-diag30_10;

/*
array a_opd01(10) opd01_1-opd01_10;
array a_opd02(10) opd02_1-opd02_10;
array a_opd03(10) opd03_1-opd03_10;
array a_opd04(10) opd04_1-opd04_10;
array a_opd05(10) opd05_1-opd05_10;
array a_opd06(10) opd06_1-opd06_10;
array a_opd07(10) opd07_1-opd07_10;
array a_opd08(10) opd08_1-opd08_10;
array a_opd09(10) opd09_1-opd09_10;
array a_opd10(10) opd10_1-opd10_10;
array a_opd11(10) opd11_1-opd11_10;
array a_opd12(10) opd12_1-opd12_10;
array a_opd13(10) opd13_1-opd13_10;
array a_opd14(10) opd14_1-opd14_10;
array a_opd15(10) opd15_1-opd15_10;
array a_opd16(10) opd16_1-opd16_10;
array a_opd17(10) opd17_1-opd17_10;
array a_opd18(10) opd18_1-opd18_10;
array a_opd19(10) opd19_1-opd19_10;
array a_opd20(10) opd20_1-opd20_10;
array a_opd21(10) opd21_1-opd21_10;
array a_opd22(10) opd22_1-opd22_10;
array a_opd23(10) opd23_1-opd23_10;
array a_opd24(10) opd24_1-opd24_10;
array a_opd25(10) opd25_1-opd25_10;
array a_opd26(10) opd26_1-opd26_10;
array a_opd27(10) opd27_1-opd27_10;
array a_opd28(10) opd28_1-opd28_10;
array a_opd29(10) opd29_1-opd29_10;
array a_opd30(10) opd30_1-opd30_10;
*/

array a_ekod1(10) $ ekod1_1-ekod1_10;
array a_ekod2(10) $ ekod2_1-ekod2_10;
array a_ekod3(10) $ ekod3_1-ekod3_10;
array a_ekod4(10) $ ekod4_1-ekod4_10;
array a_ekod5(10) $ ekod5_1-ekod5_10;

array a_op (10) op1-op10;
```

```
array a_indate (10) indate1-indate10;
array a_adm_status (10) $ adm_status1-adm_status10;
array a_mvo (10) $ mvo1-mvo10;
array a_outdate (10) outdate1-outdate10;
array a_disch_status (10) $ disch_status1-disch_status10;
array a_lt_klin (10) $ lt_klin1-lt_klin10;

%LET alldiag = hdia_1-hdia_10
diag1_1-diag1_10
diag2_1-diag2_10
diag3_1-diag3_10
diag4_1-diag4_10
diag5_1-diag5_10
diag6_1-diag6_10
diag7_1-diag7_10
diag8_1-diag8_10
diag9_1-diag9_10
diag10_1-diag10_10
diag11_1-diag11_10
diag12_1-diag12_10
diag13_1-diag13_10
diag14_1-diag14_10
diag15_1-diag15_10
diag16_1-diag16_10
diag17_1-diag17_10
diag18_1-diag18_10
diag19_1-diag19_10
diag20_1-diag20_10
diag21_1-diag21_10
diag22_1-diag22_10
diag23_1-diag23_10
diag24_1-diag24_10
diag25_1-diag25_10
diag26_1-diag26_10
diag27_1-diag27_10
diag28_1-diag28_10
diag29_1-diag29_10
diag30_1-diag30_10;

%LET allcause =
ekod1_1-ekod1_10
ekod2_1-ekod2_10
ekod3_1-ekod3_10
ekod4_1-ekod4_10
ekod5_1-ekod5_10;

%LET allindate = indate1-indate10;
%LET alloutdate = outdate1-outdate10;

%LET allop = op1-op10;
/*
%LET allopd = opd01_1-opd01_10 opd02_1-opd02_10 opd03_1-opd03_10 opd04_1-opd04_10 opd05_1-
opd05_10 opd06_1-opd06_10
                        opd07_1-opd07_10 opd08_1-opd08_10 opd09_1-opd09_10 opd10_1-opd10_10 opd11_1-
opd11_10 opd12_1-opd12_10 opd12_1-opd12_10 opd12_1-opd12_10
 opd12_1-opd12_10 opd12_1-opd12_10 opd12_1-opd12_10 opd12_1-opd12_10 opd12_1-opd12_10 opd12_1-
opd12_10 opd12_1-opd12_10 opd13_1-opd13_10
```

```
 opd14_1-opd14_10 opd15_1-opd15_10 opd16_1-opd16_10 opd17_1-opd17_10 opd18_1-opd18_10 opd19_1-
opd19_10 opd20_1-opd20_10
 opd21_1-opd21_10 opd22_1-opd22_10 opd23_1-opd23_10 opd24_1-opd24_10 opd25_1-opd25_10 opd26_1-
opd26_10 opd27_1-opd27_10
 opd28_1-opd28_10 opd29_1-opd29_10 opd30_1-opd30_10;
*/

%LET allmvo = mvo1-mvo10;
%LET alldisch_status = disch_status1-disch_status10;
%LET alladm_status = adm_status1-adm_status10;
%LET alllt_klin = lt_klin1-lt_klin10;


retain &alldiag &allcause &allindate &alloutdate &alllt_klin &allop &allmvo &alladm_status &alldisch_status
                        &alllt_klin;
            if first.adm_no then
                        do i=1 to 10; a_hdia(i)='';
                                    a_diag1(i)=''; a_diag2(i)=''; a_diag3(i)=''; a_diag4(i)='';
a_diag5(i)='';
                                    a_diag6(i)=''; a_diag7(i)=''; a_diag8(i)=''; a_diag9(i)='';
a_diag10(i)='';
                                    a_diag11(i)=''; a_diag12(i)=''; a_diag13(i)=''; a_diag14(i)='';
a_diag15(i)='';
                                    a_diag16(i)=''; a_diag17(i)=''; a_diag18(i)=''; a_diag19(i)='';
a_diag20(i)='';
                                    a_diag21(i)=''; a_diag22(i)=''; a_diag23(i)=''; a_diag24(i)='';
a_diag25(i)='';
                                    a_diag26(i)=''; a_diag27(i)=''; a_diag28(i)=''; a_diag29(i)='';
a_diag30(i)='';
                                    a_ekod1(i)=''; a_ekod2(i)=''; a_ekod3(i)=''; a_ekod4(i)='';
a_ekod5(i)='';
                                    a_indate(i)=.; a_outdate(i)=.; a_lt_klin(i)=''; a_adm_status(i)='';
a_mvo(i)='';
                                    a_disch_status(i)=''; a_op(i) ='';
                                    /* a_opd01(i) =.; a_opd02(i) =.; a_opd03(i) =.; a_opd04(i) =.;
a_opd05(i) =.;
                                    a_opd06(i) =.; a_opd07(i) =.; a_opd08(i) =.; a_opd09(i) =.;
a_opd10(i) =.; a_opd11(i) =.; a_opd12(i) =.;
                                    a_opd13(i) =.; a_opd14(i) =.; a_opd15(i) =.; a_opd16(i) =.;
a_opd17(i) =.; a_opd18(i) =.; a_opd19(i) =.;
                                    a_opd20(i) =.; a_opd21(i) =.; a_opd22(i) =.; a_opd23(i) =.;
a_opd24(i) =.; a_opd25(i) =.; a_opd26(i) =.;
                                    a_opd27(i) =.; a_opd28(i) =.; a_opd29(i) =.; a_opd30(i) =.;*/
                        end;

            a_hdia(dep_change) = hdia;
            a_diag1(dep_change) = dia1;
            a_diag2(dep_change) = dia2;
            a_diag3(dep_change) = dia3;
            a_diag4(dep_change) = dia4;
            a_diag5(dep_change) = dia5;
            a_diag6(dep_change) = dia6;
            a_diag7(dep_change) = dia7;
            a_diag8(dep_change) = dia8;
            a_diag9(dep_change) = dia9;
            a_diag10(dep_change) = dia10;
            a_diag11(dep_change) = dia11;
```

```
a_diag12(dep_change) = dia12;
a_diag13(dep_change) = dia13;
a_diag14(dep_change) = dia14;
a_diag15(dep_change) = dia15;
a_diag16(dep_change) = dia16;
a_diag17(dep_change) = dia17;
a_diag18(dep_change) = dia18;
a_diag19(dep_change) = dia19;
a_diag20(dep_change) = dia20;
a_diag22(dep_change) = dia22;
a_diag22(dep_change) = dia22;
a_diag23(dep_change) = dia23;
a_diag24(dep_change) = dia24;
a_diag25(dep_change) = dia25;
a_diag26(dep_change) = dia26;
a_diag27(dep_change) = dia27;
a_diag28(dep_change) = dia28;
a_diag29(dep_change) = dia29;
a_diag30(dep_change) = dia30;

a_ekod1(dep_change) = ekod1;
a_ekod2(dep_change) = ekod2;
a_ekod3(dep_change) = ekod3;
a_ekod4(dep_change) = ekod4;
a_ekod5(dep_change) = ekod5;

a_op(dep_change) = op;
/*a_opd01(dep_change) = opd1;
a_opd02(dep_change) = opd2;
a_opd03(dep_change) = opd3;
a_opd04(dep_change) = opd4;
a_opd05(dep_change) = opd5;
a_opd06(dep_change) = opd6;
a_opd07(dep_change) = opd7;
a_opd08(dep_change) = opd8;
a_opd09(dep_change) = opd9;
a_opd10(dep_change) = opd10;
a_opd11(dep_change) = opd11;
a_opd12(dep_change) = opd12;
a_opd13(dep_change) = opd13;
a_opd14(dep_change) = opd14;
a_opd15(dep_change) = opd15;
a_opd16(dep_change) = opd16;
a_opd17(dep_change) = opd17;
a_opd18(dep_change) = opd18;
a_opd19(dep_change) = opd19;
a_opd20(dep_change) = opd20;
a_opd21(dep_change) = opd21;
a_opd22(dep_change) = opd22;
a_opd23(dep_change) = opd23;
a_opd24(dep_change) = opd24;
a_opd25(dep_change) = opd25;
a_opd26(dep_change) = opd26;
a_opd27(dep_change) = opd27;
a_opd28(dep_change) = opd28;
a_opd29(dep_change) = opd29;
a_opd30(dep_change) = opd30;*/
```

```
                    a_indate(dep_change) = indate;
                    a_outdate(dep_change) = outdate;
                    a_mvo(dep_change) = mvo;
                    a_lt_klin(dep_change) = lt_klin;
                    a_adm_status(dep_change) = insatt;
                    a_disch_status(dep_change) = utsatt;

                    if last.adm_no then output; keep lopnr bdate kon fland geo pvard adm_no adm_intval
dep_change pvard &alldiag
                     &allcause &allindate &alloutdate &alllt_klin &allop &allmvo &alladm_status &alldisch_status;

label lopnr = 'ID';
run;

/* proc contents data = d4a; run; */

/* Display number of admissions by year before and after creating chains of linked admissions */
proc freq data = d3c; table year / plots = freqplot; title 'Number of admissions by year'; run;
data d4ax; set d4a (keep = indate1); year = year(indate1); run;
proc freq data = d4ax; table year / plots = freqplot; title 'Number of admissions by year'; run;

/* Check records with year <1997 */
data d_temp; set d4a (keep = hdia_1 indate1); year = year(indate1); if year < 1998; run;
proc freq data = d_temp order = freq; table hdia_1; title 'Distribution of main dx among admission chains with
indate before 1998'; run;
/* 4701 records with admission date before 1998 to be deleted below. Injury while admitted to hospital.
Mainly psychiatric diagnoses and cerebral infarction. */

proc datasets lib = work nolist; * Delete temporary file;
                    delete d3 d3a d3b d3c; run; quit;

proc freq data = d4a order = freq; table hdia_1; title 'Distribution of main dx among admission chains'; run;

/* Selection of data. This removes records where the injury occured during a hospital stay. */
data d4b; set d4a;
if strip(hdia_1) = '' then delete;
if substr(strip(hdia_1),1,1) not in('S', 's', 'T', 't', '8', '9') then delete;
year = year(indate1); if year < 1998 then delete;
run;

proc datasets lib = work nolist; * Delete temporary file;
                    delete d4a d4ax; run; quit;

/* Rename diagnosis (and opcode variables / not in this version) */
data data.trauma_index; set d4b;
                    array a_alldiag(*) $ &alldiag;
                    array a_newdiag(*) $ dx1-dx310;
                    do i=1 to 310;
                                        a_newdiag(i)=a_alldiag(i);
                    end;
                    drop i &alldiag; run;

/* CHAPTER 5 - Check data from Cause of death register for accuracy
--------------------------------------------------------------------------------------------- */
proc datasets lib = work nolist; * Delete temporary file no longer in use;
                    delete d4b; run; quit;
```

```
/* CHAPTER 6 - Continue data check:
                    ----------------------------------------------------------------------------------------------
                    - Are there records with invalid main diagnosis?
                    * First 4 positions in dx code should match ICD10code in matrix definition
                    * List incorrect codes
                    * Repare incorrect codes */

data d6; set &datalib..&taballdead. &datalib..&tabinjurydead.(keep = ulorsak morsak1-morsak48 kap19);
array arr_diag[*] $ ulorsak morsak1-morsak48 kap19;
do  n=1 to 50;
                    if substr(arr_diag[n],1,1) in('S' 'T') then ICD10code = compress(SUBSTR(arr_diag[n],1,4), "-");
output;
end;
drop n ulorsak morsak1-morsak48 kap19; run;

proc sort data = d6 out = d6a; by ICD10code; run;

data d_diaglist; set data.D_transl_4; temp_ICD10code = PUT(ICD10code,$6.); drop ICD10code; run;

data d_diaglist2; set d_diaglist; ICD10code = temp_ICD10code; drop temp_ICD10code; run;

data d6b; merge d6a(in=a) d_diaglist2(in=b); by ICD10code; if a AND NOT b;
                    if substr(ICD10code,1,3) eq 'T78' then delete;                    * remove allergy from check;
                    if substr(ICD10code,1,3) ge 'T80' then delete;                    * remove adverse and late
effects;
keep ICD10code; run;

proc freq data = d6b; tables ICD10code / out = data.freqcount_faultyICD10_dead;
Title 'Listing of injury ICD10-codes in Cause-of-death data not in injury matrix definition';
run;

proc datasets lib = work nolist;
                    * Delete temporary file no longer in use;
                    delete d6 d6a d6b d_diaglist d_diaglist2; run; quit;

/* !EVALUATION POINT: Note incorrect diagnosis codes from output above */

/* CHAPTER 7 - Mend dataset diagnoses according to the result.
                    -------------------------------------------------------------------------------------------
/* !DATA ENTRY:  Enter faulty codes from result above and correct codes below*/

data d7_alldead; set &datalib..&taballdead.;
array arr_diag[*] $ ulorsak morsak1-morsak48 kap19;
do  n = 1 to 50;
                    if SUBSTR(arr_diag[n],1,4)='S00' then arr_diag[n]='S009';
                    if SUBSTR(arr_diag[n],1,4)='S10' then arr_diag[n]='S109';
                    if SUBSTR(arr_diag[n],1,4)='S20' then arr_diag[n]='S209';
                    if SUBSTR(arr_diag[n],1,4)='S30' then arr_diag[n]='S309';
                    if SUBSTR(arr_diag[n],1,4)='S40' then arr_diag[n]='S409';
                    if SUBSTR(arr_diag[n],1,4)='S50' then arr_diag[n]='S509';
                    if SUBSTR(arr_diag[n],1,4)='S60' then arr_diag[n]='S609';
                    if SUBSTR(arr_diag[n],1,4)='S70' then arr_diag[n]='S709';
                    if SUBSTR(arr_diag[n],1,4)='S80' then arr_diag[n]='S809';
                    if SUBSTR(arr_diag[n],1,4)='S90' then arr_diag[n]='S909';
                    if SUBSTR(arr_diag[n],1,4)='T00' then arr_diag[n]='T009';
                    if SUBSTR(arr_diag[n],1,4)='T08' then arr_diag[n]='T089';
```

```
            if SUBSTR(arr_diag[n],1,4)='T080' then arr_diag[n]='T089';
            if SUBSTR(arr_diag[n],1,4)='T10' then arr_diag[n]='T109';
            if SUBSTR(arr_diag[n],1,4)='T100' then arr_diag[n]='T109';
            if SUBSTR(arr_diag[n],1,4)='T120' then arr_diag[n]='T129';
            if SUBSTR(arr_diag[n],1,4)='T15' then arr_diag[n]='T159';
            if SUBSTR(arr_diag[n],1,4)='T20' then arr_diag[n]='T209';
            if SUBSTR(arr_diag[n],1,4)='T33' then arr_diag[n]='T339';
            if SUBSTR(arr_diag[n],1,4)='T36' then arr_diag[n]='T369';
            if SUBSTR(arr_diag[n],1,4)='T51' then arr_diag[n]='T519';
            if SUBSTR(arr_diag[n],1,4)='T68' then arr_diag[n]='T689';
            if SUBSTR(arr_diag[n],1,4)='T79' then arr_diag[n]='T799';
            if SUBSTR(arr_diag[n],1,4)='S16' then arr_diag[n]='S169';
            if SUBSTR(arr_diag[n],1,4)='S18' then arr_diag[n]='S189';
            if SUBSTR(arr_diag[n],1,4)='T07' then arr_diag[n]='T079';
            if SUBSTR(arr_diag[n],1,4)='T100' then arr_diag[n]='T109';
            if SUBSTR(arr_diag[n],1,4)='T12' then arr_diag[n]='T129';
            if SUBSTR(arr_diag[n],1,4)='T120' then arr_diag[n]='T129';
            if SUBSTR(arr_diag[n],1,4)='T55' then arr_diag[n]='T559';
            if SUBSTR(arr_diag[n],1,4)='T58' then arr_diag[n]='T589';
            if SUBSTR(arr_diag[n],1,4)='T64' then arr_diag[n]='T649';
            if SUBSTR(arr_diag[n],1,4)='T66' then arr_diag[n]='T669';
            if SUBSTR(arr_diag[n],1,4)='T68' then arr_diag[n]='T689';
            if SUBSTR(arr_diag[n],1,4)='T71' then arr_diag[n]='T719';
end;
drop n kon; run;

data d7_injurydead; set &datalib..&tabinjurydead.;
array arr_diag[*] $ morsak1-morsak48 ulorsak kap19;
do  n=1 to 50;
            if SUBSTR(arr_diag[n],1,4)='S00' then arr_diag[n]='S009';
            if SUBSTR(arr_diag[n],1,4)='S10' then arr_diag[n]='S109';
            if SUBSTR(arr_diag[n],1,4)='S20' then arr_diag[n]='S209';
            if SUBSTR(arr_diag[n],1,4)='S30' then arr_diag[n]='S309';
            if SUBSTR(arr_diag[n],1,4)='S40' then arr_diag[n]='S409';
            if SUBSTR(arr_diag[n],1,4)='S50' then arr_diag[n]='S509';
            if SUBSTR(arr_diag[n],1,4)='S60' then arr_diag[n]='S609';
            if SUBSTR(arr_diag[n],1,4)='S70' then arr_diag[n]='S709';
            if SUBSTR(arr_diag[n],1,4)='S80' then arr_diag[n]='S809';
            if SUBSTR(arr_diag[n],1,4)='S90' then arr_diag[n]='S909';
            if SUBSTR(arr_diag[n],1,4)='T00' then arr_diag[n]='T009';
            if SUBSTR(arr_diag[n],1,4)='T08' then arr_diag[n]='T089';
            if SUBSTR(arr_diag[n],1,4)='T080' then arr_diag[n]='T089';
            if SUBSTR(arr_diag[n],1,4)='T10' then arr_diag[n]='T109';
            if SUBSTR(arr_diag[n],1,4)='T100' then arr_diag[n]='T109';
            if SUBSTR(arr_diag[n],1,4)='T120' then arr_diag[n]='T129';
            if SUBSTR(arr_diag[n],1,4)='T15' then arr_diag[n]='T159';
            if SUBSTR(arr_diag[n],1,4)='T20' then arr_diag[n]='T209';
            if SUBSTR(arr_diag[n],1,4)='T33' then arr_diag[n]='T339';
            if SUBSTR(arr_diag[n],1,4)='T36' then arr_diag[n]='T369';
            if SUBSTR(arr_diag[n],1,4)='T51' then arr_diag[n]='T519';
            if SUBSTR(arr_diag[n],1,4)='T68' then arr_diag[n]='T689';
            if SUBSTR(arr_diag[n],1,4)='T79' then arr_diag[n]='T799';
            if SUBSTR(arr_diag[n],1,4)='S16' then arr_diag[n]='S169';
            if SUBSTR(arr_diag[n],1,4)='S18' then arr_diag[n]='S189';
            if SUBSTR(arr_diag[n],1,4)='T07' then arr_diag[n]='T079';
            if SUBSTR(arr_diag[n],1,4)='T100' then arr_diag[n]='T109';
            if SUBSTR(arr_diag[n],1,4)='T12' then arr_diag[n]='T129';
```

```
            if SUBSTR(arr_diag[n],1,4)='T120' then arr_diag[n]='T129';
            if SUBSTR(arr_diag[n],1,4)='T55' then arr_diag[n]='T559';
            if SUBSTR(arr_diag[n],1,4)='T58' then arr_diag[n]='T589';
            if SUBSTR(arr_diag[n],1,4)='T64' then arr_diag[n]='T649';
            if SUBSTR(arr_diag[n],1,4)='T66' then arr_diag[n]='T669';
            if SUBSTR(arr_diag[n],1,4)='T68' then arr_diag[n]='T689';
            if SUBSTR(arr_diag[n],1,4)='T71' then arr_diag[n]='T719';
end;

age_d = alder;
drop n alder; run;

proc contents data = d7_alldead; run;

/* !EVALUATION POINT: To verify that the mend is correct - run check in chapter 6 again with
   the mended datasets d7_alldead and d7_injurydead. Change library for permanent dataset with
   faulty codes when doing this not overwrite it*/

/* CHAPTER 8 - Merge data from hospital discharge data with data from Cause of death register
-------------------------------------------------------------------------------------------*/

proc sort data = data.trauma_index; by lopnr; run;
proc sort data = d7_alldead nodupkey; by lopnr; run;

/* Merge data from hospital discharge register with extraction from cause of death register
   of patients present in the hospital discharge register*/
data d8_disch_alldead;
            merge data.trauma_index (in = a) d7_alldead (in = b); by lopnr;
            if dep_change = . then delete; run; *delete deaths not related to a previuos hosp admission;

/* Merge this combined dataset with extract from cause of death register of patients with
   injury diagnosis as cause of death. This adds patients dead before arrival to hospital but
   if the patient has a previous hospital admission the record from the cause of death
   register is appended to the record from the hospital admission and not added as a prehospital
   index injury death. In this first step only deaths for patients already in the discharge
   register are kept. Dep_change = . indicates that there is no corresponding hospital admission
   for that patient. */

proc sort data = d7_injurydead; by lopnr; run;

data d8b;
            merge d8_disch_alldead (in = a) d7_injurydead (in = b); by lopnr; if a OR b;
            if dep_change = . then delete; run;

/* In this next step all prehospital index injury deaths are added to the dataset. A new
   variable for the hospital discharge date is created for sorting the records so that
   records of prehospital index injury deaths are put last in the list for each individual
   (lopnr). */

data d8c; set d8_disch_alldead d7_injurydead;
            outdate = max(of outdate1-outdate10);
            sortdate = outdate; if sortdate = . then sortdate = '31dec2019'd; run;

proc datasets lib = work nolist;
            delete d7_alldead d7_injurydead d8_disch_alldead d8b; run;

proc sort data = work.d8c; by lopnr sortdate; run;
```

```
/*  A record added from the cause of death register (by the SET statement above) is
    deleted if it is associated with a previous hospital admission. This is detected by
    comparing the death date with the hospital discharge date for that individual in a
    retain step. A flag (delete_var) is set to 1. If the individual has a previous admission
            the interval from discharge to prehospital death is calculated and added to the record. */

data data.d8d; set d8c; format deathdate tempdate outdate indate1-indate10 outdate1-outdate10
YYMMDD10.;
/* Fix unknown day or month for death. create variable deathdatefixed that indicates if day (=1) or also month
(=2)
            was unknown */
            if substr(dodsdat,5,2) = '00' then dodsdatnew1 =
substr(dodsdat,1,4)||'06'||substr(dodsdat,7,2);
                        else dodsdatnew1 = dodsdat;
            if substr(dodsdatnew1,7,2)= '00' then dodsdatnew = substr(dodsdatnew1,1,6)||'15';
                        else dodsdatnew = dodsdatnew1;
            deathdatefixed = 0;
            if substr(dodsdat,7,2) = '00' then deathdatefixed = 1;
            if substr(dodsdat,5,2)='00' then deathdatefixed = 2;

            if dodsdat NE '' then deathdate = MDY(substr(strip(dodsdatnew),5,2),

                                                substr(strip(dodsdatnew),7,2),

                                                            substr(strip(dodsdatnew),1,4));
            * new format death date;

if first.lopnr and deathdate NE . then tempdate = 0 and mort_intval = 0;
by lopnr;
retain tempdate mort_intval;
if (deathdate - tempdate) < 2 and outdate = . then delete_var = 1;
            else do
                        delete_var = 0;
                        mort_intval = deathdate - tempdate;
            end;
if tempdate = 0 then mort_intval = 0; tempdate = outdate;
drop dodsdatnew1 dodsdatnew; run;

data d8e; set data.d8d;
            if dep_change = . then ekod1_1 = ulorsak;
            if dep_change = . then indate1 = deathdate;
            if dep_change = . then adm_status1 = '4'; * adm_status = 4 is patient dead before admission to
hosp;
            if delete_var = 1 then delete; * Delete records of trauma deaths that have already been
                         appended on corresponding admission record;
            drop tempdate sortdate; run;

/* CHAPTER 9 - Create final traumapatient database
------------------------------------------------------------------------------------------*/
data d9a; set d8e;
/*Delete late effects of trauma from admission records;*/
            if substr(dx1,1,3) in('T78', 'T80', 'T81', 'T82', 'T83', 'T84', 'T85', 'T86', 'T87', 'T88', '995', '996',
'997', '998', '999')
            then delete; if substr(dx1,1,4) = 'T983' then delete;
/*Delete late effects of trauma from prehospital deaths records;*/
```

```
                if dep_change = . and substr(ulorsak,1,3) in('T78', 'T80', 'T81', 'T82', 'T83', 'T84', 'T85', 'T86',
'T87' 'T88','995', '996',


                '997', '998', '999')
                then delete; if dep_change = . and substr(ulorsak,1,4) = 'T983' then delete; run;

proc datasets lib = work nolist;
                delete d8e; run; quit;

/* Create variable for date for previous injury admission (prev_inj_date),
  date for next injury admission (date_nextinj), and number of injury admissions (no_injadm). */
proc sort data = d9a; by lopnr indate1; run;

data d9c; set d9a; by lopnr ; drop adm_intval;
                format tmp_admdate tmp_disdate prev_inj_admdate prev_inj_disdate yymmdd10.
inj_adm_no 2.;
                retain tmp_admdate tmp_disdate inj_adm_no;
                if first.lopnr then do prev_inj_admdate = .; prev_inj_disdate = .; inj_adm_no = 1; end;
                else do prev_inj_admdate = tmp_admdate; prev_inj_disdate = tmp_disdate; inj_adm_no + 1;
end;
                tmp_admdate = min(of indate1 - indate10); tmp_disdate = max(of outdate1-outdate10);
                drop tmp_admdate tmp_disdate; run;

proc sort data = d9c; by lopnr descending indate1; run;

data d9d; set d9c; by lopnr;
                format tmp_admdate subseq_inj_admdate yymmdd10.;
                retain tmp_admdate;
                if first.lopnr then subseq_inj_admdate = .; else subseq_inj_admdate = tmp_admdate;
                tmp_admdate = min(of indate1-indate10); drop tmp_admdate; run;

proc datasets lib = work nolist;
                delete d9c; run; quit;

proc sort data = d9d; by lopnr indate1; run;

data data.injury9819_final; set d9d;
adm_no = _n_; *Create variable for admissionnumber;
year = year(indate1);
if year < 1998 then delete;
drop year; run;

proc contents data = data.injury9819_final; run;

data d; set data.injury9819_final;
year = year(indate1);
weekday = weekday(indate1);
if dx1 NE '' then icd9 = 0; *Indicator for main dx coded ICD-9;
if substr(dx1,1,1) in('8','9') then icd9 = 1;
if dx1 NE '' then injurymaindx = 0; *Indicator for main dx being injury code;
if substr(dx1,1,1) in('S','T','8','9') then injurymaindx = 1;
death = 0; *Indicator for deathdate present;
if dodsdat NE '' then death = 1;
run;
```

```
proc freq data = d; table year weekday icd9 injurymaindx death year*icd9; title 'Number of index records per
year'; run;
proc freq data = d order = freq; table dx1 ekod1_1 lt_klin1; title 'Frequency dx and klin'; run;

/* Check final file for specific patient */
data check1; set d; if lopnr = 168; run;
data check2; set datasos.t_t_t_r_par_sv_7178_2020; if lopnr = 168; run;
data check3; set datasosd.t_r_dors__7178_2020; if lopnr = 168; run;
proc sort data = check1; by indate1; run;
proc sort data = check2; by indatuma; run;

proc contents data = datasosd.t_r_dors__7178_2020;


/* Create file with lopnr and indatum
data data.injuryindexcase_151207; set data.injury9814;
keep lopnr indate1; run;
proc contents data = data.injuryindexcase_151207; run; */


/*****************************************************************************************
**
Program:        Avancerad merge
Syfte:                          Komplettera data i en tabell med v‰rden frÂn en annan d‰r fˆrhÂllandet ‰r
        mÂnga till mÂnga.

Skapad:                         2006-06-08 Sˆren Gustafsson
fndrad:                         2009-08-29 Rolf Gedeborg (vetenskaplig applikation fˆr att extrahera info
                                        om comorbiditet frÂn Socialstyrelsens patientregister fˆr sluten
vÂrd)
÷vrigt:                         Programmet extraherar information frÂn ett best‰mt tidsintervall bakÂt frÂn
                                        inskrivningsdatum (i detta fall 5*365 dagar). Se
variabeldefinition nedan.

******************************************************************************************
*

Extract information on comorbidity from Swedish National Discharge Register (all admissions
5 years prior to injury admission). Conditions are defined both according to ICD-9 and
ICD-10. 4 new variables are created for each comorbidity (XXX denotes type of comorbidity):
- Number of admissions with main diagnosis: Co_XXX_nrmain
- Total length of stay (with main diagnosis): Co_XXX_los
- Date of last admissÌon with main diagnosis: Co_XXX_recentdatemain
- Number of admissions with subsidiary diagnosis: Co_XXX_nrsub

Create local copy of file with previous hospital admissions. Keep variables upin(personnummer)
adm_date(inskrivningsdatum), los(vÂrdtid), main_diag(huvuddiagnos) och bdia1-bdia7(bidiagnoser). */

data trauma_comorb1; set datasos.t_t_t_r_par_sv_7178_2020 (keep = lopnr indatuma utdatuma hdia dia1-
dia30);
if length(strip(indatuma)) = 8 then indatum = input(indatuma, YYMMDD10.);
if length(strip(utdatuma)) = 8 then utdatum = input(utdatuma, YYMMDD10.);
los = .; if indatum NE . AND utdatum NE . then los = datdif(indatum, utdatum, 'ACT/ACT');
hdiax = compress(upcase(strip(hdia)), "-");
drop hdia indatuma utdatuma utdatum; run;

/* Create index on lopnr(anonymt personnummer) */
```

```
proc sql;
		create index lopnr
		on trauma_comorb1;
quit;

/* Perform advanced merge to create new variables for comorbidity*/
data data.injury9819_co;
   set data.injury9819_final;
		retain sparid;
		if lopnr = sparid then do;
				lopnr = '';
		end;
		format Co_CHF_recentdatemain Co_IHD_recentdatemain Co_valv_recentdatemain
				Co_ary_recentdatemain Co_pulmcirc_recentdatemain Co_HT_recentdatemain
				Co_CVD_recentdatemain Co_perivasc_recentdatemain
Co_neurol_recentdatemain
				Co_CPD_recentdatemain Co_inf_recentdatemain Co_diab_recentdatemain
				Co_xendo_recentdatemain Co_renal_recentdatemain
Co_hepatic_recentdatemain
				Co_immundef_recentdatemain Co_hemomal_recentdatemain
Co_hemodis_recentdatemain
				Co_tumournomet_recentdatemain Co_tumourmet_recentdatemain
Co_collagen_recentdatemain
				Co_coag_recentdatemain Co_obese_recentdatemain Co_nutr_recentdatemain
				Co_fluid_recentdatemain Co_bleed_recentdatemain
Co_anemia_recentdatemain
				Co_alco_recentdatemain Co_drug_recentdatemain
Co_psycho_recentdatemain
				Co_affect_recentdatemain Co_xpsych_recentdatemain Co_tx_recentdatemain
				Co_bonemusc_recentdatemain Co_injury_recentdatemain
Co_intox_recentdatemain yymmdd8.
				hdiax dia1-dia30 $8.;

		/* set counters to zero */
		Co_CHF_nrmain=0; Co_CHF_los=0; Co_CHF_nrsub=0;
		Co_IHD_nrmain=0; Co_IHD_los=0; Co_IHD_nrsub=0;
		Co_valv_nrmain=0; Co_valv_los=0; Co_valv_nrsub=0;
		Co_ary_nrmain=0; Co_ary_los=0; Co_ary_nrsub=0;
		Co_pulmcirc_nrmain=0; Co_pulmcirc_los=0; Co_pulmcirc_nrsub=0;
		Co_HT_nrmain=0;			Co_HT_los=0; Co_HT_nrsub=0;
		Co_CVD_nrmain=0; Co_CVD_los=0; Co_CVD_nrsub=0;
		Co_perivasc_nrmain=0; Co_perivasc_los=0; Co_perivasc_nrsub=0;
		Co_neurol_nrmain=0;		Co_neurol_los=0; Co_neurol_nrsub=0;
		Co_CPD_nrmain=0; Co_CPD_los=0; Co_CPD_nrsub=0;
		Co_inf_nrmain=0; Co_inf_los=0; Co_inf_nrsub=0;
		Co_diab_nrmain=0; Co_diab_los=0; Co_diab_nrsub=0;
		Co_xendo_nrmain=0; Co_xendo_los=0; Co_xendo_nrsub=0;
		Co_renal_nrmain=0; Co_renal_los=0; Co_renal_nrsub=0;
		Co_hepatic_nrmain=0; Co_hepatic_los=0; Co_hepatic_nrsub=0;
		Co_immundef_nrmain=0; Co_immundef_los=0; Co_immundef_nrsub=0;
		Co_hemomal_nrmain=0; Co_hemomal_los=0; Co_hemomal_nrsub=0;
		Co_hemodis_nrmain=0; Co_hemodis_los=0; Co_hemodis_nrsub=0;
		Co_tumournomet_nrmain=0; Co_tumournomet_los=0; Co_tumournomet_nrsub=0;
		Co_tumourmet_nrmain=0; Co_tumourmet_los=0; Co_tumourmet_nrsub=0;
		Co_collagen_nrmain=0; Co_collagen_los=0; Co_collagen_nrsub=0;
		Co_coag_nrmain=0; Co_coag_los=0; Co_coag_nrsub=0;
		Co_obese_nrmain=0; Co_obese_los=0; Co_obese_nrsub=0;
```

```
Co_nutr_nrmain=0; Co_nutr_los=0; Co_nutr_nrsub=0;
Co_fluid_nrmain=0; Co_fluid_los=0; Co_fluid_nrsub=0;
Co_bleed_nrmain=0; Co_bleed_los=0; Co_bleed_nrsub=0;
Co_anemia_nrmain=0;             Co_anemia_los=0; Co_anemia_nrsub=0;
Co_alco_nrmain=0; Co_alco_los=0; Co_alco_nrsub=0;
Co_drug_nrmain=0; Co_drug_los=0; Co_drug_nrsub=0;
Co_psycho_nrmain=0; Co_psycho_los=0; Co_psycho_nrsub=0;
Co_affect_nrmain=0; Co_affect_los=0; Co_affect_nrsub=0;
Co_xpsych_nrmain=0; Co_xpsych_los=0; Co_xpsych_nrsub=0;
Co_tx_nrmain=0; Co_tx_los=0; Co_tx_nrsub=0;
Co_bonemusc_nrmain=0; Co_bonemusc_los=0; Co_bonemusc_nrsub=0;
Co_injury_nrmain=0; Co_injury_los=0; Co_injury_nrsub=0;
Co_intox_nrmain=0; Co_intox_los=0; Co_intox_nrsub=0;


array bdia[*] $ dia1-dia30; /* Define array for subsidiary diagnoses */
CALL MISSING (hdiax, of dia1-dia30, indatum, los); /* Set variables in lookup table to missing */
do until(klar = 1);
  /* Extra loop som tvingar fram l‰sning med id=missing om */
                /* samma nyckel finns flera ggr i taba=grundtabellen (h‰r


"datasafe.injury9804") */
do until(_iorc_=%sysrc(_dsenom)); /* Villkor som checkar om tr‰ff vid sˆkning */
        set trauma_comorb1 key = lopnr;
        select(_iorc_);
        when (%sysrc(_sok)) do;   /* Tr‰ff vid sˆkning. H‰r kan den egna koden
l‰ggas in */
                                        if indatum lt indate1 and /*
Define interval for extraction of information */
                                          indatum ge indate1-(365*5)
                                        then do;

/* Check for Congestive Heart Failure (CHF)*/
if substr(hdiax,1,3) in ('398','425','428','429','I13','I42','I43','I50','I51')
or substr(hdiax,1,4) in ('393A','402A','402X','404A','404B','404X')
then do; Co_CHF_nrmain+1; Co_CHF_recentdatemain=indatum; Co_CHF_los+los; end;
do i=1 to 30;
        if length(strip(bdia[i])) GE 3 then do;
                if substr(upcase(strip(bdia[i])),1,3) in
('398','425','428','429','I13','I42','I43','I50','I51')
                then Co_CHF_nrsub + 1;
        end;
        if length(strip(bdia[i])) GE 4 then do;
                if substr(upcase(strip(bdia[i])),1,4) in ('393A','402A','402X','404A','404B','404X')
                then Co_CHF_nrsub + 1;
        end;
end;

/* Check for Ischemic Heart Disease (IHD)*/
if substr(hdiax,1,3) in ('410','411','412','413','414','I20','I21','I22','I23','I24','I25')
then do; Co_IHD_nrmain+1; Co_IHD_recentdatemain=indatum; Co_IHD_los+los; end;
do i=1 to 30;
        if length(strip(bdia[i])) GE 3 then do;
                if substr(upcase(strip(bdia[i])),1,3) in
('410','411','412','413','414','I20','I21','I22','I23','I24','I25')
                then Co_IHD_nrsub+1;
```

```
                end;
end;

/* Check for Valvular disease (valv)*/
if substr(hdiax,1,3) in ('394','395','396','397','421','424','I33','I34','I35','I36','I37',
'I38','I39','I05','I06','I07','I08',',I33','I34','I35','I36','I37','I38','I39')
or substr(hdiax,1,4) in ('I091')
then do; Co_valv_nrmain+1; Co_valv_recentdatemain=indatum; Co_valv_los+los; end;
do i=1 to 30;
                if length(strip(bdia[i])) GE 3 then do;
                                if substr(upcase(strip(bdia[i])),1,3) in
('394','395','396','397','421','424','I33','I34','I35','I36','I37','I38','I39','I05','I06','I07','I08',',I33','I34','I35','I36',
                                                'I37','I38','I39')
                                then Co_valv_nrsub+1;
                end;
end;
do i=1 to 30;
                if length(strip(bdia[i])) GE 4 then do;
                                if substr(upcase(strip(bdia[i])),1,4) in ('I091')
                                then Co_valv_nrsub+1;
                end;
end;

/* Check for Cardiac arrythmias (ary) */
if substr(hdiax,1,3) in ('426','427','I44','I45','I46','I47','I48','I49','R00')
then do; Co_ary_nrmain+1; Co_ary_recentdatemain=indatum; Co_ary_los+los; end;
do i=1 to 30;
                if length(strip(bdia[i])) GE 3 then do;
                                if substr(upcase(strip(bdia[i])),1,3) in
('426','427','I44','I45','I46','I47','I48','I49','R00')
                                then Co_ary_nrsub+1;
                end;
end;

/* Check for Pulmonary circulation disorders (pulmcirc) */
if substr(hdiax,1,3) in ('415','416','417','I26','I27','I28')
then do; Co_pulmcirc_nrmain+1; Co_pulmcirc_recentdatemain=indatum; Co_pulmcirc_los+los; end;
do i=1 to 30;
                if length(strip(bdia[i])) GE 3 then do;
                                if substr(upcase(strip(bdia[i])),1,3) in ('415','416','417','I26','I27','I28')
                                then Co_pulmcirc_nrsub+1;
                end;
end;

/* Check for Hypertension (HT)*/
if substr(hdiax,1,3) in ('401','402','403','404','405','I12','I15')
or substr(hdiax,1,4) in ('I109','I119')
then do; Co_HT_nrmain+1; Co_HT_recentdatemain=indatum; Co_HT_los+los; end;
do i=1 to 30;
                if length(strip(bdia[i])) GE 3 then do;
                                if substr(upcase(strip(bdia[i])),1,3) in ('401','402','403','404','405','I12','I15')
                                then Co_HT_nrsub+1;
                end;
end;
do i=1 to 30;
                if length(strip(bdia[i])) GE 4 then do;
                                if substr(upcase(strip(bdia[i])),1,4) in ('I109','I119')
```

```
                        then Co_HT_nrsub+1;
            end;
end;

/* Check for Cerebrovascular disease (CVD) */
if substr(hdiax,1,3) in ('430','431','432','433','434','435','436','437','438',
            'I60','I61','I62','I63','I64','I65','I66','I67','I68','I69')
then do; Co_CVD_nrmain+1; Co_CVD_recentdatemain=indatum; Co_CVD_los+los; end;
do i=1 to 30;
            if length(strip(bdia[i])) GE 3 then do;
                        if substr(upcase(strip(bdia[i])),1,3) in
('430','431','432','433','434','435','436','437','438','I60','I61','I62','I63','I64','I65','I66','I67','I68','I69')
                        then Co_CVD_nrsub+1;
            end;
end;

/* Check for Peripheral vascular disease (perivasc)*/
if substr(hdiax,1,3) in ('440','441','442','443','444','445','446','447','452','453','456',
            'I70','I71','I72','I73','I74','I75',',I76','I77','I78','I79','I80','I81','I82','I85',',I86',
            'I87','I88','I89')
or substr(hdiax,1,4) in ('R029')
then do; Co_perivasc_nrmain+1; Co_perivasc_recentdatemain=indatum; Co_perivasc_los+los; end;
do i=1 to 30;
            if length(strip(bdia[i])) GE 3 then do;
                        if substr(upcase(strip(bdia[i])),1,3) in
('440','441','442','443','444','445','446','447','452','453','456','I70','I71','I72','I73','I74','I75',',I76','I77','I78','I79',
                                    'I80','I81','I82','I85',',I86','I87','I88','I89')
                        then Co_perivasc_nrsub+1;
            end;
end;
do i=1 to 30;
            if length(strip(bdia[i])) GE 4 then do;
                        if substr(upcase(strip(bdia[i])),1,4) in ('R029')
                        then Co_perivasc_nrsub+1;
            end;
end;

/* Check for Neurologic disease (perivasc)*/
if substr(hdiax,1,3) in ('290','293','294','310','R40','R41','R47','R48')
or substr(hdiax,1,3) ge '320' and substr(hdiax,1,3) le '359'
or substr(hdiax,1,3) ge 'G00' and substr(hdiax,1,3) le 'G99'
or substr(hdiax,1,3) ge 'F00' and substr(hdiax,1,3) le 'F09'
or substr(hdiax,1,3) ge 'R25' and substr(hdiax,1,3) le 'R29'
or substr(hdiax,1,4) in ('R296','R298','784D','784F','780A','781C','781D')
then do; Co_neurol_nrmain+1; Co_neurol_recentdatemain=indatum; Co_neurol_los+los; end;
do i=1 to 30;
            if length(strip(bdia[i])) GE 3 then do;
                        if substr(upcase(strip(bdia[i])),1,3) in
('290','293','294','310','R40','R41','R47','R48')
                                    or substr(upcase(strip(bdia[i])),1,3) ge '320' and
substr(upcase(strip(bdia[i])),1,3) le '359'
                                    or substr(upcase(strip(bdia[i])),1,3) ge 'G00' and
substr(upcase(strip(bdia[i])),1,3) le 'G99'
                                    or substr(upcase(strip(bdia[i])),1,3) ge 'F00' and
substr(upcase(strip(bdia[i])),1,3) le 'F09'
                                    or substr(upcase(strip(bdia[i])),1,3) ge 'R25' and
substr(upcase(strip(bdia[i])),1,3) le 'R29'
```

```
				then Co_neurol_nrsub+1;
		end;
end;
do i=1 to 30;
		if length(strip(bdia[i])) GE 4 then do;
				if substr(upcase(strip(bdia[i])),1,4) in
('R296','R298','784D','784F','780A','781C','781D')
				then Co_neurol_nrsub+1;
		end;
end;

/* Check for Chronic pulmonary disease (CPD)*/
if substr(hdiax,1,3) ge '490' and substr(hdiax,1,3) le '505'
or substr(hdiax,1,3) ge 'J40' and substr(hdiax,1,3) le 'J67'
or substr(hdiax,1,3) in ('515','516','517','J84','J98','J99')
or substr(hdiax,1,4) in ('506E','506X','508B','519B','519D','519E','519W','D860','D862',
		'E662','E840','E849','J684','J701','J703','J850','J953','J961','J969')
then do; Co_CPD_nrmain+1; Co_CPD_recentdatemain=indatum; Co_CPD_los+los; end;
do i=1 to 30;
		if length(strip(bdia[i])) GE 3 then do;
				if substr(upcase(strip(bdia[i])),1,3) ge '490' and substr(bdia[i],1,3) le '505'
						or substr(upcase(strip(bdia[i])),1,3) ge 'J40' and
substr(bdia[i],1,3) le 'J67'
						or substr(upcase(strip(bdia[i])),1,3) in
('515','516','517','J84','J98','J99')
				then Co_CPD_nrsub+1;
		end;
end;
do i=1 to 30;
		if length(strip(bdia[i])) GE 4 then do;
				if substr(upcase(strip(bdia[i])),1,4) in
('506E','506X','508B','519B','519D','519E','519W','D860','D862','E662','E840','E849','J684','J701','J703','J850','J95
3','J961','J969')
				then Co_CPD_nrsub+1;
		end;
end;

/* Check for Infectious disease (inf)*/
if substr(hdiax,1,3) in ('473','510','511','590','595','597')
or substr(hdiax,1,3) ge '001' and substr(hdiax,1,3) le '066'
or substr(hdiax,1,3) ge '071' and substr(hdiax,1,3) le '078'
or substr(hdiax,1,3) ge '080' and substr(hdiax,1,3) le '139'
or substr(hdiax,1,3) ge '460' and substr(hdiax,1,3) le '466'
or substr(hdiax,1,3) ge '480' and substr(hdiax,1,3) le '491'
or substr(hdiax,1,3) ge 'A00' and substr(hdiax,1,3) le 'B09'
or substr(hdiax,1,3) ge 'B25' and substr(hdiax,1,3) le 'B99'
or substr(hdiax,1,3) ge 'J00' and substr(hdiax,1,3) le 'J32'
or substr(hdiax,1,3) ge 'J851' and substr(hdiax,1,3) le 'J869'
or substr(hdiax,1,3) ge 'M00' and substr(hdiax,1,3) le 'M01'
or substr(hdiax,1,4) in ('070B','079A','079B','079C','079D','079W','079X','519C','B159','998F',
		'999D','M726','N300','N390','T880','790H','790W')
then do; Co_inf_nrmain+1; Co_inf_recentdatemain=indatum; Co_inf_los+los; end;
do i=1 to 30;
		if length(strip(bdia[i])) GE 3 then do;
				if substr(upcase(strip(bdia[i])),1,3) in ('473','510','511','590','595','597')
						or substr(upcase(strip(bdia[i])),1,3) ge '001' and
substr(bdia[i],1,3) le '066'
```

```
                                        or substr(upcase(strip(bdia[i])),1,3) ge '071' and
substr(bdia[i],1,3) le '078'
                                        or substr(upcase(strip(bdia[i])),1,3) ge '080' and
substr(bdia[i],1,3) le '139'
                                        or substr(upcase(strip(bdia[i])),1,3) ge '460' and
substr(bdia[i],1,3) le '466'
                                        or substr(upcase(strip(bdia[i])),1,3) ge '480' and
substr(bdia[i],1,3) le '491'
                                        or substr(upcase(strip(bdia[i])),1,3) ge 'A00' and
substr(bdia[i],1,3) le 'B09'
                                        or substr(upcase(strip(bdia[i])),1,3) ge 'B25' and
substr(bdia[i],1,3) le 'B99'
                                        or substr(upcase(strip(bdia[i])),1,3) ge 'J00' and
substr(bdia[i],1,3) le 'J32'
                                        or substr(upcase(strip(bdia[i])),1,3) ge 'J851' and
substr(bdia[i],1,3) le 'J869'
                                        or substr(upcase(strip(bdia[i])),1,3) ge 'M00' and
substr(bdia[i],1,3) le 'M01'
                        then Co_inf_nrsub+1;
            end;
end;
do i=1 to 30;
            if length(strip(bdia[i])) GE 4 then do;
                        if substr(upcase(strip(bdia[i])),1,4) in
('070B','079A','079B','079C','079D','079W','079X','519C','B159','998F','999D','M726','N300','N390','T880','790H',
'790W')
                                        or substr(upcase(strip(bdia[i])),1,3) ge 'J851' and
substr(bdia[i],1,3) le 'J869'
                        then Co_inf_nrsub+1;
            end;
end;

/* Check for Diabetes (diab) */
if substr(hdiax,1,3) in ('250','E10','E11','E12','E13','E14')
then do; Co_diab_nrmain+1; Co_diab_recentdatemain=indatum; Co_diab_los+los; end;
do i=1 to 30;
            if length(strip(bdia[i])) GE 3 then  do;
                        if substr(upcase(strip(bdia[i])),1,3) in ('250','E10','E11','E12','E13','E14')
                        then Co_diab_nrsub+1;
            end;
end;

/* Check for Other endocrine disorders (xendo) */
if substr(hdiax,1,3) in ('E89')
or substr(hdiax,1,3) ge '251' and substr(hdiax,1,3) le '259'
or substr(hdiax,1,3) ge 'E00' and substr(hdiax,1,3) le 'E07'
or substr(hdiax,1,3) ge 'E15' and substr(hdiax,1,3) le 'E35'
then do; Co_xendo_nrmain+1; Co_xendo_recentdatemain=indatum; Co_xendo_los+los; end;
do i=1 to 30;
            if length(strip(bdia[i])) GE 3 then do;
                        if substr(upcase(strip(bdia[i])),1,3) in ('E89')
                                        or substr(upcase(strip(bdia[i])),1,3) ge '251' and
substr(bdia[i],1,3) le '259'
                                        or substr(upcase(strip(bdia[i])),1,3) ge 'E00' and
substr(bdia[i],1,3) le 'E07'
                                        or substr(upcase(strip(bdia[i])),1,3) ge 'E15' and
substr(bdia[i],1,3) le 'E35'
```

```
                    then Co_xendo_nrsub+1;
          end;
end;

/* Check for Renal disease (renal) */
if substr(hdiax,1,3) in ('403','404','V56','R34')
or substr(hdiax,1,3) ge '580' and substr(hdiax,1,3) le '589'
or substr(hdiax,1,3) ge 'N00' and substr(hdiax,1,3) le 'N12'
or substr(hdiax,1,3) ge 'N14' and substr(hdiax,1,3) le 'N19'
or substr(hdiax,1,4) in ('V42A','V45B','R392','Z992')
then do; Co_renal_nrmain+1; Co_renal_recentdatemain=indatum; Co_renal_los+los; end;
do i=1 to 30;
          if length(strip(bdia[i])) GE 3 then  do;
                    if substr(upcase(strip(bdia[i])),1,3) in ('403','404','V56','R34')
                              or substr(upcase(strip(bdia[i])),1,3) ge '580' and
substr(bdia[i],1,3) le '589'
                              or substr(upcase(strip(bdia[i])),1,3) ge 'N00' and
substr(bdia[i],1,3) le 'N12'
                              or substr(upcase(strip(bdia[i])),1,3) ge 'N14' and
substr(bdia[i],1,3) le 'N19'
                    then Co_renal_nrsub+1;
          end;
end;
do i=1 to 30;
          if length(strip(bdia[i])) GE 4 then do;
                    if substr(upcase(strip(bdia[i])),1,4) in ('V42A','V45B','R392','Z992')
          then Co_renal_nrsub+1;
          end;
end;

/* Check for Hepatic disease (hepatic) */
if substr(hdiax,1,3) in ('B16','B17','B18','I85')
or substr(hdiax,1,3) ge '070C' and substr(hdiax,1,3) le '070X'
or substr(hdiax,1,3) ge '570' and substr(hdiax,1,3) le '573'
or substr(hdiax,1,3) ge 'K70' and substr(hdiax,1,3) le 'K77'
or substr(hdiax,1,4) in ('070A','456A','456B','V42H','B150')
then do; Co_hepatic_nrmain+1; Co_hepatic_recentdatemain=indatum; Co_hepatic_los+los; end;
do i=1 to 30;
          if length(strip(bdia[i])) GE 3 then  do;
                    if substr(upcase(strip(bdia[i])),1,3) in ('B16','B17','B18','I85')
                              or substr(upcase(strip(bdia[i])),1,3) ge '570' and
substr(bdia[i],1,3) le '573'
                              or substr(upcase(strip(bdia[i])),1,3) ge 'K70' and
substr(bdia[i],1,3) le 'K77'
                    then Co_hepatic_nrsub+1;
          end;
end;
do i=1 to 30;
          if length(strip(bdia[i])) GE 4 then do;
                    if substr(upcase(strip(bdia[i])),1,3) ge '070C' and substr(bdia[i],1,3) le '070X'
                              or substr(upcase(strip(bdia[i])),1,4) in
('070A','456A','456B','V42H','B150')
                    then Co_hepatic_nrsub+1;
          end;
end;

/* Check for Immune deficiencies incl. HIV (immundef) */
```

```
if substr(hdiax,1,3) in ('279','173','D89')
or substr(hdiax,1,3) ge 'B20' and substr(hdiax,1,3) le 'B24'
or substr(hdiax,1,3) ge 'D80' and substr(hdiax,1,3) le 'D84'
or substr(hdiax,1,4) in ('079J','V02J')
then do; Co_immundef_nrmain+1; Co_immundef_recentdatemain=indatum; Co_immundef_los+los; end;
do i=1 to 30;
        if length(strip(bdia[i])) GE 3 then do;
                if substr(upcase(strip(bdia[i])),1,3) in ('279','173','D89')
                        or substr(upcase(strip(bdia[i])),1,3) ge 'B20' and
substr(bdia[i],1,3) le 'B24'
                        or substr(upcase(strip(bdia[i])),1,3) ge 'D80' and
substr(bdia[i],1,3) le 'D84'
                then Co_immundef_nrsub+1;
        end;
end;
do i=1 to 30;
        if length(strip(bdia[i])) GE 4 then do;
                if substr(upcase(strip(bdia[i])),1,4) in ('079J','V02J')
                then Co_immundef_nrsub+1;
        end;
end;

/* Check for Hematological malignancies (hemomal) */
if substr(hdiax,1,3) ge '200' and substr(hdiax,1,3) le '208'
or substr(hdiax,1,3) ge 'C81' and substr(hdiax,1,3) le 'C96'
or substr(hdiax,1,4) in ('D477','D478','D479')
then do; Co_hemomal_nrmain+1; Co_hemomal_recentdatemain=indatum; Co_hemomal_los+los; end;
do i=1 to 30;
        if length(strip(bdia[i])) GE 3 then do;
                if substr(upcase(strip(bdia[i])),1,3) ge '200' and substr(bdia[i],1,3) le '208'
                        or substr(upcase(strip(bdia[i])),1,3) ge 'C81' and
substr(bdia[i],1,3) le 'C96'
                then Co_hemomal_nrsub+1;
        end;
end;
do i=1 to 30;
        if length(strip(bdia[i])) GE 4 then do;
                if substr(upcase(strip(bdia[i])),1,4) in ('D477','D478','D479')
                then Co_hemomal_nrsub+1;
        end;
end;

/* Check for Other hematological disease (hemodis) */
if substr(hdiax,1,3) in ('288','289','D45','D46')
or substr(hdiax,1,3) ge 'D69' and substr(hdiax,1,3) le 'D77'
or substr(hdiax,1,4) in ('D471','D472','D473')
then do; Co_hemodis_nrmain+1; Co_hemodis_recentdatemain=indatum; Co_hemodis_los+los; end;
do i=1 to 30;
        if length(strip(bdia[i])) GE 3 then do;
                if substr(upcase(strip(bdia[i])),1,3) in ('288','289','D45','D46')
                        or substr(upcase(strip(bdia[i])),1,3) ge 'D69' and
substr(bdia[i],1,3) le 'D77'
                then Co_hemodis_nrsub+1;
        end;
end;
do i=1 to 30;
        if length(strip(bdia[i])) GE 4 then do;
```

```
                    if substr(upcase(strip(bdia[i])),1,4) in ('456A','456B','V42H')
                    then Co_hemodis_nrsub+1;
          end;
end;

/* Check for Solid tumour without metastases (tumournomet) */
if substr(hdiax,1,3) ge '140' and substr(hdiax,1,3) le '195'
or substr(hdiax,1,3) ge 'C00' and substr(hdiax,1,3) le 'C76'
or substr(hdiax,1,4) in ('Z510','Z511','Z926')
then do; Co_tumournomet_nrmain+1; Co_tumournomet_recentdatemain=indatum;
Co_tumournomet_los+los; end;
do i=1 to 30;
          if length(strip(bdia[i])) GE 3 then do;
                    if substr(upcase(strip(bdia[i])),1,3) ge '140' and substr(bdia[i],1,3) le '195'
                              or substr(upcase(strip(bdia[i])),1,3) ge 'C00' and
substr(bdia[i],1,3) le 'C76'
                    then Co_tumournomet_nrsub+1;
          end;
end;
do i=1 to 30;
          if length(strip(bdia[i])) GE 4 then do;
                    if substr(upcase(strip(bdia[i])),1,4) in ('Z510','Z511','Z926')
                    then Co_tumournomet_nrsub+1;
          end;
end;

/* Check for Metastatic cancer (tumourmet) */
if substr(hdiax,1,3) ge '196' and substr(hdiax,1,3) le '199'
or substr(hdiax,1,3) ge 'C77' and substr(hdiax,1,3) le 'C80'
or substr(hdiax,1,4) in ('C979')
then do; Co_tumourmet_nrmain+1; Co_tumourmet_recentdatemain=indatum; Co_tumourmet_los+los; end;
do i=1 to 30;
          if length(strip(bdia[i])) GE 3 then do;
                    if substr(upcase(strip(bdia[i])),1,3) ge '196' and substr(bdia[i],1,3) le '199'
                              or substr(upcase(strip(bdia[i])),1,3) ge 'C77' and
substr(bdia[i],1,3) le 'C80'
                    then Co_tumourmet_nrsub+1;
          end;
end;
do i=1 to 30;
          if length(strip(bdia[i])) GE 4 then do;
                    if substr(upcase(strip(bdia[i])),1,4) in ('C979')
                    then Co_tumourmet_nrsub+1;
          end;
end;

/* Check for Rheumatic disease (collagen) */
if substr(hdiax,1,3) in ('710','714','720','725')
or substr(hdiax,1,3) ge '729A' and substr(hdiax,1,3) le '729E'
or substr(hdiax,1,3) ge 'M05' and substr(hdiax,1,3) le 'M09'
or substr(hdiax,1,3) ge 'M30' and substr(hdiax,1,3) le 'M35'
or substr(hdiax,1,4) in ('279N','713D','713H')
then do; Co_collagen_nrmain+1; Co_collagen_recentdatemain=indatum; Co_collagen_los+los; end;
do i=1 to 30;
          if length(strip(bdia[i])) GE 3 then do;
                    if substr(upcase(strip(bdia[i])),1,3) in ('710','714','720','725')
```

```
                                        or substr(upcase(strip(bdia[i])),1,3) ge '729A' and
substr(bdia[i],1,3) le '729E'
                                        or substr(upcase(strip(bdia[i])),1,3) ge 'M05' and
substr(bdia[i],1,3) le 'M09'
                                        or substr(upcase(strip(bdia[i])),1,3) ge 'M30' and
substr(bdia[i],1,3) le 'M35'
                        then Co_collagen_nrsub+1;
            end;
end;
do i=1 to 30;
            if length(strip(bdia[i])) GE 4 then do;
                        if substr(upcase(strip(bdia[i])),1,4) in ('279N','713D','713H')
                        then Co_collagen_nrsub+1;
            end;
end;

/* Check for Coagulopathy (coag) */
if substr(hdiax,1,3) in ('286','287','D65','D66','D67','D68')
then do; Co_coag_nrmain+1; Co_coag_recentdatemain=indatum; Co_coag_los+los; end;
do i=1 to 30;
            if length(strip(bdia[i])) GE 3 then do;
                        if substr(upcase(strip(bdia[i])),1,3) in ('286','287','D65','D66','D67','D68')
                        then Co_coag_nrsub+1;
            end;
end;

/* Check for Obesity (obese) */
if substr(hdiax,1,3) in ('278','E65','E66')
then do; Co_obese_nrmain+1; Co_obese_recentdatemain=indatum; Co_obese_los+los; end;
do i=1 to 30;
            if length(strip(bdia[i])) GE 3 then do;
                        if substr(upcase(strip(bdia[i])),1,3) in ('278','E65','E66')
                        then Co_obese_nrsub+1;
            end;
end;

/* Check for Nutritional deficiences (nutr) */
if substr(hdiax,1,3) in ('E90')
or substr(hdiax,1,3) ge '260' and substr(hdiax,1,3) le '269'
or substr(hdiax,1,3) ge 'E40' and substr(hdiax,1,3) le 'E64'
then do; Co_nutr_nrmain+1; Co_nutr_recentdatemain=indatum; Co_nutr_los+los; end;
do i=1 to 30;
            if length(strip(bdia[i])) GE 3 then do;
                        if substr(upcase(strip(bdia[i])),1,3) in ('E90')
                                    or substr(upcase(strip(bdia[i])),1,3) ge '260' and
substr(bdia[i],1,3) le '269'
                                    or substr(upcase(strip(bdia[i])),1,3) ge 'E40' and
substr(bdia[i],1,3) le 'E64'
                        then Co_nutr_nrsub+1;
            end;
end;

/* Check for Fluid and electrolytes disorders (fluid) */
if substr(hdiax,1,3) in ('276','E86','E87')
or substr(hdiax,1,4) in ('R631')
then do; Co_fluid_nrmain+1; Co_fluid_recentdatemain=indatum; Co_fluid_los+los; end;
do i=1 to 30;
```

```
            if length(strip(bdia[i])) GE 3 then do;
                        if substr(upcase(strip(bdia[i])),1,3) in ('276','E86','E87')
                        then Co_fluid_nrsub+1;
            end;
end;
do i=1 to 30;
            if length(strip(bdia[i])) GE 4 then do;
                        if substr(upcase(strip(bdia[i])),1,4) in ('R631')
                        then Co_fluid_nrsub+1;
            end;
end;

/* Check for Blood loss anemia (bleed) */
if substr(hdiax,1,4) in ('285B','D629')
then do; Co_bleed_nrmain+1; Co_bleed_recentdatemain=indatum; Co_bleed_los+los; end;
do i=1 to 30;
            if length(strip(bdia[i])) GE 4 then do;
                        if substr(upcase(strip(bdia[i])),1,4) in ('285B','D629')
                        then Co_bleed_nrsub+1;
            end;
end;

/* Check for Deficiency and other anemias (anemia) */
if substr(hdiax,1,3) in ('280','281','282','283','284','D64')
or substr(hdiax,1,4) in ('285A','285W','285X')
or substr(hdiax,1,3) ge 'D50' and substr(hdiax,1,3) le 'D61'
then do; Co_anemia_nrmain+1; Co_anemia_recentdatemain=indatum; Co_anemia_los+los; end;
do i=1 to 30;
            if length(strip(bdia[i])) GE 3 then do;
                        if substr(upcase(strip(bdia[i])),1,3) in ('280','281','282','283','284','D64')
                                    or substr(upcase(strip(bdia[i])),1,3) ge 'D50' and
substr(bdia[i],1,3) le 'D61'
                        then Co_anemia_nrsub+1;
            end;
end;
do i=1 to 30;
            if length(strip(bdia[i])) GE 4 then do;
                        if substr(upcase(strip(bdia[i])),1,4) in ('285A','285W','285X')
                        then Co_anemia_nrsub+1;
            end;
end;

/* Check for Alcohol abuse (alco) */
if substr(hdiax,1,3) in ('291','303','F10','K70','Y90','Y91')
or substr(hdiax,1,4) in ('305A','K852','R780','Z502','Z721','790D')
then do; Co_alco_nrmain+1; Co_alco_recentdatemain=indatum; Co_alco_los+los; end;
do i=1 to 30;
            if length(strip(bdia[i])) GE 3 then do;
                        if substr(upcase(strip(bdia[i])),1,3) in ('291','303','F10','K70','Y90','Y91')
                        then Co_alco_nrsub+1;
            end;
end;
do i=1 to 30;
            if length(strip(bdia[i])) GE 4 then do;
                        if substr(upcase(strip(bdia[i])),1,4) in ('305A','K852','R780','Z502','Z721','790D')
                        then Co_alco_nrsub+1;
            end;
```

```
end;

/* Check for Drug abuse (drug) */
if substr(hdiax,1,3) in ('292','304')
or substr(hdiax,1,3) ge 'F11' and substr(hdiax,1,3) le 'F19'
or substr(hdiax,1,4) in ('305B','305X','R781','R782','R783','R784','R785','R786','Z503','Z722')
then do; Co_drug_nrmain+1; Co_drug_recentdatemain=indatum; Co_drug_los+los; end;
do i=1 to 30;
		if length(strip(bdia[i])) GE 3 then do;
				if substr(upcase(strip(bdia[i])),1,3) in ('292','304')
						or substr(upcase(strip(bdia[i])),1,3) ge 'F11' and
substr(bdia[i],1,3) le 'F19'
				then Co_drug_nrsub+1;
		end;
end;
do i=1 to 30;
		if length(strip(bdia[i])) GE 4 then do;
				if substr(upcase(strip(bdia[i])),1,4) in
('305B','305X','R781','R782','R783','R784','R785','R786','Z503','Z722')
				then Co_drug_nrsub+1;
		end;
end;

/* Check for Psychoses (psycho) */
if substr(hdiax,1,3) in ('295','297','298','299','R44')
or substr(hdiax,1,3) ge 'F20' and substr(hdiax,1,3) le 'F29'
or substr(hdiax,1,4) in ('780B')
then do; Co_psycho_nrmain+1; Co_psycho_recentdatemain=indatum; Co_psycho_los+los; end;
do i=1 to 30;
		if length(strip(bdia[i])) GE 3 then do;
				if substr(upcase(strip(bdia[i])),1,3) in ('295','297','298','299','R44')
						or substr(upcase(strip(bdia[i])),1,3) ge 'F20' and
substr(bdia[i],1,3) le 'F29'
				then Co_psycho_nrsub+1;
		end;
end;
do i=1 to 30;
		if length(strip(bdia[i])) GE 4 then do;
				if substr(upcase(strip(bdia[i])),1,4) in ('780B')
				then Co_psycho_nrsub+1;
		end;
end;

/* Check for affective disorders (affect) */
if substr(hdiax,1,3) in ('296','311')
or substr(hdiax,1,3) ge 'F30' and substr(hdiax,1,3) le 'F39'
then do; Co_affect_nrmain+1; Co_affect_recentdatemain=indatum; Co_affect_los+los; end;
do i=1 to 30;
		if length(strip(bdia[i])) GE 3 then do;
				if substr(upcase(strip(bdia[i])),1,3) in ('296','311')
						or substr(upcase(strip(bdia[i])),1,3) ge 'F30' and
substr(bdia[i],1,3) le 'F39'
				then Co_affect_nrsub+1;
		end;
end;

/* Check for Other psychiatric disorders (xpsych) */
```

```
if substr(hdiax,1,3) in ('300','301','302','307','308','309')
or substr(hdiax,1,3) ge '312' and substr(hdiax,1,3) le '319'
or substr(hdiax,1,3) ge 'F40' and substr(hdiax,1,3) le 'F99'
or substr(hdiax,1,4) in ('Z504')
then do; Co_xpsych_nrmain+1; Co_xpsych_recentdatemain=indatum; Co_xpsych_los+los; end;
do i=1 to 30;
		if length(strip(bdia[i])) GE 3 then do;
				if substr(upcase(strip(bdia[i])),1,3) in ('300','301','302','307','308','309')
						or substr(upcase(strip(bdia[i])),1,3) ge '312' and
substr(bdia[i],1,3) le '319'
						or substr(upcase(strip(bdia[i])),1,3) ge 'F40' and
substr(bdia[i],1,3) le 'F99'
				then Co_xpsych_nrsub+1;
		end;
end;
do i=1 to 30;
		if length(strip(bdia[i])) GE 4 then do;
				if substr(upcase(strip(bdia[i])),1,4) in ('Z504')
				then Co_xpsych_nrsub+1;
		end;
end;

/* Check for Transplantation (tx) */
if substr(hdiax,1,3) in ('V42','T86')
or substr(hdiax,1,4) in ('996W','Z940','Z941','Z942','Z943','Z944')
then do; Co_tx_nrmain+1; Co_tx_recentdatemain=indatum; Co_tx_los+los; end;
do i=1 to 30;
		if length(strip(bdia[i])) GE 3 then do;
				if substr(upcase(strip(bdia[i])),1,3) in ('V42','T86')
				then Co_tx_nrsub+1;
		end;
end;
do i=1 to 30;
		if length(strip(bdia[i])) GE 4 then do;
				if substr(upcase(strip(bdia[i])),1,4) in ('996W','Z940','Z941','Z942','Z943','Z944')
				then Co_tx_nrsub+1;
		end;
end;

/* Check for Bone or muscle disease (bonemusc) */
if substr(hdiax,1,3) in ('M86','M87','M88','M89','M90','M99')
or substr(hdiax,1,3) ge '715' and substr(hdiax,1,3) le '738'
or substr(hdiax,1,3) ge 'M12' and substr(hdiax,1,3) le 'M25'
or substr(hdiax,1,3) ge 'M40' and substr(hdiax,1,3) le 'M83'
or substr(hdiax,1,4) in ('M858','M859')
then do; Co_bonemusc_nrmain+1; Co_bonemusc_recentdatemain=indatum; Co_bonemusc_los+los; end;
do i=1 to 30;
		if length(strip(bdia[i])) GE 3 then do;
				if substr(upcase(strip(bdia[i])),1,3) in ('M86','M87','M88','M89','M90','M99')
						or substr(upcase(strip(bdia[i])),1,3) ge '715' and
substr(bdia[i],1,3) le '738'
						or substr(upcase(strip(bdia[i])),1,3) ge 'M12' and
substr(bdia[i],1,3) le 'M25'
						or substr(upcase(strip(bdia[i])),1,3) ge 'M40' and
substr(bdia[i],1,3) le 'M83'
				then Co_bonemusc_nrsub+1;
		end;
```

```
end;
do i=1 to 30;
			if length(strip(bdia[i])) GE 4 then do;
						if substr(upcase(strip(bdia[i])),1,4) in ('M858','M859')
						then Co_bonemusc_nrsub+1;
			end;
end;

/* Check for Injury (injury) */
if substr(hdiax,1,3) ge '800' and substr(hdiax,1,3) le '959'
or substr(hdiax,1,3) ge '990' and substr(hdiax,1,3) le '994'
or substr(hdiax,1,3) ge 'S00' and substr(hdiax,1,3) le 'T35'
or substr(hdiax,1,3) ge 'T66' and substr(hdiax,1,3) le 'T79'
or substr(hdiax,1,3) ge 'T90' and substr(hdiax,1,3) le 'T98'
or substr(hdiax,1,4) in ('995F')
then do; Co_injury_nrmain+1; Co_injury_recentdatemain=indatum; Co_injury_los+los; end;
do i=1 to 30;
			if length(strip(bdia[i])) GE 3 then do;
						if substr(upcase(strip(bdia[i])),1,3) ge '800' and substr(bdia[i],1,3) le '959'
									or substr(upcase(strip(bdia[i])),1,3) ge '990' and
substr(bdia[i],1,3) le '994'
									or substr(upcase(strip(bdia[i])),1,3) ge 'S00' and
substr(bdia[i],1,3) le 'T35'
									or substr(upcase(strip(bdia[i])),1,3) ge 'T66' and
substr(bdia[i],1,3) le 'T79'
									or substr(upcase(strip(bdia[i])),1,3) ge 'T90' and
substr(bdia[i],1,3) le 'T98'
						then Co_injury_nrsub+1;
			end;
end;
do i=1 to 30;
			if length(strip(bdia[i])) GE 4 then do;
						if substr(upcase(strip(bdia[i])),1,4) in ('995F')
						then Co_injury_nrsub+1;
			end;
end;

/* Check for Intoxication (intox) */
if substr(hdiax,1,3) ge '960' and substr(hdiax,1,3) le '989'
or substr(hdiax,1,3) ge 'T36' and substr(hdiax,1,3) le 'T65'
then do; Co_intox_nrmain+1; Co_intox_recentdatemain=indatum; Co_intox_los+los; end;
do i=1 to 30;
			if length(strip(bdia[i])) GE 3 then do;
						if substr(upcase(strip(bdia[i])),1,3) ge '960' and substr(bdia[i],1,3) le '989'
									or substr(upcase(strip(bdia[i])),1,3) ge 'T36' and
substr(bdia[i],1,3) le 'T65'
						then Co_intox_nrsub+1;
			end;
end;

												end;
						end;
						when (%sysrc(_dsenom)) do; /* Ingen tr‰ff. Vill inte att det betyder error */
									_error_=0;
						end;
									otherwise;
						end;
```

```
		end;
				if lopnr='' then
						lopnr = sparid;
				else
						klar = 1;
		end;
		output;
		sparid = lopnr;
		drop indatum hdiax dia1-dia30 los klar i sparid;
run;

/* Check result of comorbidity extraction */
proc freq data = data.injury9819_co; table Co_injury_nrmain Co_injury_nrsub; run;
proc means data = data.injury9819_co; var Co_injury_recentdatemain Co_injury_los; run;


/*****************************************************************************************
***
Program:	Select incident injuries
Purpose:	Use prediction model to select incident (index) injury admissions
Created:	2007-03-26 by Rolf Gedeborg
Modified:	2021-08-22 by Rolf Gedeborg
Notes:			Should also include prehospital deaths

*****************************************************************************************
***/

options fmtsearch=(formats);

/*		CHAPTER 1: Create indicator variables in original dataset to use in algorithm for detection of
		incident admissions
----------------------------------------------------------------------------------------------*/
data d20; set data.injury9819_co;

/* Set default new variables */
intox=0; rehab=0; same_dx=0; same_dx2=0; burn=0; susp_readm=0; adm_intval=.; commotio=0;

/* Calculate the interval between the previous injury discharge and the current injury admission.
		Note that for the first year this interval is calculated from the previous injury ADMISSION
		date. The variable adm_interval is created */
if prev_inj_disdate NE . then adm_intval = indate1 - prev_inj_disdate;
if prev_inj_disdate = . AND Co_injury_recentdatemain NE . then adm_intval = indate1-
Co_injury_recentdatemain;

sqr_adm_intval = adm_intval**2;

/* Create flag variable for SAME DX using 3 positions and groups of 4 positions for S72 */
format dxcompare1 dxcompare2 $4.;
if first.lopnr then do;
		dxcompare1 = '';
		dxcompare2 = '';
		end;
by lopnr; retain dxcompare1 dxcompare2;
if first.lopnr then do;
		dxcompare1 = '';
		dxcompare2 = '';
		end;
```

```
if dx1 NE '' then do;
if dxcompare2 in('S720','S721','S722','820') AND (substr(upcase(strip(dx1)),1,4) in('S720','S721','S722') OR
substr(upcase(strip(dx1)),1,3) in('820'))
            then same_dx = 1;
if adm_intval NE . AND dxcompare2 in('S720','S721','S722','820') AND substr(upcase(strip(dx1)),1,4) NOT
in('S720','S721','S722') AND
            substr(upcase(strip(dx1)),1,3) NOT in('820')
            then same_dx = 0;

if dxcompare2 in('S723','S724','S725','S726','S727','S728','S729','821') AND
            substr(upcase(strip(dx1)),1,4) in('S723','S724','S725','S726','S727','S728','S729') OR
substr(upcase(strip(dx1)),1,3) in('821')
            then same_dx = 1;
if adm_intval NE . AND dxcompare2 in('S723','S724','S725','S726','S727','S728','S729','821') AND
            substr(upcase(strip(dx1)),1,4) NOT in('S723','S724','S725','S726','S727','S728','S729') AND
substr(upcase(strip(dx1)),1,3) NOT in('821')
            then same_dx = 0;

if dxcompare2 NOT in('S720','S721','S722','S723','S724','S725','S726','S727','S728','S729','821')
            AND dxcompare1 = substr(upcase(strip(dx1)),1,3)
            then same_dx = 1;
if adm_intval NE . AND dxcompare2 NOT
in('S720','S721','S722','S723','S724','S725','S726','S727','S728','S729','821')
            AND dxcompare1 NE substr(upcase(strip(dx1)),1,3)
            then same_dx = 0;

/* Create flag variable for SAME DX using 4 positions */
if dxcompare2 = substr(upcase(strip(dx1)),1,4) then same_dx2 = 1; else same_dx2 = 0;

dxcompare1 = substr(upcase(strip(dx1)),1,3); dxcompare2 = substr(upcase(strip(dx1)),1,4);
DROP dxcompare1 dxcompare2;

/* Create flag variables for poisoning, rehab care, burn and commotio */
if substr(upcase(strip(dx1)),1,3) GE 'T36' AND substr(upcase(strip(dx1)),1,3) LE 'T65'
            then intox = 1;
if substr(upcase(strip(dx1)),1,3) in('T96','T97')
            then intox = 1;
if strip(lt_klin1) in('76','076','86','086','90','090','96','096','98','098','121','240','241','242','243','244','246','251',
            '551','552','561','562','563','564','565')
            then rehab = 1;
if substr(upcase(strip(dx1)),1,3) GE 'T20' AND substr(strip(dx1),1,3) LE 'T35'
            then burn = 1;
if substr(upcase(strip(dx1)),1,4) EQ 'S060'
            then commotio = 1;

            end;

if pvard = 2 then plan_care = 0; if pvard = 1 then plan_care = 1;

age = intck('DAY', bdate, indate1) / 365.25;
if age = . then age = age_d;

year = year(indate1);
run;

/* Check result of previous step - components of predictors for readmissions by year */
```

```
proc freq data = d20; table plan_care*year same_dx*year same_dx2*year intox*year rehab*year burn*year
commotio*year / missing plots = freqplot;
title 'Check - distribution components of predictors for readmissions by year'; run;
proc sort data = d20; by year; run;
proc means data = d20; by year; var adm_intval; run;

/* Chapter 2 - apply prediction model for incident injuries on the original dataset */
title 'Apply model to original dataset';
proc logistic INMODEL = data.outm_incid_inj2 (type = logismod);
                    score data = d20 out = d20b; run;

proc means data = d20b; var P_0; run;

/* Mark predicted readmissions and delete them from dataset*/
data data.injury9819_incid; set d20b;
                    if P_0>0.55319 then susp_readm = 0; else susp_readm = 1;                    *Cutoff for
prediction;
                    if adm_intval = . then susp_readm = 0;
                                                                  *No previous admission - considered incident;
                    if intox = 1 then susp_readm = 0;
                                                                                *All poisoning considered
incident;
                    if adm_status1 = '4' then susp_readm = 0;
                                                    *All prehospital deaths considered incident;
                    if adm_intval GE 2*365.25 then susp_readm = 0;
                                                    *All admissions more than 2 years apart set to incident;

DROP intox rehab same_dx same_dx2 burn commotio sqr_adm_intval I_readm P_0 P_1;
run;

proc freq data = data.injury9819_incid; table susp_readm*year / missing plots = freqplot; run;

/* Create new variable that indicates next incident injury admission date (next_indexadm_date) */
/*proc sort data = data.injury9819_incid; by lopnr descending inj_adm_no; run; /*Sort each patients injury
admissions descending order */
/*
data data.injury9819_incid; set data.injury9819_incid;
if susp_readm = 0;

format tempdate1 next_indexadm_date yymmdd10.;
if first.lopnr then tempdate1 = .;
by lopnr; retain tempdate1;
if first.lopnr then tempdate1 = .;
next_indexadm_date = tempdate1; tempdate1 = indate1;
drop tempdate1; run; */


/* Extract information on comorbidity from Swedish National Discharge Register (all admissions
5 years prior to injury admission). Conditions are defined both according to ICD-9 and
ICD-10. The program is to define Charlson comorbidities based on ICD-9 and ICD-10 diagnosis codes that are
found during
the 5-year period before the present hospital separation.

Reference: Quan et al. :Coding algorithms for defining comorbidities in ICD-9-CM and ICD-10 administrative
data.
Med Care 2005 Nov; 43(11):1073-1077.
```

Aug 2021 updated in accordance with Ludvigsson et al.

ICD-9 definitions have been modified to detect the Swedish version that uses letters instead of numbers in the fourth
position of the code.

Create local copy of file with previous hospital admissions. Keep variables xupin(personnummer) adm_date(inskrivningsdatum), los(vÂrdtid), main_diag(huvuddiagnos) och bdia1-bdia7(bidiagnoser). */

```
/*data data.trauma_comorb1x; set data.trauma_comorb1x
        (keep=lopnr indatum los main_diag bdia1-bdia7); run; */

/* Create index on lopnr(personnummer) */
/*proc sql;
        create index lopnr
        on tempdata.trauma_comorb1x;
quit; */

/* Perform advanced merge to create new variables for comorbidity*/
data data.store9819_comorb_Ch_prev5yr; set data.injury9819_incid;
retain sparid;
        if lopnr = sparid then do;
                lopnr = '';
        end;

format Ch_prev5y_MI Ch_prev5y_CHF Ch_prev5y_PVD Ch_prev5y_CEVD Ch_prev5y_DEM Ch_prev5y_COPD
                Ch_prev5y_Rheum Ch_prev5y_PUD Ch_prev5y_MILDLD Ch_prev5y_DIAB_UC
Ch_prev5y_DIAB_C Ch_prev5y_PARA Ch_prev5y_RD
                Ch_prev5y_CANCER Ch_prev5y_MSLD Ch_prev5y_METS Ch_prev5y_HIV 2.;

/* set counters to zero */
array Ch_var[*] Ch_prev5y_MI Ch_prev5y_CHF Ch_prev5y_PVD Ch_prev5y_CEVD Ch_prev5y_DEM
Ch_prev5y_COPD
                Ch_prev5y_Rheum Ch_prev5y_PUD Ch_prev5y_MILDLD Ch_prev5y_DIAB_UC
Ch_prev5y_DIAB_C Ch_prev5y_PARA Ch_prev5y_RD
                Ch_prev5y_CANCER Ch_prev5y_MSLD Ch_prev5y_METS Ch_prev5y_HIV;

do i = 1 to 17;
        Ch_var[i] = 0;
end;

array dia[*] $ hdia dia1-dia30; /* Define array for diagnoses */

CALL MISSING (hdia, of dia1-dia30, indatum, los); /* Set variables in lookup table to missing */

        do until(klar = 1);
          /* Extra loop som tvingar fram l‰sning med id=missing om */
                    /* samma nyckel finns flera ggr i taba=grundtabellen (h‰r


        "datasafe.injury9804") */
        do until(_iorc_=%sysrc(_dsenom)); /* Villkor som checkar om tr‰ff vid sˆkning */
                set trauma_comorb1 key = lopnr;
                select(_iorc_);
                when (%sysrc(_sok)) do;   /* Tr‰ff vid sˆkning. H‰r kan den egna koden
l‰ggas in */
```

```
                                        if indatum lt indate1 and /*
Define interval for extraction of information */
                                          indatum ge indate1-(365*5)
                                        then do;

/* L‰gg in det som ska gˆras i varje loop h‰r! */
/**Myocardial Infarction**/
do i=1 to 31;
        if length(strip(dia[i])) GE 3 then do;
                if substr(upcase(strip(dia[i])),1,3) in ('I21','I22')
                        or substr(upcase(strip(dia[i])),1,3) in ('410','412')
                then Ch_prev5y_MI = 1;
        end;
        if length(strip(dia[i])) GE 4 then do;
                if substr(upcase(strip(dia[i])),1,4) in ('I252')
                then Ch_prev5y_MI = 1;
        end;
end;

/**Congestive Heart Failure (Removed according to Ludvigson 2021: '398X','I099','I425','P290')**/
do i=1 to 31;
        if length(strip(dia[i])) GE 3 then do;
                if substr(upcase(strip(dia[i])),1,3) in ('I43','I50')
                        or substr(upcase(strip(dia[i])),1,3) in ('428')
                then Ch_prev5y_CHF = 1;
        end;
        if length(strip(dia[i])) GE 4 then do;
                if substr(upcase(strip(dia[i])),1,4) in ('I110','I130','I132',
'I255','I420','I426','I427','I428','I429')
                        or substr(upcase(strip(dia[i])),1,4) in
('402A','402B','402X','404A','404B','404X','425E','425F','425H','425W','425X')
                then Ch_prev5y_CHF = 1;
        end;
end;

/**Peripheral Vascular Disease (Removed according to Ludvigson 2021:
'093A','437D','443C','443W','557B','5579','V43E','K551','K558','K559','Z958','Z959')**/
do i=1 to 31;
        if length(strip(dia[i])) GE 3 then do;
                if substr(upcase(strip(dia[i])),1,3) in ('I70','I71','K55')
                        or substr(upcase(strip(dia[i])),1,3) in ('440','441','557')
                then Ch_prev5y_PVD = 1;
        end;
        if length(strip(dia[i])) GE 4 then do;
                if substr(upcase(strip(dia[i])),1,4) in ('I731','I738','I739','I771','I790','I792')
                        or substr(upcase(strip(dia[i])),1,4) in ('443B','443X','447B')
                then Ch_prev5y_PVD = 1;
        end;
end;

/**Cerebrovascular Disease (Removed according to Ludvigson 2021: '362D','G46','H340','I65','I66','I68') **/
do i=1 to 31;
        if length(strip(dia[i])) GE 3 then do;
                if substr(upcase(strip(dia[i])),1,3) in ('G45','I60','I61','I62','I63','I64','I67','I69')
                        or substr(upcase(strip(dia[i])),1,3) in
('430','431','432','433','434','435','436','437','438')
                then Ch_prev5y_CEVD = 1;
```

```
                end;
end;

/**Chronic Pulmonary Disease (Removed according to Ludvigson 2021:
'491','492','496','J40','J43','J44','I278','I279','J684','J701','J703','416W','416X','506E','508B','508W') **/
do i=1 to 31;
                if length(strip(dia[i])) GE 3 then do;
                                if substr(upcase(strip(dia[i])),1,3) in
('J41','J42','J45','J46','J47','J60','J61','J62','J63','J64','J65','J66','J67','J68','J69','J70')
                                                or substr(upcase(strip(dia[i])),1,3) in
('490','493','494','495','500','501','502','503','504','505','506','507','508','516','517')
                                then Ch_prev5y_COPD = 1;
                end;
end;

/**Connective Tissue Disease-Rheumatic Disease (Removed according to Ludvigson 2021:
'446F','714A','714B','714C','714W','M360') **/
do i=1 to 31;
                if length(strip(dia[i])) GE 3 then do;
                                if substr(upcase(strip(dia[i])),1,3) in
('M05','M06','M08','M13','M30','M32','M33','M34','M45','M46')
                                                or substr(upcase(strip(dia[i])),1,3) in ('446','714','720','725')
                                then Ch_prev5y_Rheum = 1;
                end;
                if length(strip(dia[i])) GE 4 then do;
                                if substr(upcase(strip(dia[i])),1,4) in
('M070','M071','M072','M073','M123','M313','M314','M315','M316','M350','M351','M353')
                                                or substr(upcase(strip(dia[i])),1,4) in
('710A','710B','710C','710D','710E','719D')
                                then Ch_prev5y_Rheum = 1;
                end;
end;

/**Dementia (Removed according to Ludvigson 2021: ) **/
do i=1 to 31;
                if length(strip(dia[i])) GE 3 then do;
                                if substr(upcase(strip(dia[i])),1,3) in ('F00','F01','F02','F03','G30')
                                                or substr(upcase(strip(dia[i])),1,3) in ('290')
                                then Ch_prev5y_DEM = 1;
                end;
                if length(strip(dia[i])) GE 4 then do;
                                if substr(upcase(strip(dia[i])),1,4) in ('F051','G311','G319')
                                                or substr(upcase(strip(dia[i])),1,4) in
('294B','331A','331B','331C','331X')
                                then Ch_prev5y_DEM = 1;
                end;
end;

/**Paraplegia and Hemiplegia (Removed according to Ludvigson 2021:
'334B','344G','344X','G041','G801','G802','G834') **/
do i=1 to 31;
                if length(strip(dia[i])) GE 3 then do;
                                if substr(upcase(strip(dia[i])),1,3) in ('G80','G81','G82')
                                                or substr(upcase(strip(dia[i])),1,3) in ('342','343')
                                then Ch_prev5y_PARA = 1;
                end;
                if length(strip(dia[i])) GE 4 then do;
```

```
                    if substr(upcase(strip(dia[i])),1,4) in ('G114','G830','G831','G832','G833','G838')
                              or substr(upcase(strip(dia[i])),1,4) in
('344A','344B','344C','344D','344E','344F')
                    then Ch_prev5y_PARA = 1;
          end;
end;

/**Diabetes without complications (Removed according to Ludvigson 2021:
'250D','250W','250X','E106','E108','E109','E116','E118','E119','E126','E128','E129','E136',
'E138','E139','E146','E148','E149') **/
do i=1 to 31;
          if length(strip(dia[i])) GE 4 then do;
                    if substr(upcase(strip(dia[i])),1,4) in
('E100','E101','E110','E111','E120','E121','E130','E131','E140','E141')
                              or substr(upcase(strip(dia[i])),1,4) in ('250A','250B','250C')
                    then Ch_prev5y_DIAB_UC = 1;
          end;
end;

/** Diabetes with complications (Removed according to Ludvigson 2021: '250H') **/
do i=1 to 31;
          if length(strip(dia[i])) GE 4 then do;
                    if substr(upcase(strip(dia[i])),1,4) in
('E102','E103','E104','E105','E106','E107','E112','E113','E114','E115','E116','E117','E122','E123','E124',

          'E125','E126','E127','E132','E133','E134','E135','E136','E137','E142','E143','E144','E145','E146','E
147')
                              or substr(upcase(strip(dia[i])),1,4) in
('250D','250E','250F','250G')
                    then Ch_prev5y_DIAB_C = 1;
          end;
end;

/** Renal Disease (Removed according to Ludvigson 2021:
'404A','404B','404X','583A','583B','583C','583E','583G','583H','588A','Z490','Z491','Z492') **/
do i=1 to 31;
          if length(strip(dia[i])) GE 3 then do;
                    if substr(upcase(strip(dia[i])),1,3) in ('N11','N18','N19','Z49')
                              or substr(upcase(strip(dia[i])),1,3) in
('582','583','585','586','V56')
                    then Ch_prev5y_RD = 1;
          end;
          if length(strip(dia[i])) GE 4 then do;
                    if substr(upcase(strip(dia[i])),1,4) in
('N032','N033','N034','N035','N036','N037','N052','N053','N054','N055','N056','N057','N250','I120','I131','Z940','
Z992')
                              or substr(upcase(strip(dia[i])),1,4) in
('403A','403B','403X','588A','V42A','V45B')
                    then Ch_prev5y_RD = 1;
          end;
end;

/** Mild Liver Disease (Removed according to Ludvigson 2021:
'070C','070D','070E','070F','070G','070X','571','573D','573E','573W','573X','V42H','K700','K701','K702','K74',
'K709','K717','K713','K714','K715','K760','K762','K763','K764','K768','K769','Z944' ) **/
do i=1 to 31;
          if length(strip(dia[i])) GE 3 then do;
```

```
                    if substr(upcase(strip(dia[i])),1,3) in ('B15','B16','B17','B18','B19','K73')
                              or substr(upcase(strip(dia[i])),1,3) in ('070','573')
                    then Ch_prev5y_MILDLD = 1;
          end;
          if length(strip(dia[i])) GE 4 then do;
                    if substr(upcase(strip(dia[i])),1,4) in ('K703','K746','K754')
                              or substr(upcase(strip(dia[i])),1,4) in ('571C','571E','571F')
                    then Ch_prev5y_MILDLD = 1;
          end;
end;

/** Moderate or Severe Liver Disease (Removed according to Ludvigson 2021:
'572W','K704','K711','K721','K729','K765','K766','K767','I864') **/
do i=1 to 31;
          if length(strip(dia[i])) GE 3 then do;
                    if substr(upcase(strip(dia[i])),1,3) in ('R18')
                    then Ch_prev5y_MSLD = 1;
          end;
          if length(strip(dia[i])) GE 4 then do;
                    if substr(upcase(strip(dia[i])),1,4) in ('I850','I859','I982','I983')
                              or substr(upcase(strip(dia[i])),1,4) in
('456A','456B','456C','572C','572D','572E','789F')
                    then Ch_prev5y_MSLD = 1;
          end;
end;

/** Peptic Ulcer Disease (Removed according to Ludvigson 2021: - ) **/
do i=1 to 31;
          if length(strip(dia[i])) GE 3 then do;
                    if substr(upcase(strip(dia[i])),1,3) in ('K25','K26','K27','K28')
                              or substr(upcase(strip(dia[i])),1,3) in ('531','532','533','534')
                    then Ch_prev5y_PUD = 1;
          end;
end;

/** Cancer  (Removed according to Ludvigson 2021: - ) **/
do i=1 to 31;
          if length(strip(dia[i])) GE 3 then do;
                    if substr(upcase(strip(dia[i])),1,3) in
('C00','C01','C02','C03','C04','C05','C06','C07','C08','C09','C10','C11','C12','C13','C14','C15','C16','C17','C18','C19','
C20','C21',

          'C22','C23','C24','C25','C26','C30','C31','C32','C33','C34','C37','C38','C39','C40','C41','C43','C45','C
46','C47','C48','C49','C50','C51','C52','C53','C54','C55',

          'C56','C57','C58','C60','C61','C62','C63','C64','C65','C66','C67','C68','C69','C70','C71','C72','C73','C
74','C75','C76','C81','C82','C83','C84','C85','C88',
                                        'C90','C91','C92','C93','C94','C95','C96','C97')
                              or substr(upcase(strip(dia[i])),1,3) in
('140','141','142','143','144','145','146','147','148','149','150','151','152','153','154','155','156','157','158','159','1
60',

          '161','162','163','164','165','170','171','172','174','175','176','179','180','181','182','183','184','18
5','186','187','188','189','190','191','192','193','194',

          '195','200','201','202','203','204','205','206','207','208')
                    then Ch_prev5y_CANCER = 1;
```

```
		end;
		if length(strip(dia[i])) GE 4 then do;
			if substr(upcase(strip(dia[i])),1,4) in ('238G')
			then Ch_prev5y_CANCER = 1;
		end;
end;

/** Metastatic Carcinoma **/
do i=1 to 31;
		if length(strip(dia[i])) GE 3 then do;
			if substr(upcase(strip(dia[i])),1,3) in ('C77','C78','C79','C80')
				or substr(upcase(strip(dia[i])),1,3) in ('196','197','198')
			then Ch_prev5y_METS = 1;
		end;
		if length(strip(dia[i])) GE 4 then do;
			if substr(upcase(strip(dia[i])),1,4) in ('199A','199B')
			then Ch_prev5y_METS = 1;
		end;
end;

/**AIDS/HIV**/
do i=1 to 31;
		if length(strip(dia[i])) GE 3 then do;
			if substr(upcase(strip(dia[i])),1,3) in ('B20','B21','B22','B23','B24','R75')
			then Ch_prev5y_HIV = 1;
		end;
		if length(strip(dia[i])) GE 4 then do;
			if substr(upcase(strip(dia[i])),1,4) in ('079J','279K')
				or substr(upcase(strip(dia[i])),1,4) in
('F024','O987','Z114','Z219','Z711')
			then Ch_prev5y_HIV = 1;
		end;
end;

							end;
			end;
			when (%sysrc(_dsenom)) do; /* Ingen tr‰ff. Vill inte att det betyder error */
				 _error_=0;
			end;
				otherwise;
			end;
		end;
			if lopnr='' then
				lopnr = sparid;
			else
				klar = 1;
		end;
		output;
		sparid = lopnr;
		drop indatum hdia dia1-dia30 los klar i sparid;
run;

data d1; set data.store9819_comorb_Ch_prev5yr;
/* Remove allergy, adverse and late effects */
if length(strip(dx1)) GE 3 then do;
		if substr(upcase(strip(dx1)),1,3) eq 'T78' then delete;				* remove
allergy;
```

```
                    if substr(upcase(strip(dx1)),1,3) ge 'T80' then delete;                                    * remove
adverse and late effects;
                    if substr(upcase(strip(dx1)),1,3) ge 'Y40' and substr(upcase(strip(dx1)),1,3) le 'Y89' then delete;
end;

/* Recode sex */
if kon = 1 then sex = 0; *Male sex;
if kon = 2 then sex = 1; *Female sex;

/* Charlson index */
charlson_index = sum(of Ch_prev5y_MI--Ch_prev5y_DIAB_UC) * 1 + sum(of Ch_prev5y_DIAB_C--
Ch_prev5y_CANCER) * 2 + CH_prev5y_MSLD *
3 + Ch_prev5y_METS * 6 + Ch_prev5y_HIV * 6;

year = year(indate1);
admno = _n_;

ecodeflag1 = 0; ecodeflag2 = 0; ecodeflag3 = 0;
if ekod1_1 = '' AND (ekod2_1 NE '') then ecodeflag1 = 1;
if ekod1_1 = '' AND (ekod2_1 NE '' OR ekod3_1 NE '' OR ekod4_1 NE '' OR ekod5_1 NE '') then ecodeflag2 = 1;
if ekod1_1 = '' AND (ekod1_2 NE '' OR ekod1_3 NE '' OR ekod1_4 NE '' OR ekod1_5 NE '' OR ekod1_6 NE '' OR
                    ekod1_7 NE '' OR ekod1_8 NE '' OR ekod1_9 NE '' OR ekod1_10 NE '') then ecodeflag3 = 1;
ecodemiss = 0; if strip(ekod1_1) = '' then ecodemiss = 1;

DROP kon adm_no;
run;

proc freq data = d1; table ecodeflag1-ecodeflag3; title 'Pattern of missing in ecode variables'; run;
proc freq data = d1; table charlson_index; title 'Distribution Charlson index'; run;
proc freq data = d1; table ecodemiss; title 'Frequency e-code missing'; run;

/*******************************************************************************************
***
Program:        Matrix classification of injuries.
Purpose:        Classify injuries according to ICD-10 code with the CDC injury matrix and
                                        cause of injury according to CDC matrix.
Created:        2006-05 by S^ren Gustafsson
Modified:       2016-04-16 by Rolf Gedeborg
Notes:                          The code for classifying cause of injury is adapted from the SAS code on CDC
                                        homepage. In ver 2 cause of death data also adds to
classification if death is in
                                        hospital during injury admission (Ref. "Severity of Injury is
Underestimated in the Absence of
                                        Autopsy Verification" J Trauma. 2004;57:46ñ50). 2006-10-26
the code was revised
                                        so that for hospital deaths info is added from cause of death
data only when based
                                        on autopsy (clinical or forensic).
********************************************************************************************
***/

/* CHAPTER 1 - Check cause of injury codes. If causecode is missing for the first
department/hospital record of the admission, causecodes          from subsequent
department/hospital records are promoted */

data d2; set d1;
/* If first ekod is missing then promote from ekod5 to ekod1 consequtively */
```

```
                    if ekod4_1 = '' then ekod4_1 = ekod5_1;
                    if ekod3_1 = '' then ekod3_1 = ekod4_1;
                    if ekod2_1 = '' then ekod2_1 = ekod3_1;
                    if ekod1_1 = '' then ekod1_1 = ekod2_1;
/* If first ekod is still missing then promote from other records in patient registry belonging to the same injury
admission to
                    create one variable for the "primary" cause code */
causecode1 = ekod1_1;
                    if causecode1 = '' then causecode1 = ekod1_2;
                    if causecode1 = '' then causecode1 = ekod1_3;
                    if causecode1 = '' then causecode1 = ekod1_4;
                    if causecode1 = '' then causecode1 = ekod1_5;
                    if causecode1 = '' then causecode1 = ekod1_6;
                    if causecode1 = '' then causecode1 = ekod1_7;
                    if causecode1 = '' then causecode1 = ekod1_8;
                    if causecode1 = '' then causecode1 = ekod1_9;
                    if causecode1 = '' then causecode1 = ekod1_10;

allcausecode = CATX(' ', of ekod1_1-ekod1_10, of ekod2_1-ekod2_10, of ekod3_1-ekod3_10, of ekod4_1-
ekod4_10, of ekod5_1-ekod5_10);
run;

proc freq data = d2 order = freq; table causecode1 dbgrund1/ missing ; title 'Pattern of missing in ecode
variables'; run;

/* Check that the causecodes are accurate
data  d1a; set d1 (keep= causecode1 u_mortcaus);
array cause[2] $ causecode1 u_mortcaus;
do i=1 to 2;
                    if cause[i] NE '' AND substr(cause[i],1,1) IN('X' 'Y' 'V' 'W') then do;
                                        if substr(cause[i],2,1) NOT IN('0' '1' '2' '3' '4' '5' '6' '7' '8' '9') then
                                                            causecode=cause[i]; output;
                                        if substr(cause[i],3,1) NOT IN('0' '1' '2' '3' '4' '5' '6' '7' '8' '9') then
                                                            causecode=cause[i]; output;
                                        if substr(cause[i],4,1) NOT IN('0' '1' '2' '3' '4' '5' '6' '7' '8' '9') then
                                                            causecode=cause[i]; output;
                    end;
end;
run;

Summarize faulty codes and save to permanent dataset
proc freq data=d1a; tables causecode / out=trauma.freqcount_faultyEcode;
title 'Faulty causecodes in injury9804_noadveff'; run; */

/* !EVALUATION POINT: Note incorrect Ecodes from output */

/* CHAPTER 2 - Mend faulty Ecodes according to result above
------------------------------------------------------------------------------------------*/

/* !DATA ENTRY:  Enter faulty codes from result above and correct codes below

data d2; set d1;
array cause[2] $ causecode1 u_mortcaus;
do i=1 to 2;
if cause[i]='V03A' then cause[i]='V030';
if cause[i]='V03AA' then cause[i]='V0300';
if cause[i]='V03AB' then cause[i]='V0301';
```

```
if cause[i]='V11' then cause[i]='V119';
if cause[i]='V13' then cause[i]='V139';
if cause[i]='V18' then cause[i]='V189';
if cause[i]='V19' then cause[i]='V199';
if cause[i]='V22' then cause[i]='V229';
if cause[i]='V4' then cause[i]='V499';
if cause[i]='V43' then cause[i]='V439';
if cause[i]='V46' then cause[i]='V469';
if cause[i]='V47' then cause[i]='V479';
if cause[i]='V48' then cause[i]='V489';
if cause[i]='V49' then cause[i]='V499';
if cause[i]='V8' then cause[i]='V899';
if cause[i]='V80' then cause[i]='V809';
if cause[i]='W1' then cause[i]='';
if cause[i]='W11O' then cause[i]='W11';
if cause[i]='X50AA' then cause[i]='X50';
if cause[i]='X61**' then cause[i]='X61';
if cause[i]='X64BB' then cause[i]='X6411';
if cause[i]='X65A' then cause[i]='X650';
if cause[i]='XX649' then cause[i]='X649';
end;
drop i; run;
*/

/* CHAPTER 3 - Classify according to ICD-10 diag codes into injury matrix
------------------------------------------------------------------------------------------*/

/* Skapar informat som ˆvers‰tter ICD10 kod till cellreferens */
proc sql;
        create view work.vy_transl_4 as
                select icd10code as start,
                  dxcellref as label,
                        'dxcell'  as fmtname,
                        'I'       as type
                from eget.d_transl_4
        ;
quit;
proc format cntlin = work.vy_transl_4;
run;

/* Varje icd10 kod, 1 till 310 st, ˆvers‰tts till en cellreferens     */
/* Varje cellreferens leder till att variabeln dxcell med samma nr   */
/* som cellreferensen s‰tts till sant(1). Maxv‰rdet som ‰r ok ‰r 152 */
/* Hˆgre v‰rde pÂ cellreferens leder till utskrift i loggen          */
/* Anv‰nder array fˆr dxcellref fˆr enklare kontroll/felsˆkning      */
data d3; set d2;/*
        causecode2-causecode10 death cdeath c4);*/
*       length text $100;

*       array dxcellref (310) dxcellref1-dxcellref310;
*       array dx (310) dx1-dx310;
*       array dxcellref_d (50) dxcellref_d1-dxcellref_d50;
*       array dx_d (50) kap19 morsak1-morsak48 ulorsak;
*       array dxcell (152) dxcell1-dxcell152;
        /* Nollst‰ll alla dxcell infˆr varje loop */
*       do i = 1 to 152;
*               dxcell(i) = 0;
```

```
*                     end;

/* Identify hospital admission and classify from discharge codes
if dx1 NE '' then
                      do i = 1 to 310;
                                            dxcellref(i) = input(substr(dx(i),1,4),?? dxcell.);
                                            if dxcellref(i) ne . then do;
                                                                  if dxcellref(i) > 152 then do;
                                                                                        text = 'Cellref='||put(dxcellref(i),7.)||' i obs
'||put(_N_,7.);
                                                                                        put text;
                                                                  end;
                                                                  else
                                                                                        dxcell(dxcellref(i)) = 1;
                                            end;
                      end;
otherwise classify from cause of death diagnoses, use cause of death data if hosp mort AND
                      autopsy
if adm_status1 = '4' OR (ABS(dodsdatn-max(of outdate1-outdate10)) LE 1 AND dbgrund1 NE '')
                      then
                      do j = 1 to 50;
                                            dxcellref_d(j) = input(substr(dx_d(j),1,4),?? dxcell.);
                                            if dxcellref_d(j) ne . then do;
                                                                  if dxcellref_d(j) > 152 then do;
                                                                                        text = 'Cellref='||put(dxcellref_d(j),7.)||' i obs
'||put(_N_,7.);
                                                                                        put text;
                                                                  end;
                                                                  else
                                                                                        dxcell(dxcellref_d(j)) = 1;
                                            end;
                      end;
*/
/* CHAPTER 4 - Classify cause of injury based on CDC matrix
                      ----------------------------------------------------------------------------------*/
format c4 1.;

if causecode1 NE '' then do;                      * classify from discharge codes;
                      if length(causecode1) GE 3 then death = substr(strip(causecode1),1,3); else death = '';
                      if length(causecode1) GE 4 then cdeath = substr(strip(causecode1),1,4); else cdeath = '';
                      format c4 1.;
                      if length(causecode1) GE 4 then c4 = input(substr(strip(causecode1),4,1),1.); else c4 = '';
                      end;
else do;                                                                                                    * classify from
cause of death codes;
                      if length(strip(ulorsak)) GE 3 then do;
                                            death = substr(strip(ulorsak),1,3);
                                            end;
                      if length(strip(ulorsak)) GE 4 then do;
                                            cdeath = substr(strip(ulorsak),1,4);
                                            c4 = input(substr(strip(ulorsak),4,1),1.);
                                            end;
                      end;

                      IF ('W25'<=DEATH<='W29' OR DEATH='W45') OR
                      (DEATH='X78') OR
                      (DEATH='X99') OR
```

```
(CDEATH='Y354') OR
(DEATH='Y28') THEN CAUSE=00;

IF ('W65'<=DEATH<='W74') OR
(DEATH='X71') OR
(DEATH='X92') OR
(DEATH='Y21') THEN CAUSE=01;

IF ('W00'<=DEATH<='W19') OR
(DEATH='X80') OR
(DEATH='Y01') OR
(DEATH='Y30') THEN CAUSE=02;

IF ('X00'<=DEATH<='X09') OR
(DEATH='X76') OR
(DEATH='X97') OR
(CDEATH='Y363') OR
(DEATH='Y26') OR (CDEATH='U013') THEN CAUSE=03;

IF ('X10'<=DEATH<='X19') OR
(DEATH='X77') OR
(DEATH='X98' ) OR
(DEATH='Y27') THEN CAUSE=04;

IF ('W32'<=DEATH<='W34') OR
('X72'<=DEATH<='X74') OR
('X93'<=DEATH<='X95') OR
(CDEATH='Y350') OR
('Y22'<=DEATH<='Y24') OR (CDEATH='U014') THEN CAUSE=05;

IF (DEATH='W24' OR 'W30'<=DEATH<='W31') THEN CAUSE=06;

IF (('V30'<=DEATH<='V79' AND 4<=C4<=9) OR
('V81'<=DEATH<='V82' AND C4=1) OR ('V83'<=DEATH<='V86'
AND 0<=C4<=3)) OR
(('V20'<=DEATH<='V28' AND 3<=C4<=9) OR
(DEATH='V29' AND (4<=C4<=9))) OR
(('V12'<=DEATH<='V14' AND 3<=C4<=9) OR
(DEATH='V19' AND (4<=C4<=6 ))) OR
(('V02'<=DEATH<='V04' AND C4 IN (1,9)) OR
CDEATH='V092') OR
(DEATH='V80' AND 3<=C4<=5) OR
((DEATH='V87' AND 0<=C4<=8) OR CDEATH='V892') THEN CAUSE=07;

IF (DEATH IN ('V10','V11','V15','V16','V17','V18') OR
('V12'<=DEATH<='V14' AND 0<=C4<=2) OR
(DEATH='V19' AND 0<=C4<=3) OR CDEATH='V198'
OR CDEATH='V199' ) THEN CAUSE=08;

IF ((DEATH IN ('V01','V05','V06')) OR
('V02'<=DEATH<='V04' AND C4=0) OR (DEATH='V09'
AND C4 IN (0,1,3,9)) ) THEN CAUSE=09;

IF ('V20'<=DEATH<='V28' AND 0<=C4<=2) OR
(DEATH='V29' AND 0<=C4<=3) OR
('V30'<=DEATH<='V79' AND 0<=C4<=3) OR
(DEATH IN ('V80') AND C4 IN (0,1,2,6,7,8,9)) OR
```

```
(DEATH IN ('V81','V82') AND C4 IN (0,2,3,4,5,6,7,8,9)) OR
('V83'<=DEATH<='V86' AND 4<=C4<=9) OR
(CDEATH IN ('V879')) OR
(DEATH IN ('V88') AND 0<=C4<=9) OR
(DEATH IN ('V89') AND C4 IN (0,1,3,9)) OR
(DEATH='X82' OR DEATH='Y03' OR DEATH='Y32') THEN CAUSE=10;

IF ('V90' <=DEATH<='V99') OR
(CDEATH='Y361') OR (CDEATH='U011') THEN CAUSE=11;

IF ('W92'<=DEATH<='W99' OR
'X20'<=DEATH<='X39' OR
'X51'<=DEATH<='X57' OR
'W42'<=DEATH<='W43' OR
'W53'<=DEATH<='W64') THEN CAUSE=12;

IF (DEATH='X50') THEN CAUSE=13;

IF ('X40'<=DEATH<='X49') OR
('X60'<=DEATH<='X69') OR
('X85'<=DEATH<='X90') OR
('Y10'<=DEATH<='Y19') OR
(CDEATH='Y352') OR ('U016'<=CDEATH<='U017') THEN CAUSE=14;

IF ('W20'<=DEATH<='W22' OR
'W50'<=DEATH<='W52') OR
(DEATH='X79') OR
(DEATH='Y00' OR DEATH='Y04') OR
(CDEATH='Y353') OR
(DEATH='Y29') THEN CAUSE=15;

IF ('W75'<=DEATH<='W84') OR
(DEATH='X91') OR
(DEATH='X70') OR
(DEATH='Y20') THEN CAUSE=16;

IF (DEATH='W23' OR
'W35'<=DEATH<='W41' OR
DEATH IN ('W44','W49','Y85') OR
'W85'<=DEATH<='W91') OR
(DEATH IN ('X75','X81')) OR
(DEATH IN ('X96','Y02') OR
'Y05'<=DEATH<='Y07') OR
(CDEATH IN ('Y351','Y355', 'Y360',
'Y362') OR 'Y364'<=CDEATH<='Y368') OR
(DEATH IN ('Y25','Y31')) OR (CDEATH IN('U010', 'U012', 'U015', 'U030')) THEN CAUSE=17;

IF (DEATH='X58' OR DEATH='Y86') OR
(DEATH='X83' OR CDEATH='Y870') OR
(DEATH='Y08' OR CDEATH='Y871') OR
(CDEATH IN ('Y356','Y890','Y891')) OR
(DEATH='Y33' OR CDEATH='Y872') OR CDEATH='U018' OR DEATH='U02' THEN CAUSE=18;

IF (DEATH='X59') OR
(DEATH='X84') OR
(DEATH='Y09') OR
(CDEATH IN ('Y357','Y369')) OR
```

```
                    (DEATH='Y34' OR CDEATH='Y899') OR CDEATH='U019' OR CDEATH='U039' THEN CAUSE=19;

                    IF (('V30'<=DEATH<='V79' AND 4<=C4<=9) OR
                    ('V83'<=DEATH<='V86'
                    AND 0<=C4<=3)) THEN MVTPER=1;

                    IF (('V20'<=DEATH<='V28' AND 3<=C4<=9) OR
                    (DEATH='V29' AND (4<=C4<=9))) THEN MVTPER=2;

                    IF (('V12'<=DEATH<='V14' AND 3<=C4<=9) OR
                    (DEATH='V19' AND (4<=C4<=6 ))) THEN MVTPER=3;

                    IF (('V02'<=DEATH<='V04' AND C4 IN (1,9)) OR
                    CDEATH='V092' ) THEN MVTPER=4;

                    IF (DEATH='V80' AND 3<=C4<=5) OR
                    ('V81'<=DEATH<='V82' AND C4=1) THEN MVTPER=5;

                    IF ((DEATH='V87' AND 0<=C4<=8) OR CDEATH='V892') THEN MVTPER=6;


                    IF ('V01'<=DEATH<='X59') OR DEATH='Y85' OR DEATH='Y86' THEN INJS=1;
                    IF ('X60'<=DEATH<='X84') OR CDEATH='Y870' OR DEATH='U03' THEN INJS=2;
                    IF ('X85'<=DEATH<='Y09') OR CDEATH='Y871' OR 'U01'<=DEATH<='U02' THEN INJS=3;
                    IF ('Y10'<=DEATH<='Y34') OR CDEATH='Y872' OR CDEATH='Y899' THEN INJS=4;

                    IF DEATH='Y35' OR DEATH='Y36' OR (DEATH='Y89' AND 0<=C4<=1) THEN INJS=5;

                    IF ('Y40'<=DEATH<='Y84') OR DEATH='Y88' THEN ADV=1;
drop hdiax death cdeath c4;
run;

proc freq data = d3; table cause injs mvtper adv; title 'Check cause of injury categories'; run;

/* Check injury diagnoses and calculate ICISS based on published DSPs (Gedeborg et al 2014). Mortality
diagnoses are used when death
was in hospital and autopsy performed */
data d4; set d3 (keep = lopnr admno adm_status1 dx1-dx310 deathdate ulorsak morsak1-morsak48 kap19
dbgrund1 outdate1-outdate10);
format mdx1-mdx50 $8. icd9dx icd10dx 1.; icd9dx = 0; icd10dx = 10;
array a_mortdxall(*) $ ulorsak morsak1-morsak48 kap19;
array a_mortdxuse(*) $ dx311-dx360;

do i=1 to 50;
                    if abs(deathdate-max(of outdate1-outdate10)) LE 1 AND strip(dbgrund1) in('1','2','3','4') OR
                                        adm_status1 = '4' then a_mortdxuse[i] = a_mortdxall[i];
end;

array a_dx(*) $ dx1-dx310 mdx1-mdx50;

do i=1 to 360;
if strip(upcase(a_dx[i])) = 'S00' then a_dx[i] = 'S009';
                                                                      *modify 3-position codes;
if strip(upcase(a_dx[i])) = 'S01' then a_dx[i] = 'S019';
if strip(upcase(a_dx[i])) = 'S02' then a_dx[i] = 'S029';
if strip(upcase(a_dx[i])) = 'S05' then a_dx[i] = 'S059';
if strip(upcase(a_dx[i])) = 'S06' then a_dx[i] = 'S069';
```

```
if strip(upcase(a_dx[i])) = 'S09' then a_dx[i] = 'S099';
if strip(upcase(a_dx[i])) = 'S12' then a_dx[i] = 'S129';
if strip(upcase(a_dx[i])) = 'S16' then a_dx[i] = 'S169';

if strip(upcase(a_dx[i])) = 'S16X' then a_dx[i] = 'S169';
if strip(upcase(a_dx[i])) = 'S18' then a_dx[i] = 'S189';
if strip(upcase(a_dx[i])) = 'S18X' then a_dx[i] = 'S189';
if strip(upcase(a_dx[i])) = 'S30' then a_dx[i] = 'S309';
if strip(upcase(a_dx[i])) = 'S31' then a_dx[i] = 'S319';
if strip(upcase(a_dx[i])) = 'S32' then a_dx[i] = 'S329';
if strip(upcase(a_dx[i])) = 'S35' then a_dx[i] = 'S359';
if strip(upcase(a_dx[i])) = 'S37' then a_dx[i] = 'S379';
if strip(upcase(a_dx[i])) = 'S41' then a_dx[i] = 'S419';
if strip(upcase(a_dx[i])) = 'S42' then a_dx[i] = 'S429';
if strip(upcase(a_dx[i])) = 'S43' then a_dx[i] = 'S439';
if strip(upcase(a_dx[i])) = 'S47' then a_dx[i] = 'S479';
if strip(upcase(a_dx[i])) = 'S47X' then a_dx[i] = 'S479';
if strip(upcase(a_dx[i])) = 'S50' then a_dx[i] = 'S509';
if strip(upcase(a_dx[i])) = 'S51' then a_dx[i] = 'S519';
if strip(upcase(a_dx[i])) = 'S52' then a_dx[i] = 'S529';
if strip(upcase(a_dx[i])) = 'S53' then a_dx[i] = 'S539';
if strip(upcase(a_dx[i])) = 'S54' then a_dx[i] = 'S549';
if strip(upcase(a_dx[i])) = 'S56' then a_dx[i] = 'S569';
if strip(upcase(a_dx[i])) = 'S72' then a_dx[i] = 'S729';
if strip(upcase(a_dx[i])) = 'S73' then a_dx[i] = 'S739';
if strip(upcase(a_dx[i])) = 'S80' then a_dx[i] = 'S809';
if strip(upcase(a_dx[i])) = 'S81' then a_dx[i] = 'S819';
if strip(upcase(a_dx[i])) = 'S82' then a_dx[i] = 'S829';
if strip(upcase(a_dx[i])) = 'S96' then a_dx[i] = 'S969';
if strip(upcase(a_dx[i])) = 'S97' then a_dx[i] = 'S979';
if strip(upcase(a_dx[i])) = 'T00' then a_dx[i] = 'T009';
if strip(upcase(a_dx[i])) = 'T18' then a_dx[i] = 'T189';
if strip(upcase(a_dx[i])) = 'T20' then a_dx[i] = 'T209';
if strip(upcase(a_dx[i])) = 'T21' then a_dx[i] = 'T219';
if strip(upcase(a_dx[i])) = 'T22' then a_dx[i] = 'T229';
if strip(upcase(a_dx[i])) = 'T23' then a_dx[i] = 'T239';
if strip(upcase(a_dx[i])) = 'T24' then a_dx[i] = 'T249';
if strip(upcase(a_dx[i])) = 'T27' then a_dx[i] = 'T279';
if strip(upcase(a_dx[i])) = 'T38' then a_dx[i] = 'T389';
if strip(upcase(a_dx[i])) = 'T39' then a_dx[i] = 'T399';
if strip(upcase(a_dx[i])) = 'T40' then a_dx[i] = 'T409';
if strip(upcase(a_dx[i])) = 'T42' then a_dx[i] = 'T429';
if strip(upcase(a_dx[i])) = 'T43' then a_dx[i] = 'T439';
if strip(upcase(a_dx[i])) = 'T50' then a_dx[i] = 'T509';
if strip(upcase(a_dx[i])) = 'T51' then a_dx[i] = 'T519';
if strip(upcase(a_dx[i])) = 'T54' then a_dx[i] = 'T549';
if strip(upcase(a_dx[i])) = 'T01' then a_dx[i] = 'T019';
if strip(upcase(a_dx[i])) = 'T07' then a_dx[i] = 'T079';
if strip(upcase(a_dx[i])) = 'T08' then a_dx[i] = 'T089';
if strip(upcase(a_dx[i])) = 'T10' then a_dx[i] = 'T109';
if strip(upcase(a_dx[i])) = 'T07X' then a_dx[i] = 'T079';
if strip(upcase(a_dx[i])) = 'T08' then a_dx[i] = 'T089';
if strip(upcase(a_dx[i])) = 'T08X' then a_dx[i] = 'T089';
if strip(upcase(a_dx[i])) = 'T08x' then a_dx[i] = 'T089';
if strip(upcase(a_dx[i])) = 'T10' then a_dx[i] = 'T109';
if strip(upcase(a_dx[i])) = 'T10X' then a_dx[i] = 'T109';
if strip(upcase(a_dx[i])) = 'T10x' then a_dx[i] = 'T109';
```

```
if strip(upcase(a_dx[i])) = 'T12' then a_dx[i] = 'T129';
if strip(upcase(a_dx[i])) = 'T12X' then a_dx[i] = 'T129';
if strip(upcase(a_dx[i])) = 'T12x' then a_dx[i] = 'T129';
if strip(upcase(a_dx[i])) = 'T16' then a_dx[i] = 'T169';
if strip(upcase(a_dx[i])) = 'T16X' then a_dx[i] = 'T169';
if strip(upcase(a_dx[i])) = 'T30' then a_dx[i] = 'T300';
if strip(upcase(a_dx[i])) = 'T46' then a_dx[i] = 'T469';
if strip(upcase(a_dx[i])) = 'T55' then a_dx[i] = 'T559';
if strip(upcase(a_dx[i])) = 'T55X' then a_dx[i] = 'T559';
if strip(upcase(a_dx[i])) = 'T58' then a_dx[i] = 'T589';
if strip(upcase(a_dx[i])) = 'T58X' then a_dx[i] = 'T589';
if strip(upcase(a_dx[i])) = 'T59' then a_dx[i] = 'T599';
if strip(upcase(a_dx[i])) = 'T64' then a_dx[i] = 'T649';
if strip(upcase(a_dx[i])) = 'T64X' then a_dx[i] = 'T649';
if strip(upcase(a_dx[i])) = 'T66' then a_dx[i] = 'T669';
if strip(upcase(a_dx[i])) = 'T68' then a_dx[i] = 'T689';
if strip(upcase(a_dx[i])) = 'T68X' then a_dx[i] = 'T689';
if strip(upcase(a_dx[i])) = 'T71' then a_dx[i] = 'T719';
if strip(upcase(a_dx[i])) = 'T71X' then a_dx[i] = 'T719';
if strip(upcase(a_dx[i])) = 'T84' then a_dx[i] = 'T849';
if strip(upcase(a_dx[i])) = 'T96' then a_dx[i] = 'T969';
if strip(upcase(a_dx[i])) = 'T97' then a_dx[i] = 'T979';
if strip(upcase(a_dx[i])) = 'T74' then a_dx[i] = 'T749';
if strip(upcase(a_dx[i])) = 'T75' then a_dx[i] = 'T759';

if a_dx[i] NE '' then do;
            if substr(strip(a_dx[i]),1,1) in ('8','9') then icd9dx = 1;
            if substr(strip(upcase(a_dx[i])),1,1) in ('S','T') then icd10dx = 1;
            end;
end;

drop i;
run;

proc freq data = d4; table icd9dx icd10dx; title 'Occurence of ICD9 injury codes'; run;

/* Transpose to long format */
proc transpose data = d4
   out = dx_for_iciss_long prefix = dx;
   by admno;
            var dx1-dx360;
run;

proc datasets lib = work nolist;
            delete d1 d2 d4; run; quit;

/* Check ICD-10 codes are valid. If not add to step above. */
data d5; set dx_for_iciss_long;
if dx1 NE '' then do;
            if substr(strip(upcase(dx1)),1,1) in('S','T') AND length(dx1) = 3;
            end;
run;

proc freq data = d5; table dx1; run;

/* Remove lines with diagnosis missing */
data dx_for_iciss_long; set dx_for_iciss_long;
```

```
if length(strip(dx1)) GE 1 then do;
			if substr(strip(upcase(dx1)),1,1) in('S','T') then icd10code = substr(strip(upcase(dx1)),1,4);
			end;
if icd10code = '' then delete;
if icd10code > 'T799' then delete;
									*delete excluded codes;
if icd10code NE '' AND length(icd10code) GE 3 then do;
			if substr(strip(icd10code),1,3) = 'T78' then delete;
			end;
drop dx1; run;

proc sort data = dx_for_iciss_long nodupkey; by admno icd10code; run;		*remove duplicate diagnosis
codes;
proc sort data = dx_for_iciss_long; by icd10code; run;

/* Generate dataset with log(DSP) */
data work.logdsp_ice_allage; set data.dsp_ice_allage;
if dsp_ice_sum_allage = 0 then dsp_ice_sum_allage = 0.0000001;
logdsp_ice_sum_allage = log(dsp_ice_sum_allage);
keep icd10code logdsp_ice_sum_allage; run;

proc sort data = work.logdsp_ice_allage; by icd10code; run;

data dx_for_iciss_long; merge dx_for_iciss_long(IN=a) work.logdsp_ice_allage(IN=b); by icd10code; if a;
if substr(strip(icd10code),1,1) NOT IN('S', 'T') then delete; run;

proc sort data = dx_for_iciss_long; by admno _name_; run;

/* Calculate the score */
proc means data = dx_for_iciss_long sum noprint; by admno; var logdsp_ice_sum_allage;
			output out = d_out(drop = _type_ _freq_)
			sum(logdsp_ice_sum_allage) = logiciss_sum; run;

data d_out2; set d_out;
iciss = exp(logiciss_sum);
drop logiciss_sum;
run;

/* CHECKPOINT: Merge to an analysis dataset. VERIFY CORRECT ANALYSIS DATASET NAME */
proc sort data = d3; by admno; run;
proc sort data = d_out2; by admno; run;
data data.injury9819_2_samedate; merge d3(IN=a) d_out2(IN=b); by admno; if a OR b;
format sex sexf. cause causef. mvtper mvtperf. adm_status1-adm_status10 $admstf. disch_status1-
disch_status10 $disstf.
lt_klin1-lt_klin10 mvof. region $lanf.;

if geo = '' AND lk NE '' then region = substr(lk,1,2);
if lk = '' AND geo NE '' then region = substr(geo,1,2);
drop pvard;
run;

/* Describe variables */
proc freq data = data.injury9819_2; table region; title 'Distribution region variable'; run;
proc freq data = data.injury9819_2; table lt_klin1; title 'Distribution type dept'; run;

data d_temp; set data.injury9819_2 (keep = causecode1);
causecheck = 0; if causecode1 = '' then causecheck = 1; run;
```

```
proc freq data = d_temp; table causecheck; title 'Missing cause of injury'; run;



/* Describe and verify ICISS score calculation */
proc means data = data.injury9819_2 n nmiss mean min P10 P25 median P75 P90 max; var iciss age; title
'Check ICISS and age variable'; run;
proc freq data = data.injury9819_2; table age; run;

ODS GRAPHICS / MAXOBS = 2397556;
proc sgplot data = data.injury9819_2; histogram age; density age / type = kernel; run;
proc sgplot data = data.injury9819_2; histogram iciss; density iciss / type = kernel; run;

data d_temp; set data.injury9819_2;
if iciss = .; run;
proc freq data = d_temp; table adm_status1; run;

proc means data = data.injury9819_2; var age; title 'Verify data'; run;
proc freq data = d3; table year sex charlson_index / missing; title 'Verify data'; run;

proc sort data = data.injury9819_2; by sex; run;
proc sgplot data = data.injury9819_2; by sex; histogram age; density age / type = kernel; run;

/* Check if main dx is the same for first and second department */
data d_temp; set data.injury9819_2 (keep = dx1 dx11);
if strip(dx11) NE '' then do;
                dx_eq = 0;
                if strip(dx1) = strip(dx11) then dx_eq = 1;
                end;
run;

proc freq data = d_temp; table dx_eq / missing; run;
proc freq data = data.injury9819_2; table dep_change / missing; run;

/* Check individual records against original data */
proc surveyselect data = data.injury9819_2
method = srs
n = 1
out = check_final; run;

data check_par; set datasos.t_t_t_r_par_sv_7178_2020;
if lopnr = 5132304; run;

/* Fix region */
data data.region; set datasos.t_t_t_r_par_sv_7178_2020 (keep = lopnr indatuma lk lkf);
indate1 = input(indatuma, YYMMDD10.);
if strip(lkf) NE '' then region = substr(strip(lkf), 1, 2);
if region = '' and strip(lk) NE '' then region = substr(strip(lk), 1, 2);
if region in ('40', '88', '99') then region = '';
run;

proc freq data = data.region; table region / missing; title 'Frequency region'; run;

data data.injury9819_3; set data.injury9819_2 (drop = region); run;
proc sort data = data.injury9819_3; by lopnr indate1; run;

proc sort data = data.region; by lopnr indate1; run;
```

```
data data.injury9819_3; merge data.injury9819_3 (IN = a) data.region (IN = b); by lopnr indate1; if a; run;

proc freq data = data.injury9819_3; table region / missing; title 'Frequency region'; run;



/************************************************************************************
Program:        Count hip fx
Purpose:        For Karl Michaelsson and UmeÂ group
Created:        2016-12-01
Revised:        2023-01-21
By:                                     Rolf Gedeborg
Notes:                          2019-01-24 added analysis of mean age
                                        2019-01-27 added sensitivity analysis to look in secondary dx
                                        2022-02-19: Changed to same date to define transfer between
departments
                                        2022-04-30: Fix bug that failed to implement prediction model
for incident injuries
                                        2023-01-21: Added deathdate to dataset with naive selection
hip fx
*************************************************************************************/

/* Set libraries */
libname data  "\\micro.intra\projekt\P0660$\P0660_Gem"; run;
libname lin  "\\micro.intra\projekt\P0660$\P0660_Gem\Linkoping"; run;
libname formats  "\\micro.intra\projekt\P0660$\P0660_Gem\Formats"; run;
libname datasos  "\\micro.intra\projekt\P0660$\P0660_Data\Leverans_20210831"; run;
libname datadors  "\\micro.intra\projekt\P0660$\P0660_Data\Leverans_20210322"; run;

options fmtsearch = (formats);

proc contents data = data.injury9819_3; title 'Dataset content: Trauma analysis dataset'; run;


data data.hipfxincid; set data.injury9819_3;

if adm_status1 = '4' then delete;

array dx_a(31) $ dx1 dx11 dx21 dx31 dx41 dx51 dx61 dx71 dx81 dx91 dx101 dx111 dx121 dx131 dx141 dx151
dx161 dx171 dx181 dx191 dx201
                                                dx211 dx221 dx231 dx241 dx251 dx261 dx271
dx281 dx291 dx301;
array dx_a2(330) $ dx1 - dx330;

hipfxall = 0; hipfxall2 = 0; hipfxmain = 0; hipfx_otherinj = 0;

do i = 1 to 31;
        if length(compress(dx_a(i), ' *-+.')) GE 3 then do;
        if substr(compress(dx_a(i), ' *-+.'),1,3) in('820') then hipfxall = 1;
        end;
        if length(compress(dx_a(i), ' *-+.')) GE 4 then do;
        if substr(compress(dx_a(i), ' *-+.'),1,4) in('S720','S721','S722') then hipfxall = 1;
        end;
end;

do i = 1 to 330;
```

```
			if length(compress(dx_a2(i), ' *-+.')) GE 3 then do;
			if substr(compress(dx_a2(i), ' *-+.'),1,3) in('820') then hipfxall2 = 1;
			if substr(compress(dx_a2(i), ' *-+.'),1,3) NOT in('820') AND
					substr(compress(dx_a2(i), ' *-+.'),1,1) in('8') then hipfx_otherinj = 1;
			end;
			if length(compress(dx_a2(i), ' *-+.')) GE 4 then do;
			if substr(compress(dx_a2(i), ' *-+.'),1,4) in('S720','S721','S722') then hipfxall2 = 1;
			if substr(compress(dx_a2(i), ' *-+.'),1,4) NOT in('S720','S721','S722') AND
					substr(compress(dx_a2(i), ' *-+.'),1,1) in('S', 'T') then hipfx_otherinj = 1;
			end;
end;

if hipfxall2 = 1;

if substr(compress(dx1, ' *-+.'),1,4) in('S720','S721','S722','820') then hipfxmain = 1;

if length(compress(dx1, ' *-+.')) GE 3 then do;
			if substr(compress(dx1, ' *-+.'),1,3) in('820') then hipfxall2 = 1;
			end;
if length(compress(dx1, ' *-+.')) GE 4 then do;
			if substr(compress(dx1, ' *-+.'),1,4) in('S720','S721','S722') then hipfxall2 = 1;
			end;

disch_status = disch_status10;
if disch_status = '' then disch_status = disch_status9;
if disch_status = '' then disch_status = disch_status8;
if disch_status = '' then disch_status = disch_status7;
if disch_status = '' then disch_status = disch_status6;
if disch_status = '' then disch_status = disch_status5;
if disch_status = '' then disch_status = disch_status4;
if disch_status = '' then disch_status = disch_status3;
if disch_status = '' then disch_status = disch_status2;
if disch_status = '' then disch_status = disch_status1;

format los 5.; label los = 'Length of stay (days)';
los = intck('day', indate1, max(of outdate1-outdate10)); if los = 0 then los = 0.5;

if age GE 50;
keep lopnr indate1 year age sex charlson_index region iciss hipfxall hipfxall2 hipfxmain hipfx_otherinj
deathdate los disch_status susp_readm;
run;

data d1a; set data.hipfxincid; if hipfxmain = 1; run;
proc freq data = d1a; table year * age * sex; format age age5y65f.; title 'Count incident hip fracture'; run;
proc freq data = d1a; table year * sex; title 'Count incident hip fracture'; run;
proc freq data = d1a; table hipfx_otherinj; title 'Count other injuries'; run;
proc freq data = d1a; table susp_readm; title 'Count suspected readmissions'; run;




proc means data = d1a maxdec = 2; class year sex; var age; run;

data d1b; set d1; if hipfxall = 1; run;
proc freq data = d1b; table hipfxmain; title 'Hipfx any position first department'; run;

data d1c; set d1; if hipfxall2 = 1; run;
```

```
proc freq data = d1c; table hipfxmain; title 'Hipfx any position'; run;
proc freq data = d1c; table year * age * sex; format age age5y65f.; title 'Count hip fracture, incident cases, any
position'; run;



/* Naive analysis */
proc contents data = datasos.t_t_t_r_par_sv_7178_2020; title 'Dataset content: PAR (hosp discharge registry)';
run;

data data.hipfxall9819; set datasos.t_t_t_r_par_sv_7178_2020 (keep = lopnr indatuma utdatuma kon hdia dia1
- dia30 foddat);
array dx(31) $ hdia dia1 - dia30;

hipfxdx = 0; hipfxmaindx = 0;

if length(compress(hdia, ' *-+.')) GE 3 then do;
                        if substr(compress(hdia, ' *-+.'),1,3) in('820') then hipfxmaindx = 1;
                        end;
            if length(compress(hdia, ' *-+.')) GE 4 then do;
                        if substr(compress(hdia, ' *-+.'),1,4) in('S720','S721','S722') then hipfxmaindx =
1;
            end;

do i = 1 to 31;
            if length(compress(dx(i), ' *-+.')) GE 3 then do;
                        if substr(compress(dx(i), ' *-+.'),1,3) in('820') then hipfxdx = 1;
                        end;
            if length(compress(dx(i), ' *-+.')) GE 4 then do;
                        if substr(compress(dx(i), ' *-+.'),1,4) in('S720','S721','S722') then hipfxdx = 1;
                        end;
            end;

if hipfxdx = 1;

format indate outdate bdate YYMMDD10. age 3.1;
indate = input(indatuma, YYMMDD10.);
outdate = input(utdatuma, YYMMDD10.);
bdate = mdy(substr(strip(foddat),5,2), '15', substr(strip(foddat),1,4));
age = intck('DAY', bdate, indate) / 365.25;
year = year(indate);

if year > 1997;

/* Recode sex */
if kon = 1 then sex = 0; *Male sex;
if kon = 2 then sex = 1; *Female sex;

drop indatuma utdatuma kon hdia dia1 - dia30 foddat;
run;

proc sort data = data.hipfxall9819 out = data.hipfxall9819first nodupkey; by lopnr; run;

data data.hipfxall9819b; set data.hipfxall9819;
if hipfxmaindx = 1; run;
proc sort data = data.hipfxall9819b out = data.hipfxall9819firstmain nodupkey; by lopnr; run;
```

```
proc freq data = data.hipfxall9819; table year * sex; title 'Count naive incident hip fracture'; run;

data d3; set datasos.par_sv_8714_ny (keep = ar ekod1-ekod5);
cause_miss = 0;
if ekod1 = '' AND if ekod2 = '' AND if ekod3 = '' AND if ekod4 = '' AND if ekod5 = '' then cause_miss = 1;
run;

Proc freq data = d3; table cause_miss * ar; run;

/* Dataset for competing risk analysis naive hip dx and risk for second fracture. Keep all records with main dx
hip fx and add deathdate */


proc sort data = data.hipfxall9819 out = data.hipfxall9819first nodupkey; by lopnr; run;

data data.hipfxall9819b; set data.hipfxall9819;
if hipfxmaindx = 1; run;
proc sort data = data.hipfxall9819b out = data.hipfxall9819firstmain nodupkey; by lopnr; run;


proc freq data = data.hipfxall9819; table year * sex; title 'Count naive incident hip fracture'; run;


/* Create dataset with death dates */
data data.deaths; set datadors.t_r_dors__7178_2020 (keep = lopnr DODSDAT);
format deathdate YYMMDD10.;
if substr(DODSDAT,5,2) = '00' then substr(DODSDAT,5,2) = '06';
if substr(DODSDAT,7,2) = '00' then substr(DODSDAT,7,2) = '15';
deathdate = input(DODSDAT, YYMMDD10.);
if deathdate NE .;
drop DODSDAT;
run;
```

Supplementary File 2. The SAS code used by the Swedish National Board of Health and Welfare (https://www.socialstyrelsen.se/en) to calculate the official annual number of hip fractures in Sweden. The SAS code also displays the authority's approach when calculating all annual hip fracture events (the naïve approach) and the yearly number of first hip fractures.

```
data egtask.svov(keep=ar lt sjukhus reg pseudo pnrq indatum utdatum pnrq hdia diagnos kon alder_s
hfrakt) ;
set
a_par.a_par_sv_1: (where=(1964<=ar<=2010))
a_par.a_par_sv_2: (where=(2011<=ar<=2014))
a_par.a_par_sv_2015_ver5
a_par.a_par_sv_2016_ver3(rename=(lk=lkf))
a_par.a_par_sv_2017_ver3(rename=(lk=lkf));
where pnrq in('0','4') and kon in ('1','2') and put(lkf,$2.) between '01' and '25' and
    alder_s>=0 ;
            Do i =1 to 30;
            sc=scan (diagnos,i,'');
            if sc >' ' and sc in :('820','S720','S721','S722')then do;
            hfrakt='Höftfraktur';
output;
end;
end;
run;

proc format;
value age
0-49='< 50'
50-high=[ald20x.];
run;
```

```
/*Incidenta fall;*/

proc sort data=egtask.svov;
by pseudo indatum;
run;

data nyafall;
set egtask.svov;
by pseudo indatum;
if pseudo >' ' and first.pseudo and ar => 1964 then inc=1; run;

/* Antal per år */
proc sort data=egtask.svov nodupkey out=egtask.svov_p;
by ar pseudo;
run;

ods excel file="H:\data\twans\data\Karl Michaëlsson dnr 33936_2018x.xlsx"
options(sheet_name='Alla HF oavsett återinläggning eller ej' );

proc tabulate data=egtask.svov;
class ar hfrakt kon alder_s;
table hfrakt=''*kon*alder_s='Ålder' all='Totalt',ar='År'*n='' /Box=' ' ;
format alder_s age. kon $kon.;
run;
ods excel options(sheet_name='Alla HF individer');
proc tabulate data=egtask.svov_p;
class ar hfrakt kon alder_s;
table hfrakt=''*kon*alder_s='Ålder' all='Totalt',ar='År'*n='' /Box=' ' ;
format alder_s age. kon $kon.;
run;
```

```
ods excel options(sheet_name='Alla HF förstagångare');
proc tabulate data=nyafall(where=(inc=1));
class ar hfrakt kon alder_s;
table hfrakt=''*kon*alder_s='Ålder' all='Totalt',ar='År'*n='' /Box=' ' ;
format alder_s age. kon $kon.;
run;

ods excel close;
```
